# Supplementary material for: Changes in sick notes associated with COVID-19 from 2020 to 2022: a cohort study in 24 million primary care patients in OpenSAFELY-TPP
Source: BMJ Open. 2024 Jul 3;14(7):e080600. doi: 10.1136/bmjopen-2023-080600 (PMC11227761; doi:10.1136/bmjopen-2023-080600)
Supplement: Supplementary data [file bmjopen-2023-080600supp001.pdf]

Supplementary Files

Supplementary Figure 1. Source of first recorded positive SARS-CoV-2 test or COVID-19 diagnosis, by year. Some people have been identified by multiple sources and therefore the percentages within years may not add up to 100%. SGSS = Second Generation Surveillance System.

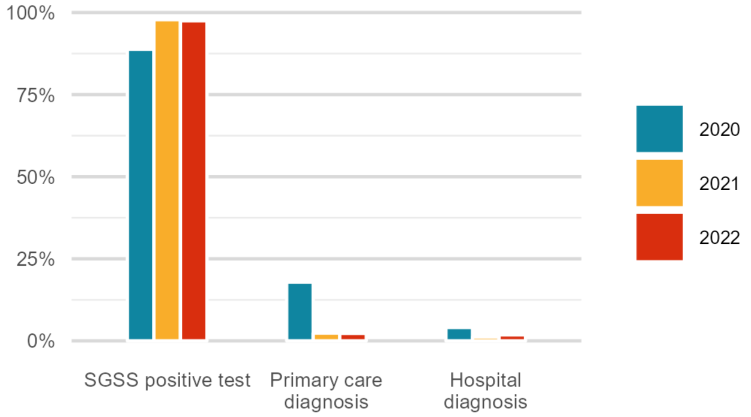

Supplementary Figure 2. Month of first recorded positive SARS-CoV-2 test or COVID-19 diagnosis by year

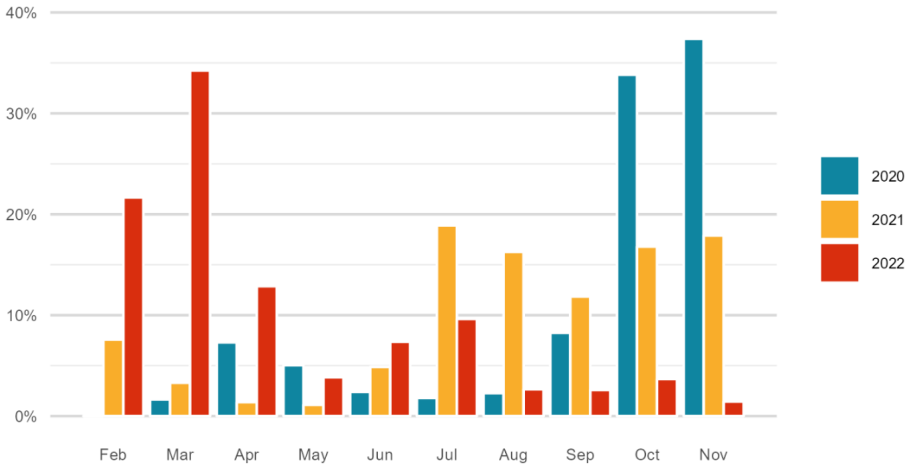

Supplementary Table 1. Select clinical characteristics of general population cohorts, 1 Feb - 30 Nov of each year. General population comparator cohorts were frequency-matched on age, sex, and administrative region. All counts rounded to nearest 7.

	2019	2020		2021		2022	
	General population*	COVID-19 cohort	General population^	COVID-19 cohort	General population^	COVID-19 cohort	General population^
	n (%)	n (%)	n (%)	n (%)	n (%)	n (%)	n (%)
**Total**	3,140,326 (100.0)	365,421 (100.0)	3,439,534 (100.0)	1,206,555 (100.0)	4,571,469 (100.0)	1,321,313 (100.0)	4,818,870 (100.0)
**Health conditions**							
Other chronic respiratory disease	55,265 (1.8)	6650 (1.8)	59,773 (1.7)	15,932 (1.3)	67,361 (1.5)	27,083 (2.0)	90,755 (1.9)
Chronic cardiac disease	79,667 (2.5)	11,039 (3.0)	86,933 (2.5)	26,320 (2.2)	100,527 (2.2)	38,906 (2.9)	127,764 (2.7)
Lung cancer	1442 (0.0)	280 (0.1)	1610 (0.0)	483 (0.0)	1764 (0.0)	1050 (0.1)	2730 (0.1)
Haematological cancer	9212 (0.3)	1316 (0.4)	10,101 (0.3)	3444 (0.3)	12,964 (0.3)	7854 (0.6)	16,142 (0.3)
Other cancer	63,987 (2.0)	7798 (2.1)	69,923 (2.0)	22,351 (1.9)	84,742 (1.9)	39,599 (3.0)	125,146 (2.6)
Chronic liver disease	14,672 (0.5)	2093 (0.6)	16,380 (0.5)	4452 (0.4)	20,860 (0.5)	7637 (0.6)	26,110 (0.5)
Other neurological disease	21,945 (0.7)	2499 (0.7)	23,730 (0.7)	6678 (0.6)	29,827 (0.7)	14,364 (1.1)	35,924 (0.7)
Organ transplant	3339 (0.1)	679 (0.2)	3591 (0.1)	1554 (0.1)	4494 (0.1)	3885 (0.3)	5257 (0.1)
Asplenia	3416 (0.1)	427 (0.1)	3675 (0.1)	1127 (0.1)	4662 (0.1)	2191 (0.2)	5446 (0.1)
HIV	5404 (0.2)	714 (0.2)	6027 (0.2)	1799 (0.1)	7973 (0.2)	3304 (0.3)	8799 (0.2)
Other permanent immunodeficiency	2338 (0.1)	336 (0.1)	2730 (0.1)	1071 (0.1)	3689 (0.1)	1869 (0.1)	4158 (0.1)
Rheumatoid arthritis / SLE / psoriasis	137,536 (4.4)	16,982 (4.6)	150,787 (4.4)	56,203 (4.7)	193,893 (4.2)	71,022 (5.4)	229,537 (4.8)
**Smoking status**							
Current	637,581 (20.3)	47,915 (13.1)	684,236 (19.9)	194,327 (16.1)	895,923 (19.6)	192,381 (14.6)	910,245 (18.9)
Former	802,263 (25.5)	103,810 (28.4)	880,390 (25.6)	358,526 (29.7)	1,189,923 (26.0)	426,041 (32.2)	1,426,432 (29.6)
Never	1,536,297 (48.9)	194,747 (53.3)	1,672,286 (48.6)	581,210 (48.2)	2,185,834 (47.8)	661,794 (50.1)	2,280,509 (47.3)
Missing	164,185 (5.2)	18,942 (5.2)	202,622 (5.9)	72,485 (6.0)	299,789 (6.6)	41,097 (3.1)	201,684 (4.2)

*age, sex and STP frequency matched with 2020 COVID-19 cohort; ^age, sex and STP frequency matched with contemporary COVID-19 cohort; HIV = human immunodeficiency virus; SLE = systemic lupus erythematosus

Supplementary Table 2. Demographic and clinical characteristics of hospitalised cohorts, 1 Feb - 30 Nov of each year. All counts rounded to nearest 7.

	**COVID-19 hospitalised cohorts**			**Pneumonia hospitalised cohort**
	**2020**	**2021**	**2022**	**2019**
	**n (%)**	**n (%)**	**n (%)**	**n (%)**
**Total**	22,015 (100.0)	30,205 (100.0)	34,692 (100.0)	29,673 (100.0)
**Age**				
18-24 y	819 (3.7)	2107 (7.0)	2485 (7.2)	1106 (3.7)
25-34 y	2429 (11.0)	5635 (18.7)	6419 (18.5)	2618 (8.8)
35-44 y	3465 (15.7)	6307 (20.9)	6006 (17.3)	4158 (14.0)
45-54 y	6328 (28.7)	7329 (24.3)	7623 (22.0)	7798 (26.3)
55-64 y	8974 (40.8)	8827 (29.2)	12,166 (35.1)	13,986 (47.1)
**Sex**				
Female	10,059 (45.7)	15,442 (51.1)	20,531 (59.2)	14,294 (48.2)
Male	11,956 (54.3)	14,763 (48.9)	14,161 (40.8)	15,379 (51.8)
**Ethnicity**				
White	12,376 (56.2)	18,305 (60.6)	24,542 (70.7)	19,817 (66.8)
Asian or Asian British	3395 (15.4)	3374 (11.2)	2289 (6.6)	1589 (5.4)
Black	1008 (4.6)	1260 (4.2)	805 (2.3)	553 (1.9)
Mixed	343 (1.6)	532 (1.8)	406 (1.2)	217 (0.7)
Other	532 (2.4)	623 (2.1)	511 (1.5)	273 (0.9)
Unknown	4361 (19.8)	6118 (20.3)	6146 (17.7)	7217 (24.3)
**IMD**				
1 (most deprived)	7077 (32.1)	10,199 (33.8)	9478 (27.3)	8974 (30.2)
2	5040 (22.9)	6839 (22.6)	7525 (21.7)	6636 (22.4)
3	3962 (18.0)	5446 (18.0)	6951 (20.0)	5775 (19.5)
4	3269 (14.8)	4319 (14.3)	5957 (17.2)	4662 (15.7)
5 (least deprived)	2667 (12.1)	3395 (11.2)	4774 (13.8)	3626 (12.2)
**Region**				
East	3885 (17.6)	5341 (17.7)	7238 (20.9)	5866 (19.8)
East Midlands	4375 (19.9)	6090 (20.2)	6776 (19.5)	5698 (19.2)
London	1680 (7.6)	1715 (5.7)	1848 (5.3)	1624 (5.5)
North East	1568 (7.1)	2044 (6.8)	2219 (6.4)	1715 (5.8)
North West	2457 (11.2)	3339 (11.1)	3542 (10.2)	2926 (9.9)
South East	854 (3.9)	1421 (4.7)	2359 (6.8)	1701 (5.7)
South West	1631 (7.4)	3080 (10.2)	4326 (12.5)	3857 (13.0)
West Midlands	1547 (7.0)	2037 (6.7)	1771 (5.1)	1463 (4.9)
Yorkshire & The Humber	4025 (18.3)	5145 (17.0)	4613 (13.3)	4816 (16.2)
**Health conditions**				
Obesity	9870 (44.8)	12,600 (41.7)	11,452 (33.0)	9877 (33.3)
Hypertension	6594 (30.0)	6552 (21.7)	8435 (24.3)	8386 (28.3)
Diabetes	5726 (26.0)	5817 (19.3)	6846 (19.7)	6146 (20.7)
Asthma	4907 (22.3)	6755 (22.4)	8694 (25.1)	7581 (25.5)
Other chronic respiratory disease	1645 (7.5)	1722 (5.7)	3304 (9.5)	5460 (18.4)
Chronic cardiac disease	2324 (10.6)	2198 (7.3)	3780 (10.9)	4095 (13.8)
Lung cancer	154 (0.7)	126 (0.4)	301 (0.9)	735 (2.5)
Haematological cancer	371 (1.7)	469 (1.6)	1162 (3.3)	882 (3.0)
Other cancer	1323 (6.0)	1358 (4.5)	3199 (9.2)	3325 (11.2)
Chronic liver disease	623 (2.8)	595 (2.0)	1582 (4.6)	1477 (5.0)
Other neurological disease	651 (3.0)	595 (2.0)	1841 (5.3)	1260 (4.2)
Organ transplant	280 (1.3)	455 (1.5)	1253 (3.6)	490 (1.7)

Asplenia	77 (0.3)	91 (0.3)	203 (0.6)	189 (0.6)
HIV	14 (0.1)	14 (0.0)	49 (0.1)	21 (0.1)
Other permanent immunodeficiency	49 (0.2)	70 (0.2)	196 (0.6)	91 (0.3)
Rheumatoid arthritis / SLE / Psoriasis	1505 (6.8)	1967 (6.5)	3066 (8.8)	2569 (8.7)
**Smoking status**				
Current	3171 (14.4)	4788 (15.9)	9093 (26.2)	11,039 (37.2)
Former	7791 (35.4)	10,654 (35.3)	11,277 (32.5)	9233 (31.1)
Never	10,752 (48.8)	14,021 (46.4)	13,636 (39.3)	9058 (30.5)
Missing	301 (1.4)	742 (2.5)	679 (2.0)	336 (1.1)

HIV = human immunodeficiency virus; SLE = systemic lupus erythematosus

Supplementary Table 3. Number and percentage of people with a sick note in each cohort by demographic categories. The denominator is all people within each category. All counts rounded to the nearest 7.

	2021	2020		2021		2022	
	General population*	COVID-19 cohort	General population^	COVID-19 cohort	General population^	COVID-19 cohort	General population^
	n (%)	n (%)	n (%)	n (%)	n (%)	n (%)	n (%)
**Total**	181,167 (5.8)	34,377 (9.4)	153,447 (4.5)	102,949 (8.5)	259,812 (5.7)	152,859 (11.6)	273,889 (5.7)
**Age**							
18-24 y	23,807 (4.8)	1687 (2.7)	17,556 (3.2)	9765 (5.0)	31,458 (4.5)	10,262 (9.2)	16,499 (4.4)
25-34 y	37,212 (5.6)	5264 (6.8)	30,051 (4.1)	20,307 (7.7)	53,704 (5.4)	29,078 (11.3)	48,804 (5.3)
35-44 y	36,575 (5.7)	7413 (9.9)	30,919 (4.4)	25,732 (8.7)	63,924 (5.7)	35,672 (11.4)	62,881 (5.5)
45-54 y	46,347 (6.4)	10,843 (12.9)	40,586 (5.2)	27,489 (10.1)	66,381 (6.4)	41,195 (12.5)	74,326 (6.2)
55-64 y	37,226 (6.0)	9177 (13.5)	34,335 (5.1)	19,649 (10.9)	44,345 (6.1)	36,652 (11.8)	71,372 (6.0)
**Sex**							
Female	114,191 (6.6)	23,191 (11.5)	100,037 (5.3)	65,037 (10.1)	164,451 (6.7)	106,610 (12.9)	193,816 (6.5)
Male	66,976 (4.8)	11,186 (6.8)	53,410 (3.5)	37,912 (6.8)	95,361 (4.5)	46,249 (9.3)	80,080 (4.4)
**Ethnicity**							
White	118,272 (6.2)	20,622 (9.7)	100,681 (4.8)	67,522 (8.6)	173,194 (6.0)	112,063 (11.6)	193,599 (5.9)
Mixed	11,060 (4.9)	4662 (10.3)	10,409 (4.1)	8169 (11.9)	16,219 (5.1)	7000 (13.4)	16,310 (5.4)
Asian / Asian British	3955 (5.9)	1078 (13.3)	3325 (4.3)	2632 (12.3)	6160 (6.0)	2576 (14.0)	6895 (6.5)
Black	2016 (5.8)	420 (9.8)	1764 (4.4)	1274 (9.1)	3108 (5.5)	1624 (11.5)	3381 (5.8)
Other	1820 (3.0)	490 (8.9)	1596 (2.3)	1211 (8.1)	3241 (3.2)	1701 (8.8)	3689 (3.5)
Unknown	44,051 (5.3)	7112 (8.0)	35,672 (4.0)	22,127 (7.4)	57,890 (5.1)	27,888 (11.1)	50,022 (5.2)
**Region**							
East Midlands	21693 (5.0)	4767 (9.5)	17,423 (3.7)	17,577 (7.3)	44,625 (4.9)	34,139 (10.4)	59,633 (5.0)
East	37,121 (5.8)	6608 (8.8)	31,213 (4.4)	20426 (9.0)	51,457 (6.0)	27,720 (12.5)	50,442 (6.2)
London	4998 (3.4)	1148 (6.7)	3801 (2.4)	4053 (7.1)	7084 (3.4)	5957 (8.4)	9163 (3.6)
North East	16,373 (6.3)	3087 (10.3)	14,329 (5.1)	6979 (9.8)	17,724 (6.6)	7833 (13.3)	15,092 (7.0)
North West	28,168 (6.6)	5313 (10.7)	25,025 (5.4)	12,558 (9.7)	33,950 (6.9)	14,805 (13.6)	27,965 (7.0)

South East	4676 (4.6)	987 (8.3)	3913 (3.5)	4599 (6.5)	12,425 (4.7)	9996 (10.0)	17,430 (4.8)
South West	11,354 (5.1)	2254 (8.8)	9401 (3.9)	11,382 (7.1)	31,787 (5.2)	25,158 (11.0)	44,107 (5.3)
West Midlands	10,941 (6.2)	2002 (9.7)	8939 (4.6)	5481 (10.6)	12,229 (6.3)	5705 (14.0)	10,094 (6.8)
Yorkshire & The Humber	45,843 (6.2)	8204 (9.6)	39,403 (4.9)	19,887 (10.1)	48,524 (6.4)	21,539 (13.1)	39,956 (6.6)
**IMD quintile**							
1 (most deprived)	59,052 (7.4)	9989 (10.2)	50,148 (5.7)	28,728 (11.1)	77,714 (7.6)	33,229 (15.7)	72,597 (7.8)
2	39,767 (6.3)	7581 (9.9)	33,586 (4.9)	22,827 (9.4)	57,729 (6.2)	33,320 (13.2)	61,446 (6.3)
3	32,417 (5.4)	6503 (9.4)	27,699 (4.2)	20,083 (8.1)	49,301 (5.3)	32,578 (11.2)	55,762 (5.3)
4	27,853 (4.8)	5698 (8.9)	23,506 (3.7)	17,087 (7.3)	41,846 (4.8)	29,288 (10.2)	46,494 (4.8)
5 (least deprived)	22,078 (4.2)	4606 (8.1)	18,494 (3.2)	14,224 (6.4)	33,222 (4.1)	24,444 (8.8)	37,597 (4.2)

*age, sex and STP frequency matched with 2020 COVID-19 cohort; ^age, sex and STP frequency matched with contemporary COVID-19 cohort

Supplementary Table 4. Number of people issued a sick note and rate of first sick note per 100 person-months for hospitalised cohorts by demographic categories. The denominator for the percentages is all people within each category. All counts rounded to the nearest 7.

	**COVID-19 hospitalised cohorts**						**Pneumonia hospitalised cohort 2019**	
	**2020**		**2021**		**2022**		**2019**	
	**n (%)**	**Rate per 100 person-months (95%CI)**	**n (%)**	**Rate per 100 person-months (95%CI)**	**n (%)**	**Rate per 100 person-months (95%CI)**	**n (%)**	**Rate per 100 person-months (95%CI)**
**Total**	4606 (20.9)	6.78 (6.59 - 6.98)	7231 (23.9)	7.19 (7.03 - 7.36)	6664 (19.2)	4.13 (4.03 - 4.22)	7448 (25.1)	7.17 (7.01 - 7.34)
**Age**								
18-24 y	91 (11.1)	3.58 (2.84 - 4.31)	336 (15.9)	4.26 (3.80 - 4.71)	392 (15.8)	3.02 (2.72 - 3.31)	266 (24.1)	6.29 (5.53 - 7.04)
25-34 y	399 (16.4)	4.98 (4.49 - 5.47)	1050 (18.6)	5.27 (4.95 - 5.58)	1029 (16.0)	3.04 (2.86 - 3.23)	798 (30.5)	8.46 (7.88 - 9.05)
35-44 y	791 (18.1)	6.88 (6.41 - 7.36)	1589 (25.2)	7.75 (7.37 - 8.13)	1218 (20.3)	4.14 (3.90 - 4.37)	1253 (30.1)	8.66 (8.18 - 9.13)
45-54 y	1498 (23.7)	7.50 (7.12 - 7.88)	2107 (28.7)	9.07 (8.68 - 9.46)	1722 (22.6)	5.16 (4.92 - 5.41)	2100 (26.9)	7.76 (7.42 - 8.09)
55-64 y	1834 (20.4)	7.09 (6.76 - 7.41)	2149 (24.3)	7.42 (7.11 - 7.73)	2310 (19.0)	4.45 (4.27 - 4.63)	3031 (21.7)	6.23 (6.01 - 6.46)
**Sex**								
Female	2212 (22.0)	7.29 (6.99 - 7.60)	3430 (22.2)	6.64 (6.42 - 6.86)	3731 (18.2)	3.74 (3.62 - 3.86)	3556 (24.9)	7.00 (6.77 - 7.23)
Male	2394 (20.0)	6.37 (6.12 - 6.63)	3801 (25.7)	7.78 (7.53 - 8.03)	2940 (20.8)	4.75 (4.58 - 4.92)	3899 (25.4)	7.36 (7.12 - 7.59)
**Ethnicity**								
White	2611 (21.1)	6.99 (6.73 - 7.26)	4459 (24.4)	7.63 (7.41 - 7.86)	4704 (19.2)	4.12 (4.00 - 4.23)	4914 (24.8)	7.08 (6.88 - 7.28)
Mixed	651 (19.2)	6.28 (5.80 - 6.76)	721 (21.4)	5.36 (4.97 - 5.75)	427 (18.7)	4.07 (3.68 - 4.45)	350 (22.0)	5.76 (5.16 - 6.37)
Asian / Asian British	245 (24.3)	6.79 (5.94 - 7.64)	336 (26.7)	8.30 (7.41 - 9.18)	175 (21.7)	4.78 (4.07 - 5.49)	147 (26.6)	7.38 (6.19 - 8.57)
Black	77 (22.4)	6.79 (5.28 - 8.31)	112 (21.1)	6.28 (5.12 - 7.44)	77 (19.0)	3.89 (3.02 - 4.76)	56 (25.8)	7.17 (5.29 - 9.04)
Other	105 (19.7)	5.41 (4.37 - 6.44)	119 (19.1)	4.81 (3.94 - 5.67)	84 (16.4)	3.57 (2.81 - 4.34)	63 (23.1)	7.27 (5.48 - 9.06)
Unknown	910 (20.9)	6.74 (6.30 - 7.18)	1484 (24.3)	7.30 (6.92 - 7.67)	1204 (19.6)	4.18 (3.95 - 4.42)	1925 (26.7)	7.79 (7.44 - 8.13)
**Region**								
East Midlands	819 (21.1)	5.79 (5.39 - 6.18)	1246 (23.3)	7.35 (6.94 - 7.75)	1358 (18.8)	3.88 (3.67 - 4.09)	1512 (25.8)	7.30 (6.94 - 7.67)
East	854 (19.5)	6.39 (5.96 - 6.82)	1428 (23.4)	7.02 (6.65 - 7.38)	1372 (20.2)	4.48 (4.24 - 4.71)	1449 (25.4)	7.35 (6.97 - 7.73)
London	287 (17.1)	4.26 (3.77 - 4.75)	329 (19.2)	5.31 (4.74 - 5.89)	294 (15.9)	3.17 (2.81 - 3.54)	350 (21.6)	5.95 (5.33 - 6.57)
North East	322 (20.5)	7.79 (6.94 - 8.63)	497 (24.3)	6.91 (6.30 - 7.51)	392 (17.7)	3.81 (3.43 - 4.18)	420 (24.5)	7.11 (6.43 - 7.79)
North West	581 (23.6)	9.11 (8.37 - 9.85)	861 (25.8)	7.49 (6.99 - 7.99)	721 (20.4)	4.55 (4.22 - 4.88)	735 (25.1)	7.32 (6.79 - 7.85)
South East	168 (19.7)	5.20 (4.42 - 5.99)	329 (23.2)	6.87 (6.13 - 7.62)	427 (18.1)	3.65 (3.30 - 3.99)	434 (25.5)	7.16 (6.48 - 7.83)

South West	413 (25.3)	8.04 (7.27 - 8.82)	735 (23.9)	8.69 (8.06 - 9.32)	819 (18.9)	3.91 (3.64 - 4.18)	994 (25.8)	7.14 (6.69 - 7.58)
West Midlands	301 (20.7)	6.28 (5.57 - 6.99)	497 (24.4)	6.71 (6.12 - 7.30)	357 (20.2)	4.66 (4.18 - 5.15)	343 (23.4)	6.69 (5.98 - 7.40)
Yorkshire & The Humber	854 (21.2)	8.58 (8.00 - 9.15)	1316 (25.6)	7.45 (7.05 - 7.85)	938 (20.3)	4.64 (4.34 - 4.94)	1211 (25.1)	7.35 (6.93 - 7.76)
**IMD quintile**								
1 (most deprived)	1337 (18.9)	6.50 (6.16 - 6.85)	2352 (23.1)	6.54 (6.27 - 6.80)	1743 (20.6)	4.01 (3.82 - 4.20)	1967 (21.9)	6.13 (5.86 - 6.40)
2	1043 (20.7)	6.69 (6.28 - 7.10)	1645 (24.1)	7.35 (7.00 - 7.70)	1449 (19.3)	4.18 (3.96 - 4.39)	1673 (25.2)	7.16 (6.82 - 7.50)
3	896 (22.6)	7.22 (6.75 - 7.69)	1379 (25.3)	7.74 (7.33 - 8.14)	1330 (19.1)	4.05 (3.83 - 4.26)	1512 (26.2)	7.51 (7.13 - 7.88)
4	756 (23.1)	7.29 (6.77 - 7.81)	1057 (24.5)	7.71 (7.24 - 8.17)	1183 (19.9)	4.24 (3.99 - 4.48)	1288 (27.6)	8.08 (7.64 - 8.52)
5 (least deprived)	574 (21.5)	6.40 (5.88 - 6.93)	798 (23.5)	7.51 (6.99 - 8.03)	952 (19.9)	4.22 (3.95 - 4.49)	1015 (28.0)	8.25 (7.75 - 8.76)

Supplementary Table 5. Crude and adjusted HRs for receipt of first sick note by follow-up period post-index date among general population cohorts

Comparison and follow-up period post-index date	Sick note rate per 100 person-months (95%CI)		Crude HR (95%CI)	Age-sex adjusted HR (95%CI)
	COVID-19 cohort	General population		
**2020 COVID-19 cohort vs 2019 general population**				
0-30 days	9.35 (9.24 - 9.46)	1.99 (1.98 - 2.01)	4.59 (4.52 - 4.65)	4.77 (4.70 - 4.84)
0-90 days	6.20 (6.14 - 6.27)	1.48 (1.47 - 1.48)	3.59 (3.55 - 3.64)	3.69 (3.64 - 3.73)
0-150 days	5.39 (5.33 - 5.45)	1.31 (1.30 - 1.32)	3.35 (3.31 - 3.39)	3.41 (3.37 - 3.45)
Entire follow-up period	4.88 (4.83 - 4.93)	1.21 (1.20 - 1.22)	3.20 (3.16 - 3.24)	3.23 (3.20 - 3.27)
**2020 COVID-19 cohort vs 2020 general population**				
0-30 days	9.35 (9.24 - 9.46)	1.57 (1.55 - 1.58)	5.83 (5.74 - 5.92)	6.14 (6.05 - 6.24)
0-90 days	6.20 (6.14 - 6.27)	1.12 (1.11 - 1.13)	4.65 (4.59 - 4.71)	4.83 (4.77 - 4.89)
0-150 days	5.39 (5.33 - 5.45)	0.99 (0.99 - 1.00)	4.35 (4.30 - 4.40)	4.46 (4.40 - 4.51)
Entire follow-up period	4.88 (4.83 - 4.93)	0.93 (0.93 - 0.93)	4.13 (4.08 - 4.18)	4.19 (4.14 - 4.24)
**2021 COVID-19 cohort vs 2019 general population**				
0-30 days	6.51 (6.46 - 6.56)	1.99 (1.98 - 2.01)	3.24 (3.21 - 3.28)	3.33 (3.29 - 3.37)
0-90 days	3.50 (3.48 - 3.52)	1.48 (1.47 - 1.48)	2.27 (2.25 - 2.29)	2.39 (2.37 - 2.41)
0-150 days	2.91 (2.90 - 2.93)	1.31 (1.30 - 1.32)	2.06 (2.04 - 2.07)	2.17 (2.15 - 2.18)
Entire follow-up period	2.66 (2.64 - 2.67)	1.21 (1.20 - 1.22)	1.96 (1.95 - 1.98)	2.04 (2.03 - 2.06)
**2021 COVID-19 cohort vs 2021 general population**				
0-30 days	6.51 (6.46 - 6.56)	1.97 (1.96 - 1.99)	3.27 (3.24 - 3.31)	3.43 (3.40 - 3.47)
0-90 days	3.50 (3.48 - 3.52)	1.47 (1.46 - 1.48)	2.29 (2.28 - 2.31)	2.40 (2.38 - 2.42)
0-150 days	2.91 (2.90 - 2.93)	1.32 (1.31 - 1.33)	2.07 (2.06 - 2.09)	2.17 (2.16 - 2.19)
Entire follow-up period	2.66 (2.64 - 2.67)	1.23 (1.22 - 1.23)	1.97 (1.96 - 1.99)	2.06 (2.04 - 2.07)
**2022 COVID-19 cohort vs 2019 general population**				
0-30 days	4.87 (4.83 - 4.91)	1.99 (1.98 - 2.01)	2.46 (2.43 - 2.48)	2.14 (2.11 - 2.17)
0-90 days	2.57 (2.55 - 2.58)	1.48 (1.47 - 1.48)	1.79 (1.77 - 1.81)	1.63 (1.62 - 1.65)
0-150 days	2.05 (2.04 - 2.06)	1.31 (1.30 - 1.32)	1.65 (1.64 - 1.66)	1.53 (1.52 - 1.55)
Entire follow-up period	1.73 (1.72 - 1.73)	1.21 (1.20 - 1.22)	1.56 (1.55 - 1.57)	1.46 (1.45 - 1.47)
**2022 COVID-19 cohort vs 2022 general population**				

Comparison and follow-up period post-index date	Sick note rate per 100 person-months (95%CI)		Crude HR (95%CI)	Age-sex adjusted HR (95%CI)
	COVID-19 cohort	General population		
0-30 days	4.87 (4.83 - 4.91)	2.09 (2.07 - 2.10)	2.35 (2.32 - 2.37)	2.35 (2.33 - 2.38)
0-90 days	2.57 (2.55 - 2.58)	1.53 (1.52 - 1.54)	1.73 (1.71 - 1.74)	1.73 (1.72 - 1.74)
0-150 days	2.05 (2.04 - 2.06)	1.34 (1.39 - 1.35)	1.62 (1.60 - 1.63)	1.62 (1.61 - 1.63)
Entire follow-up period	1.73 (1.72 - 1.73)	1.23 (1.22 - 1.23)	1.55 (1.54 - 1.56)	1.55 (1.54 - 1.56)

^General population age, sex and STP frequency matched.

*Fully adjusted models include age, sex, IMD quintile, region, ethnicity, obesity, smoking status, hypertension, diabetes, chronic respiratory disease, asthma, chronic cardiac disease, lung cancer, haematological cancer, other cancer, chronic liver disease, other neurological disease, organ transplant, asplenia, HIV, permanent immunodeficiency, and rheumatoid arthritis/systemic lupus erythematosus/psoriasis.

Supplementary Table 6. Crude and adjusted HRs for receipt of first sick note for comparisons of hospitalised cohorts, overall and by follow-up time post-diagnosis

Time period	Sick note rate per 100 person-months (95%CI)		Crude HR (95%CI)	Age-sex adjusted HR (95%CI)	Fully adjusted HR* (95%CI)
	COVID-19 cohort	Comparator cohort			
**2020 COVID-19 cohort vs 2019 pneumonia cohort**					
0-30 days	18.84 (18.18 - 19.49)	22.06 (21.46 - 22.66)	0.85 (0.81 - 0.88)	0.83 (0.78 - 0.88)	0.72 (0.68 - 0.77)
0-90 days	11.44 (11.09 - 11.79)	12.47 (12.17 - 12.76)	0.86 (0.83 - 0.89)	0.83 (0.78 - 0.88)	0.74 (0.69 - 0.78)
0-150 days	8.66 (8.40 - 8.92)	9.28 (9.06 - 9.49)	0.88 (0.85 - 0.92)	0.84 (0.79 - 0.88)	0.75 (0.71 - 0.80)
Overall	6.78 (6.59 - 6.98)	7.17 (7.01 - 7.34)	0.90 (0.86 - 0.93)	0.85 (0.80 - 0.89)	0.77 (0.73 - 0.81)
**2021 COVID-19 cohort vs 2019 pneumonia cohort**					
0-30 days	21.39 (20.81 - 21.96)	22.06 (21.46 - 22.66)	0.97 (0.93 - 1.00)	0.98 (0.92 - 1.03)	0.83 (0.78 - 0.88)
0-90 days	11.65 (11.37 - 11.93)	12.47 (12.17 - 12.76)	0.93 (0.90 - 0.96)	0.92 (0.88 - 0.97)	0.81 (0.77 - 0.85)
0-150 days	9.03 (8.82 - 9.24)	9.28 (9.06 - 0.49)	0.93 (0.90 - 0.96)	0.91 (0.87 - 0.96)	0.80 (0.77 - 0.85)
Overall	7.19 (7.03 - 7.36)	7.17 (7.01 - 7.34)	0.94 (0.91 - 0.97)	0.92 (0.87 - 0.96)	0.81 (0.77 - 0.86)
**2022 COVID-19 cohort vs 2019 pneumonia cohort**					
0-30 days	12.92 (12.52 - 13.32)	22.06 (21.46 - 22.66)	0.59 (0.57 - 0.62)	0.58 (0.54 - 0.61)	0.56 (0.53 - 0.60)
0-90 days	7.05 (6.87 - 7.24)	12.47 (12.17 - 12.75)	0.61 (0.59 - 0.62)	0.59 (0.56 - 0.62)	0.58 (0.56 - 0.61)
0-150 days	5.22 (5.09 - 5.35)	9.28 (9.06 - 9.49)	0.63 (0.61 - 0.65)	0.61 (0.58 - 0.64)	0.60 (0.58 - 0.63)
Overall	4.13 (4.03 - 4.22)	7.17 (7.01 - 7.34)	0.65 (0.63 - 0.67)	0.63 (0.60 - 0.66)	0.62 (0.59 - 0.65)

*Fully adjusted models include age, sex, IMD quintile, region, ethnicity, obesity, smoking status, hypertension, diabetes, chronic respiratory disease, asthma, chronic cardiac disease, lung cancer, haematological cancer, other cancer, chronic liver disease, other neurological disease, organ transplant, asplenia, HIV, permanent immunodeficiency, and rheumatoid arthritis/systemic lupus erythematosus/psoriasis.

Supplementary Table 7. Crude and adjusted hazard ratio for first sick note for all comparisons, stratified by age group.

	COVID-19 cohort vs 2019 general population		COVID-19 cohort vs contemporary general population	
	Crude HR (95%CI)	Fully adjusted HR (95%CI)	Crude HR (95%CI)	Fully adjusted HR (95%CI)
**2020 COVID-19 cohort**				
18-24 y	1.31 (1.25 - 1.38)	1.38 (1.31 - 1.45)	2.01 (1.91 - 2.12)	2.05 (1.95 - 2.16)
25-34 y	2.44 (2.37 - 2.51)	2.47 (2.40 - 2.54)	3.31 (3.22 - 3.41)	3.29 (3.19 - 3.39)
35-44 y	3.47 (3.38 - 3.56)	3.45 (3.36 - 3.54)	4.46 (4.35 - 4.58)	4.37 (4.26 - 4.48)
45-54 y	3.83 (3.75 - 3.91)	3.77 (3.69 - 3.85)	4.77 (4.67 - 4.87)	4.64 (4.54 - 4.75)
55-64 y	4.05 (3.95 - 4.14)	3.89 (3.80 - 3.98)	4.80 (4.69 - 4.91)	4.59 (4.48 - 4.70)
**2021 COVID-19 cohort**				
18-24 y	1.22 (1.19 - 1.25)	1.06 (1.00 - 1.12)	1.25 (1.23 - 1.28)	1.24 (1.22 - 1.27)
25-34 y	1.71 (1.68 - 1.74)	1.31 (1.28 - 1.35)	1.73 (1.71 - 1.76)	1.68 (1.66 - 1.71)
35-44 y	2.16 (2.13 - 2.19)	1.75 (1.72 - 1.78)	2.13 (2.10 - 2.17)	2.10 (2.07 - 2.13)
45-54 y	2.26 (2.23 - 2.29)	2.19 (2.15 - 2.22)	2.27 (2.24 - 2.30)	2.26 (2.23 - 2.29)
55-64 y	2.51 (2.47 - 2.56)	2.36 (2.32 - 2.39)	2.48 (2.44 - 2.52)	2.41 (2.37 - 2.45)
**2022 COVID-19 cohort**				
18-24 y	1.41 (1.38 - 1.45)	1.44 (1.40 - 1.47)	1.53 (1.49 - 1.57)	1.47 (1.43 - 1.51)
25-34 y	1.49 (1.47 - 1.51)	1.57 (1.54 - 1.59)	1.55 (1.52 - 1.57)	1.54 (1.52 - 1.57)
35-44 y	1.52 (1.50 - 1.54)	1.60 (1.57 - 1.63)	1.53 (1.51 - 1.55)	1.55 (1.53 - 1.57)
45-54 y	1.55 (1.53 - 1.57)	1.64 (1.61 - 1.66)	1.57 (1.55 - 1.59)	1.60 (1.58 - 1.62)
55-64 y	1.60 (1.57 - 1.62)	1.74 (1.71 - 1.77)	1.56 (1.54 - 1.59)	1.63 (1.61 - 1.65)

	COVID-19 hospitalised cohort vs 2019 pneumonia hospitalised cohort	
	Crude HR (95%CI)	Fully adjusted HR (95%CI)
**2020 COVID-19 hospitalised cohort**		
18-24 y	0.50 (0.39 - 0.63)	0.48 (0.37 - 0.61)
25-34 y	0.54 (0.48 - 0.61)	0.54 (0.47 - 0.61)
35-44 y	0.77 (0.71 - 0.84)	0.73 (0.66 - 0.80)
45-54 y	0.94 (0.88 - 1.00)	0.81 (0.76 - 0.88)

55-64 y	0.50 (0.39 - 0.63)	0.94 (0.88 - 0.99)	
**2021 COVID-19 hospitalised cohort**			
18-24 y	0.64 (0.54 - 0.75)	0.61 (0.51 - 0.72)	
25-34 y	0.57 (0.52 - 0.63)	0.57 (0.51 - 0.63)	
35-44 y	0.82 (0.76 - 0.88)	0.74 (0.68 - 0.80)	
45-54 y	1.09 (1.03 - 1.16)	0.94 (0.88 - 1.01)	
55-64 y	1.16 (1.09 - 1.22)	0.61 (0.51 - 0.72)	
**2022 COVID-19 hospitalised cohort**			
18-24 y	0.54 (0.46 - 0.63)	0.54 (0.46 - 0.64)	
25-34 y	0.42 (0.39 - 0.47)	0.44 (0.39 - 0.48)	
35-44 y	0.56 (0.52 - 0.61)	0.56 (0.52 - 0.61)	
45-54 y	0.73 (0.68 - 0.78)	0.72 (0.68 - 0.77)	
55-64 y	0.76 (0.72 - 0.80)	0.78 (0.74 - 0.82)	

*Fully adjusted models include age, sex, IMD quintile, region, ethnicity, obesity, smoking status, hypertension, diabetes, chronic respiratory disease, asthma, chronic cardiac disease, lung cancer, haematological cancer, other cancer, chronic liver disease, other neurological disease, organ transplant, asplenia, HIV, permanent immunodeficiency, and rheumatoid arthritis/systemic lupus erythematosus/psoriasis.

Supplementary Table 8. Crude and adjusted hazard ratio of first sick note for all comparisons, stratified by sex

	**COVID-19 cohort vs 2019 general population**		**COVID-19 cohort vs contemporary general population**	
	**Crude HR (95%CI)**	**Fully adjusted HR (95%CI)**	**Crude HR (95%CI)**	**Fully adjusted HR (95%CI)**
**2020 COVID-19 cohort**				
Female	3.39 (3.34 - 3.44)	3.40 (3.35 - 3.45)	4.22 (4.16 - 4.29)	4.22 (4.15 - 4.28)
Male	2.86 (2.80 - 2.92)	2.84 (2.78 - 2.89)	3.91 (3.83 - 4.00)	3.79 (3.71 - 3.87)
**2021 COVID-19 cohort**				
Female	2.08 (2.06 - 2.10)	2.08 (2.06 - 2.10)	2.01 (1.99 - 2.03)	2.01 (2.00 - 2.03)
Male	1.84 (1.82 - 1.86)	1.93 (1.90 - 1.95)	1.93 (1.91 - 1.95)	1.95 (1.93 - 1.98)
**2022 COVID-19 cohort**				
Female	1.54 (1.52 - 1.55)	1.52 (1.51 - 1.54)	1.53 (1.51 - 1.54)	1.53 (1.52 - 1.54)
Male	1.50 (1.48 - 1.52)	1.61 (1.59 - 1.64)	1.60 (1.58 - 1.62)	1.67 (1.65 - 1.69)

	**COVID-19 hospitalised cohort vs 2019 pneumonia hospitalised cohort**	
	**Crude HR (95%CI)**	**Fully adjusted HR (95%CI)**
**2020 COVID-19 hospitalised cohort**		
Female	0.95 (0.91 - 1.01)	0.78 (0.72 - 0.85)
Male	0.85 (0.81 - 0.89)	0.75 (0.69 - 0.81)
**2021 COVID-19 hospitalised cohort**		
Female	0.87 (0.83 - 0.92)	0.76 (0.71 - 0.82)
Male	1.02 (0.97 - 1.06)	0.88 (0.82 - 0.94)
**2022 COVID-19 hospitalised cohort**		
Female	0.61 (0.59 - 0.64)	0.57 (0.53 - 0.61)
Male	0.70 (0.67 - 0.74)	0.70 (0.66 - 0.75)

*Fully adjusted models include age, sex, IMD quintile, region, ethnicity, obesity, smoking status, hypertension, diabetes, chronic respiratory disease, asthma, chronic cardiac disease, lung cancer, haematological cancer, other cancer, chronic liver disease, other neurological disease, organ transplant, asplenia, HIV, permanent immunodeficiency, and rheumatoid arthritis/systemic lupus erythematosus/psoriasis.

Supplementary Table 9. Crude and adjusted hazard ratio of first sick note for all comparisons, stratified by ethnicity

	**COVID-19 cohort vs 2019 general population**		**COVID-19 cohort vs contemporary general population**	
	**Crude HR (95%CI)**	**Fully adjusted HR (95%CI)**	**Crude HR (95%CI)**	**Fully adjusted HR (95%CI)**
**2020 COVID-19 cohort**				
White	3.13 (3.08 - 3.18)	3.13 (3.08 - 3.18)	4.05 (3.99 - 4.11)	4.01 (3.95 - 4.08)
Asian or Asian British	3.71 (3.59 - 3.84)	3.60 (3.48 - 3.73)	4.44 (4.30 - 4.60)	4.31 (4.16 - 4.47)
Black	3.81 (3.56 - 4.08)	3.76 (3.50 - 4.03)	5.12 (4.78 - 5.49)	4.95 (4.60 - 5.32)
Mixed	3.04 (2.73 - 3.38)	2.99 (2.68 - 3.33)	4.00 (3.59 - 4.45)	3.89 (3.48 - 4.34)
Other	5.33 (4.83 - 5.89)	4.54 (4.08 - 5.05)	6.74 (6.09 - 7.46)	5.89 (5.28 - 6.56)
Not stated				
**2021 COVID-19 cohort**				
White	1.89 (1.87 - 1.91)	1.94 (1.92 - 1.96)	1.91 (1.90 - 1.93)	1.94 (1.92 - 1.96)
Asian or Asian British	2.84 (2.76 - 2.92)	2.93 (2.84 - 3.02)	2.69 (2.62 - 2.76)	2.62 (2.55 - 2.70)
Black	2.59 (2.47 - 2.72)	2.88 (2.73 - 3.04)	2.54 (2.42 - 2.66)	2.62 (2.50 - 2.74)
Mixed	2.01 (1.87 - 2.15)	2.14 (1.98 - 2.30)	2.09 (1.95 - 2.23)	2.08 (1.95 - 2.23)
Other	3.42 (3.18 - 3.67)	3.22 (2.97 - 3.49)	3.23 (3.03 - 3.45)	2.90 (2.71 - 3.11)
Not stated				
**2022 COVID-19 cohort**				
White	1.46 (1.45 - 1.47)	1.48 (1.47 - 1.50)	1.49 (1.48 - 1.50)	2.10 (2.04 - 2.16)
Asian or Asian British	2.21 (2.15 - 2.28)	2.48 (2.40 - 2.58)	1.97 (1.91 - 2.02)	1.69 (1.61 - 1.77)
Black	1.94 (1.85 - 2.04)	1.88 (1.77 - 1.99)	1.71 (1.64 - 1.80)	1.60 (1.51 - 1.71)
Mixed	1.57 (1.47 - 1.68)	1.66 (1.54 - 1.79)	1.54 (1.45 - 1.64)	1.98 (1.86 - 2.10)
Other	2.32 (2.16 - 2.48)	2.23 (2.06 - 2.42)	1.93 (1.82 - 2.04)	1.61 (1.59 - 1.63)
Not stated	1.62 (1.59 - 1.64)	1.54 (1.51 - 1.57)	1.63 (1.61 - 1.66)	2.10 (2.04 - 2.16)
	**COVID-19 hospitalised cohort vs 2019 pneumonia hospitalised cohort**			
	**Crude HR (95%CI)**	**Fully adjusted HR (95%CI)**		
**2020 COVID-19 hospitalised cohort**				
White	0.93 (0.89 - 0.97)	0.78 (0.72 - 0.83)		

Asian or Asian British	0.98 (0.86 - 1.12)	0.90 (0.76 - 1.08)	
Black	0.97 (0.79 - 1.19)	0.80 (0.60 - 1.07)	
Mixed	0.96 (0.68 - 1.35)	0.58 (0.35 - 0.94)	
Other	0.83 (0.61 - 1.14)	0.77 (0.51 - 1.18)	
Not stated	0.83 (0.77 - 0.90)	0.74 (0.65 - 0.83)	
**2021 COVID-19 hospitalised cohort**			
White	0.99 (0.95 - 1.03)	0.81 (0.76 - 0.87)	
Asian or Asian British	0.94 (0.83 - 1.07)	1.02 (0.86 - 1.22)	
Black	1.03 (0.85 - 1.25)	0.90 (0.69 - 1.17)	
Mixed	0.83 (0.60 - 1.14)	0.55 (0.35 - 0.84)	
Other	0.70 (0.52 - 0.96)	0.73 (0.47 - 1.12)	
Not stated	0.88 (0.82 - 0.94)	0.76 (0.69 - 0.85)	
**2022 COVID-19 hospitalised cohort**			
White	0.65 (0.63 - 0.68)	0.62 (0.58 - 0.65)	
Asian or Asian British	0.75 (0.65 - 0.86)	0.75 (0.63 - 0.90)	
Black	0.72 (0.58 - 0.90)	0.68 (0.52 - 0.90)	
Mixed	0.62 (0.44 - 0.88)	0.51 (0.32 - 0.80)	
Other	0.58 (0.42 - 0.80)	0.61 (0.41 - 0.91)	
Not stated	0.61 (0.57 - 0.66)	0.60 (0.54 - 0.66)	

*Fully adjusted models include age, sex, IMD quintile, region, ethnicity, obesity, smoking status, hypertension, diabetes, chronic respiratory disease, asthma, chronic cardiac disease, lung cancer, haematological cancer, other cancer, chronic liver disease, other neurological disease, organ transplant, asplenia, HIV, permanent immunodeficiency, and rheumatoid arthritis/systemic lupus erythematosus/psoriasis.

Supplementary Table 10. Crude and adjusted hazard ratio of first sick note for all comparisons, stratified by IMD quintile

	COVID-19 cohort vs 2019 general population		COVID-19 cohort vs contemporary general population	
	Crude HR (95%CI)	Fully adjusted HR (95%CI)	Crude HR (95%CI)	Fully adjusted HR (95%CI)
**2020 COVID-19 cohort**				
1 (most deprived)	2.64 (2.59 - 2.70)	2.70 (2.64 - 2.76)	3.44 (3.36 - 3.51)	3.41 (3.33 - 3.48)
2	3.05 (2.98 - 3.13)	3.06 (2.98 - 3.14)	3.96 (3.86 - 4.06)	3.91 (3.81 - 4.02)
3	3.45 (3.36 - 3.54)	3.43 (3.33 - 3.52)	4.41 (4.29 - 4.53)	4.36 (4.24 - 4.49)
4	3.71 (3.61 - 3.82)	3.71 (3.61 - 3.82)	4.72 (4.59 - 4.86)	4.71 (4.57 - 4.86)
5 (least deprived)	3.86 (3.74 - 3.99)	3.93 (3.80 - 4.06)	5.02 (4.86 - 5.18)	5.15 (4.98 - 5.32)
**2021 COVID-19 cohort**				
1 (most deprived)	1.84 (1.81 - 1.86)	1.86 (1.84 - 1.89)	1.77 (1.75 - 1.79)	1.77 (1.74 - 1.79)
2	1.93 (1.90 - 1.96)	1.98 (1.94 - 2.01)	1.93 (1.90 - 1.96)	1.95 (1.92 - 1.98)
3	2.04 (2.00 - 2.07)	2.10 (2.06 - 2.14)	2.06 (2.02 - 2.09)	2.09 (2.06 - 2.13)
4	2.09 (2.05 - 2.13)	2.15 (2.10 - 2.19)	2.09 (2.05 - 2.12)	2.15 (2.11 - 2.19)
5 (least deprived)	2.18 (2.14 - 2.23)	2.25 (2.20 - 2.30)	2.21 (2.17 - 2.26)	2.30 (2.26 - 2.35)
**2022 COVID-19 cohort**				
1 (most deprived)	1.68 (1.66 - 1.70)	1.59 (1.57 - 1.62)	1.58 (1.55 - 1.60)	1.55 (1.53 - 1.57)
2	1.64 (1.62 - 1.67)	1.57 (1.54 - 1.59)	1.60 (1.58 - 1.62)	1.59 (1.56 - 1.61)
3	1.62 (1.60 - 1.65)	1.55 (1.52 - 1.58)	1.58 (1.56 - 1.61)	1.57 (1.55 - 1.59)
4	1.64 (1.62 - 1.67)	1.53 (1.49 - 1.56)	1.61 (1.59 - 1.64)	1.60 (1.58 - 1.63)
5 (least deprived)	1.62 (1.59 - 1.65)	1.50 (1.47 - 1.54)	1.56 (1.54 - 1.59)	1.56 (1.53 - 1.58)

	COVID-19 hospitalised cohort vs 2019 pneumonia hospitalised cohort	
	Crude HR (95%CI)	Fully adjusted HR (95%CI)
**2020 COVID-19 hospitalised cohort**		
1 (most deprived)	0.96 (0.89 - 1.03)	0.76 (0.68 - 0.84)
2	0.9 (0.83 - 0.97)	0.73 (0.65 - 0.82)
3	0.93 (0.85 - 1.01)	0.83 (0.73 - 0.94)
4	0.88 (0.81 - 0.96)	0.84 (0.73 - 0.96)

5 (least deprived)	0.78 (0.70 - 0.86)	0.71 (0.60 - 0.83)	
**2021 COVID-19 hospitalised cohort**			
1 (most deprived)	1.02 (0.97 - 1.09)	0.85 (0.78 - 0.93)	
2	0.96 (0.90 - 1.03)	0.80 (0.72 - 0.89)	
3	0.96 (0.90 - 1.04)	0.89 (0.79 - 0.99)	
4	0.88 (0.81 - 0.96)	0.79 (0.70 - 0.90)	
5 (least deprived)	0.82 (0.75 - 0.90)	0.69 (0.60 - 0.79)	
**2022 COVID-19 hospitalised cohort**			
1 (most deprived)	0.71 (0.67 - 0.76)	0.66 (0.60 - 0.72)	
2	0.65 (0.61 - 0.70)	0.63 (0.57 - 0.70)	
3	0.62 (0.57 - 0.66)	0.63 (0.57 - 0.70)	
4	0.60 (0.56 - 0.65)	0.57 (0.51 - 0.64)	
5 (least deprived)	0.60 (0.55 - 0.65)	0.59 (0.52 - 0.67)	

*Fully adjusted models include age, sex, IMD quintile, region, ethnicity, obesity, smoking status, hypertension, diabetes, chronic respiratory disease, asthma, chronic cardiac disease, lung cancer, haematological cancer, other cancer, chronic liver disease, other neurological disease, organ transplant, asplenia, HIV, permanent immunodeficiency, and rheumatoid arthritis/systemic lupus erythematosus/psoriasis.

Supplementary Table 11. Crude and adjusted hazard ratio of first sick note for all comparisons, stratified by region

	COVID-19 cohort vs 2019 general population		COVID-19 cohort vs contemporary general population	
	**Crude HR (95%CI)**	**Fully adjusted HR (95%CI)**	**Crude HR (95%CI)**	**Fully adjusted HR (95%CI)**
**2020 COVID-19 cohort**				
East Midlands	3.13 (3.05 - 3.22)	3.12 (3.04 - 3.21)	4.05 (3.94 - 4.16)	3.98 (3.87 - 4.09)
East	3.34 (3.24 - 3.45)	3.17 (3.07 - 3.27)	4.56 (4.42 - 4.71)	4.28 (4.14 - 4.43)
London	3.68 (3.45 - 3.92)	3.24 (3.03 - 3.47)	5.22 (4.89 - 5.58)	4.57 (4.26 - 4.89)
North East	3.12 (3.00 - 3.24)	3.15 (3.03 - 3.28)	3.86 (3.71 - 4.02)	3.86 (3.71 - 4.02)
North West	3.20 (3.11 - 3.30)	3.24 (3.14 - 3.33)	3.94 (3.82 - 4.06)	3.92 (3.80 - 4.04)
South East	3.28 (3.06 - 3.52)	3.14 (2.92 - 3.37)	4.34 (4.04 - 4.65)	4.15 (3.86 - 4.46)
South West	3.44 (3.29 - 3.61)	3.36 (3.21 - 3.52)	4.55 (4.35 - 4.77)	4.40 (4.19 - 4.61)
West Midlands	3.29 (3.14 - 3.46)	3.32 (3.16 - 3.49)	4.37 (4.16 - 4.59)	4.34 (4.13 - 4.57)
Yorkshire & The Humber	3.15 (3.07 - 3.22)	3.16 (3.08 - 3.24)	4.02 (3.92 - 4.12)	3.97 (3.88 - 4.07)
**2021 COVID-19 cohort**				
East Midlands	2.05 (2.02 - 2.09)	2.06 (2.02 - 2.10)	1.95 (1.92 - 1.98)	1.99 (1.96 - 2.02)
East	2.01 (1.97 - 2.05)	1.97 (1.93 - 2.01)	2.07 (2.03 - 2.11)	2.07 (2.04 - 2.11)
London	2.63 (2.53 - 2.74)	2.62 (2.50 - 2.74)	2.69 (2.59 - 2.79)	2.60 (2.50 - 2.70)
North East	1.96 (1.91 - 2.01)	1.97 (1.91 - 2.03)	1.85 (1.80 - 1.90)	1.89 (1.84 - 1.94)
North West	1.85 (1.81 - 1.88)	1.87 (1.83 - 1.91)	1.73 (1.69 - 1.76)	1.76 (1.72 - 1.79)
South East	2.02 (1.94 - 2.10)	1.98 (1.90 - 2.07)	2.02 (1.95 - 2.09)	2.05 (1.98 - 2.12)
South West	2.03 (1.98 - 2.09)	1.99 (1.93 - 2.04)	2.02 (1.97 - 2.06)	2.06 (2.02 - 2.11)
West Midlands	2.10 (2.03 - 2.17)	2.18 (2.1 - 2.26)	2.04 (1.98 - 2.11)	2.08 (2.01 - 2.14)
Yorkshire & The Humber	2.03 (1.99 - 2.06)	2.04 (2.00 - 2.07)	1.92 (1.89 - 1.95)	1.95 (1.92 - 1.99)
**2022 COVID-19 cohort**				
East Midlands	1.71 (1.68 - 1.73)	1.58 (1.55 - 1.61)	1.55 (1.53 - 1.58)	1.57 (1.55 - 1.60)
East	1.61 (1.58 - 1.64)	1.50 (1.47 - 1.53)	1.55 (1.53 - 1.58)	1.57 (1.55 - 1.59)
London	1.91 (1.84 - 1.98)	1.96 (1.88 - 2.05)	1.79 (1.74 - 1.86)	1.90 (1.83 - 1.96)
North East	1.68 (1.63 - 1.73)	1.56 (1.51 - 1.61)	1.50 (1.46 - 1.54)	1.53 (1.49 - 1.57)
North West	1.63 (1.60 - 1.66)	1.49 (1.46 - 1.53)	1.51 (1.48 - 1.54)	1.51 (1.48 - 1.54)
South East	1.67 (1.61 - 1.73)	1.53 (1.47 - 1.59)	1.54 (1.50 - 1.58)	1.54 (1.50 - 1.58)

South West	1.66 (1.62 - 1.69)	1.47 (1.44 - 1.51)	1.54 (1.52 - 1.57)	1.54 (1.52 - 1.57)
West Midlands	1.80 (1.75 - 1.86)	1.74 (1.68 - 1.81)	1.62 (1.57 - 1.67)	1.65 (1.60 - 1.71)
Yorkshire & The Humber	1.65 (1.63 - 1.68)	1.56 (1.53 - 1.59)	1.53 (1.51 - 1.56)	1.58 (1.55 - 1.61)

	**COVID-19 hospitalised cohort vs 2019 pneumonia hospitalised cohort**	
	**Crude HR (95%CI)**	**Fully adjusted HR (95%CI)**
**2020 COVID-19 hospitalised cohort**		
East Midlands	0.83 (0.77 - 0.91)	0.69 (0.61 - 0.78)
East	0.78 (0.67 - 0.91)	0.74 (0.65 - 0.84)
London	0.94 (0.82 - 1.09)	0.65 (0.51 - 0.83)
North East	1.06 (0.95 - 1.18)	0.81 (0.65 - 1.02)
North West	0.75 (0.63 - 0.90)	0.89 (0.76 - 1.04)
South East	1.10 (0.98 - 1.23)	0.58 (0.45 - 0.76)
South West	0.88 (0.75 - 1.02)	1.08 (0.91 - 1.28)
West Midlands	0.96 (0.88 - 1.05)	0.74 (0.59 - 0.93)
Yorkshire & The Humber	0.83 (0.77 - 0.91)	0.78 (0.69 - 0.90)
**2021 COVID-19 hospitalised cohort**		
East Midlands	0.91 (0.85 - 0.98)	0.78 (0.69 - 0.87)
East	0.92 (0.85 - 0.99)	0.79 (0.70 - 0.88)
London	0.86 (0.74 - 1.00)	0.80 (0.65 - 1.00)
North East	0.94 (0.83 - 1.08)	0.83 (0.68 - 1.03)
North West	0.98 (0.89 - 1.09)	0.81 (0.70 - 0.95)
South East	0.90 (0.78 - 1.04)	0.79 (0.63 - 0.99)
South West	0.99 (0.90 - 1.09)	0.82 (0.71 - 0.95)
West Midlands	0.96 (0.84 - 1.11)	0.92 (0.74 - 1.15)
Yorkshire & The Humber	0.98 (0.91 - 1.06)	0.86 (0.77 - 0.97)
**2022 COVID-19 hospitalised cohort**		
East Midlands	0.68 (0.63 - 0.73)	0.68 (0.61 - 0.75)
East	0.60 (0.56 - 0.65)	0.60 (0.54 - 0.66)
London	0.61 (0.52 - 0.71)	0.72 (0.58 - 0.90)
North East	0.59 (0.52 - 0.68)	0.58 (0.48 - 0.70)

North West	0.69 (0.63 - 0.77)	0.61 (0.53 - 0.71)	
South East	0.58 (0.51 - 0.66)	0.56 (0.46 - 0.67)	
South West	0.63 (0.58 - 0.69)	0.58 (0.51 - 0.67)	
West Midlands	0.74 (0.64 - 0.86)	0.72 (0.58 - 0.88)	
Yorkshire & The Humber	0.69 (0.64 - 0.75)	0.61 (0.54 - 0.68)	

*Fully adjusted models include age, sex, IMD quintile, region, ethnicity, obesity, smoking status, hypertension, diabetes, chronic respiratory disease, asthma, chronic cardiac disease, lung cancer, haematological cancer, other cancer, chronic liver disease, other neurological disease, organ transplant, asplenia, HIV, permanent immunodeficiency, and rheumatoid arthritis/systemic lupus erythematosus/psoriasis.

Supplementary Figure 3. Adjusted hazard ratio of first sick note comparing hospitalised COVID-19 cohorts to people hospitalised with pneumonia in 2019, stratified by demographic categories and year

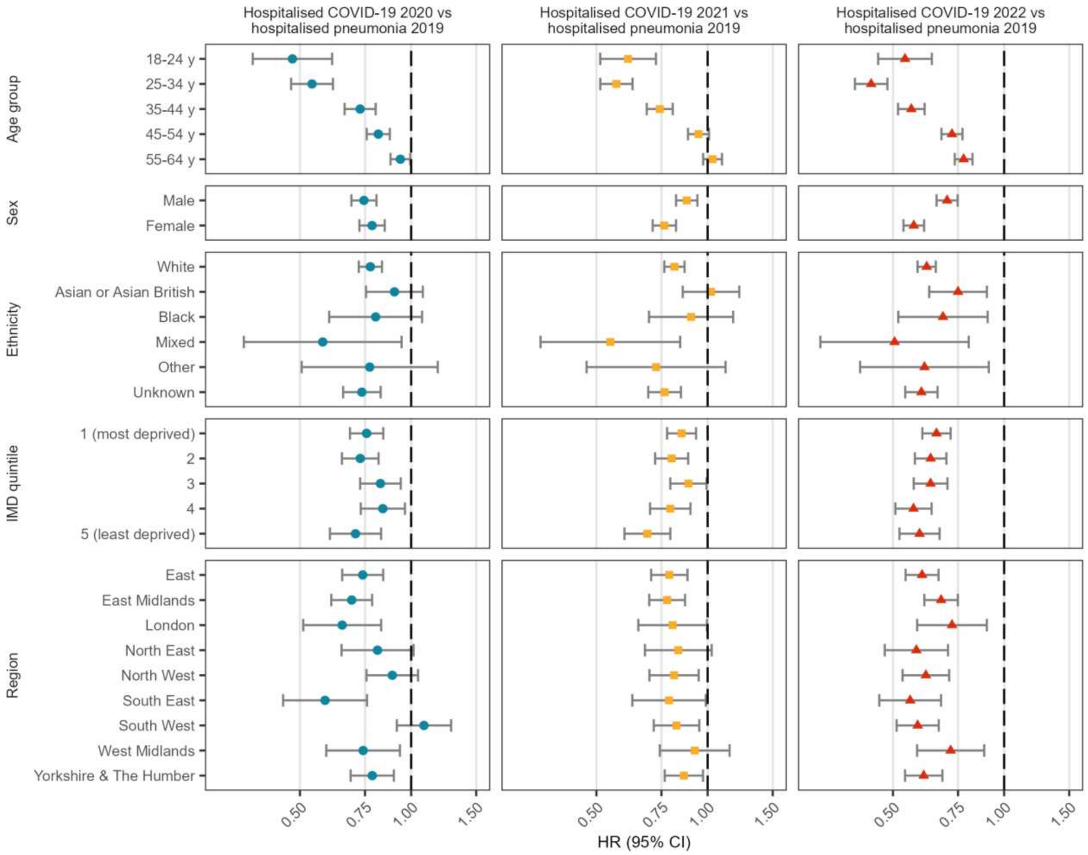

*All models are adjusted for age, sex, ethnicity, IMD quintile and region, excluding the stratification variable. Models are additionally adjusted for obesity, smoking status, hypertension, diabetes, chronic respiratory disease, asthma, chronic cardiac disease, lung cancer, haematological cancer, other cancer, chronic liver disease, other neurological disease, organ transplant, asplenia, HIV, permanent immunodeficiency, and rheumatoid arthritis/systemic lupus erythematosus/psoriasis

Information governance

NHS England is the data controller of the NHS England OpenSAFELY COVID-19 Service; TPP is the data processor; all study authors using OpenSAFELY have the approval of NHS England.(1) This implementation of OpenSAFELY is hosted within the TPP environment which is accredited to the ISO 27001 information security standard and is NHS IG Toolkit compliant.(2)

Patient data has been pseudonymised for analysis and linkage using industry standard cryptographic hashing techniques; all pseudonymised datasets transmitted for linkage onto OpenSAFELY are encrypted; access to the NHS England OpenSAFELY COVID-19 service is via a virtual private network (VPN) connection; the researchers hold contracts with NHS England and only access the platform to initiate database queries and statistical models; all database activity is logged; only aggregate statistical outputs leave the platform environment following best practice for anonymisation of results such as statistical disclosure control for low cell counts.(3)

The service adheres to the obligations of the UK General Data Protection Regulation (UK GDPR) and the Data Protection Act 2018. The service previously operated under notices initially issued in February 2020 by the the Secretary of State under Regulation 3(4) of the Health Service (Control of Patient Information) Regulations 2002 (COPI Regulations), which required organisations to process confidential patient information for COVID-19 purposes; this set aside the requirement for patient consent.(4) As of 1 July 2023, the Secretary of State has requested that NHS England continue to operate the Service under the COVID-19 Directions 2020.(5) In some cases of data sharing, the common law duty of confidence is met using, for example, patient consent or support from the Health Research Authority Confidentiality Advisory Group.(6)

Taken together, these provide the legal bases to link patient datasets using the service. GP practices, which provide access to the primary care data, are required to share relevant health information to support the public health response to the pandemic, and have been informed of how the service operates.

(1) NHS Digital. The NHS England OpenSAFELY COVID-19 service - privacy notice [Internet]. 2023 [cited 2023 Jul 5]. Available from: https://digital.nhs.uk/coronavirus/coronavirus-covid-19-response-information-governance-hub/the-nhs-england-opensafely-covid-19-service-privacy-notice

(2) NHS Digital. NHS Digital. 2023 [cited 2023 Jul 5]. Data Security and Protection Toolkit. Available from: https://digital.nhs.uk/data-and-information/looking-after-information/data-security-and-information-governance/data-security-and-protection-toolkit

(3) NHS Digital [Internet]. [cited 2023 Mar 6]. ISB1523: Anonymisation Standard for Publishing Health and Social Care Data. Available from: https://digital.nhs.uk/data-and-information/information-standards/information-standards-and-data-collections-including-extractions/publications-and-notifications/standards-and-collections/isb1523-anonymisation-standard-for-publishing-health-and-social-care-data

(4) UK Department of Health and Social Care. GOV.UK. 2022 [cited 2023 Jul 5]. [Withdrawn] Coronavirus (COVID-19): notice under regulation 3(4) of the Health Service (Control of Patient Information) Regulations 2002 – general. Available from: https://www.gov.uk/government/publications/coronavirus-covid-19-

notification-of-data-controllers-to-share-information/coronavirus-covid-19-notice-under-regulation-34-of-the-health-service-control-of-patient-information-regulations-2002-general--2

(5) NHS Digital. NHS Digital. 2022 [cited 2023 Jul 5]. Secretary of State for Health and Social Care: COVID-19 Public Health Directions 2020. Available from: https://digital.nhs.uk/about-nhs-digital/corporate-information-and-documents/directions-and-data-provision-notices/secretary-of-state-directions/covid-19-public-health-directions-2020

(6) NHS Health Research Authority. Health Research Authority. [cited 2023 Jul 5]. Confidentiality Advisory Group. Available from: https://www.hra.nhs.uk/about-us/committees-and-services/confidentiality-advisory-group/
