# Supplementary material for: Changes in sick notes associated with COVID-19 from 2020 to 2022: a cohort study in 24 million primary care patients in OpenSAFELY-TPP
Source: BMJ Open. 2024 Jul 3;14(7):e080600. doi: 10.1136/bmjopen-2023-080600 (PMC11227761; doi:10.1136/bmjopen-2023-080600)
Supplement: Author's manuscript [file bmjopen-2023-080600.draft_revisions.pdf]

BMJ Open is committed to open peer review. As part of this commitment we make the peer review history of every article we publish publicly available.

When an article is published we post the peer reviewers' comments and the authors' responses online. We also post the versions of the paper that were used during peer review. These are the versions that the peer review comments apply to.

The versions of the paper that follow are the versions that were submitted during the peer review process. They are not the versions of record or the final published versions. They should not be cited or distributed as the published version of this manuscript.

BMJ Open is an open access journal and the full, final, typeset and author-corrected version of record of the manuscript is available on our site with no access controls, subscription charges or pay-per-view fees (http://bmjopen.bmj.com).

If you have any questions on BMJ Open's open peer review process please email info.bmjopen@bmj.com

BMJ Open

Changes in fit notes associated with COVID-19 from 2020 to 2022: A cohort study in 24 million primary care patients in OpenSAFELY-TPP

Journal:	*BMJ Open*
Manuscript ID	bmjopen-2023-080600
Article Type:	Original research
Date Submitted by the Author:	05-Oct-2023
Complete List of Authors:	Schaffer, Andrea; University of Oxford, Nuffield Department of Primary Care Health Sciences<br>Park, Robin Y; University of Oxford, Nuffield Department of Primary Care Health Sciences<br>Tazare, John; London School of Hygiene & Tropical Medicine<br>Bhaskaran, Krishnan; LSHTM, NCDE<br>MacKenna, Brian; University of Oxford, Nuffield Department of Primary Care Health Sciences<br>Denaxas, S; UCL, Institute of Health Informatics; NIHR University College London Hospitals Biomedical Research Centre<br>Dillingham, Iain; University of Oxford, Nuffield Department of Primary Care Health Sciences<br>Bacon, Seb; University of Oxford, Nuffield Department of Primary Care Health Sciences<br>Mehrkar, Amir; University of Oxford Nuffield Department of Primary Care Health Sciences<br>Bates, Christopher; TPP<br>Goldacre, Ben; University of Oxford, Primary Care Health Sciences<br>Greaves, Felix; National Institute for Health and Care Excellence; Imperial College London Department of Primary Care and Public Health<br>Macleod, John; NIHR Applied Research Collaboration West<br>Consortium, The OpenSAFELY ; Oxford University<br>Collaborative, National Core Studies; University of Oxford Nuffield Department of Primary Care Health Sciences, Bennett Institute for Applied Data Science<br>Tomlinson, Laurie; London School of Hygiene and Tropical Medicine, Epidemiology and Population Health<br>Walker, Alex; University of Oxford, Nuffield Department of Primary Care Health Sciences
Keywords:	COVID-19, EPIDEMIOLOGY, PUBLIC HEALTH, Primary Care < Primary Health Care

SCHOLARONE™
Manuscripts

I, the Submitting Author has the right to grant and does grant on behalf of all authors of the Work (as defined in the below author licence), an exclusive licence and/or a non-exclusive licence for contributions from authors who are: i) UK Crown employees; ii) where BMJ has agreed a CC-BY licence shall apply, and/or iii) in accordance with the terms applicable for US Federal Government officers or employees acting as part of their official duties; on a worldwide, perpetual, irrevocable, royalty-free basis to BMJ Publishing Group Ltd ("BMJ") its licensees and where the relevant Journal is co-owned by BMJ to the co-owners of the Journal, to publish the Work in this journal and any other BMJ products and to exploit all rights, as set out in our licence.

The Submitting Author accepts and understands that any supply made under these terms is made by BMJ to the Submitting Author unless you are acting as an employee on behalf of your employer or a postgraduate student of an affiliated institution which is paying any applicable article publishing charge ("APC") for Open Access articles. Where the Submitting Author wishes to make the Work available on an Open Access basis (and intends to pay the relevant APC), the terms of reuse of such Open Access shall be governed by a Creative Commons licence – details of these licences and which Creative Commons licence will apply to this Work are set out in our licence referred to above.

Other than as permitted in any relevant BMJ Author's Self Archiving Policies, I confirm this Work has not been accepted for publication elsewhere, is not being considered for publication elsewhere and does not duplicate material already published. I confirm all authors consent to publication of this Work and authorise the granting of this licence.

For peer review only

Changes in fit notes associated with COVID-19 from 2020 to 2022: A cohort study in 24 million primary care patients in OpenSAFELY-TPP

Andrea L Schaffer[1*], Robin Y Park[1], John Tazare[2], Krishnan Bhaskaran[2], Brian MacKenna[1], Spiros Denaxas[3,4,5], Iain Dillingham[1], Sebastian CJ Bacon[1], Amir Mehrkar[1], Chris Bates[6], Ben Goldacre[1], Felix Greaves[7,8], John Macleod[9], The OpenSAFELY Collaborative^, National Core Studies Collaborative^, Laurie A Tomlinson[2], Alex J Walker[1],

[1] Bennett Institute for Applied Data Science, Nuffield Department of Primary Care Health Sciences, University of Oxford, Oxford, UK

[2] London School of Hygiene and Tropical Medicine, London, UK

[3] Institute of Health Informatics, University College London, London, UK

[4] University College London Hospitals Biomedical Research Centre, London, UK

[5] BHF Data Science Center, Health Data Research UK, London, UK

[6] TPP, TPP House, Leeds, UK

[7] Department of Primary Care and Public Health, Imperial College London, London, UK

[8] National Institute for Health and Care Excellence, London, UK

[9] NIHR Applied Research Collaboration (ARC) West, Bristol, UK

*Corresponding author:

Dr Andrea Schaffer andrea.schaffer@phc.ox.ac.uk

Word count: 3122

^Members of the collaboratives are listed in the Acknowledgements

For peer review only - http://bmjopen.bmj.com/site/about/guidelines.xhtml

Abstract

Objectives: Long-term sickness absence from employment has negative economic consequences and can widen health inequalities. We quantified the fit note ("sick note") rate in people with and without COVID-19 (2020, 2021, 2022), as an indication of the public health and economic burden for people recovering from COVID-19.

Design: Cohort study.

Setting: With NHS England approval, we used routine clinical data (primary care, hospital and COVID-19 testing records) within the OpenSAFELY-TPP database

Participants: People 18-64 years with a recorded positive test or diagnosis of COVID-19 in 2020 (n=365,421), 2021 (n=1,206,555) or 2022 (n=1,321,313); general population matched on age, sex and region in 2019 (n=3,140,326), 2020 (n=3,439,534), 2021 (n=4,571,469), and 2022 (n=4,818,870); people hospitalised with pneumonia in 2019 (n=29,673).

Outcome measure: Receipt of a fit note in primary care.

Results: Among people with a positive SARS-CoV-2 test or COVID-19 diagnosis, the fit note rate was 4.88 per 100 person-months (95%CI 4.83-4.93) in 2020, 2.66 (95%CI 2.64-2.67) in 2021, and 1.73 (95%CI 1.72-1.73) in 2022. Compared with the age, sex and region matched general population, the adjusted hazard ratio (HR) for receipt of a fit note over the entire follow-up period was 4.07 (95%CI 4.02-4.12) in 2020 decreasing to 1.57 (95%CI 1.56-1.58) in 2022. The HR was highest in the first 30 days post-diagnosis in all years. Among the subset of people hospitalised with COVID-19, after adjustment the fit note rate was lower than in people hospitalised with pneumonia.

Conclusions: Given the under-recording of post-acute COVID-19-related symptoms, these findings contribute a valuable perspective on the long-term effects of COVID-19. We identified a considerable increase among people with COVID-19, even in an era when most people are vaccinated. Most fit notes are associated with the acute phase of the disease, but the increased risk several months post-diagnosis provides further evidence of the long-term impact.

Keywords

sars-cov-2, covid-19, long covid, fit notes, sick notes, sick leave

For peer review only

Strengths and limitations of this study

- Our data come from a large, representative sample of 40% of primary care patients in England.
- To better understand fit note patterns, we identified COVID-19 cohorts from three different pandemic time periods, with both contemporary and historical comparators, to account for changes in preventive measures and disruptions to working patterns that would have impacted on receipt of fit notes over time.
- We relied on three different methods for identifying COVID-19 cases (positive PCR or LFT test, primary care diagnosis, hospitalisation), but there is still bias in who gets tested or seeks care for COVID-19 which will have changed as the pandemic evolved. It is unclear how this would correlate with the likelihood of requesting or requiring a fit note.
- We could not identify whether people were participating in the workforce and included everyone of working age. It is therefore likely that the true absolute fit note rate is higher than estimated.

For peer review only

For peer review only - http://bmjopen.bmj.com/site/about/guidelines.xhtml

Background

In primary care, a doctor may issue a fit note (also called “sick note”) after the first seven days of sickness absence if the doctor assesses that the patient’s health affects their fitness for work. In 2021-22, over 11 million fit notes were issued in England.(1) Long-term sickness absence from employment has negative consequences for the economy as well as individuals and can lead to widened health inequalities, financial insecurity, and reduced social participation.(2) Improving the health and productivity of the population and reducing welfare benefit claims are important policy objectives.(3,4)

The Office of National Statistics (ONS) estimated that 82% of the English population had been infected with SARS-CoV-2 by November 2022.(5,6) Additionally, an estimated 1.9 million people self-reported as experiencing long COVID symptoms in the UK in the four weeks prior to March 2023, with two-thirds experiencing symptoms for at least one year.(7) The prevalence of long-term symptoms was greatest in people aged 35-69 years, women, and people living in more deprived areas.(7) Previous UK research has also shown that receipt of fit notes varies by sociodemographics, with women and people in manual and service occupations more likely to be issued a fit note.(4) The most common reasons for receiving a fit note prior to the COVID-19 pandemic included mental health and musculoskeletal disorders.(1,8) To date, there has been limited research on fit notes issued to patients recovering from COVID-19 in England.

Given the risk of long-term symptoms following COVID-19 (“long COVID”), it is of public health interest to quantify the impact of SARS-CoV-2 infection and recovery on the workforce. However, long COVID is heterogeneous and difficult to define.(9,10) Furthermore, coding of long COVID in primary care is very low,(11) and cannot be relied on to identify people suffering from persistent symptoms. Therefore, our objectives were to: 1) describe the demographic and clinical characteristics of people given a fit note following a documented SARS-CoV-2 infection or COVID-19 diagnosis; 2) determine how the fit note rate varies over time post-diagnosis; and 3) quantify the difference in fit note rate in people with SARS-CoV-2 infection or COVID-19 diagnosis compared with the general population and people hospitalised with pneumonia.

Methods

Study design

We conducted an observational cohort study using general practice primary care electronic health record (EHR) data from primary care practices in England.

Data source and data sharing

We used primary care data from approximately 40% of the English population currently registered with GP surgeries using TPP SystmOne software. All data were linked, stored and analysed securely using the OpenSAFELY platform, https://www.opensafely.org/, as part of the NHS England OpenSAFELY COVID-19 service. Data include pseudonymised data

For peer review only - http://bmjopen.bmj.com/site/about/guidelines.xhtml

such as coded diagnoses, medications and physiological parameters. No free text data are included. All code is shared openly for review and re-use under MIT open license (https://github.com/opensafely/long-covid-sick-notes). Similarly, pseudonymised datasets including ONS registered deaths, hospital episode statistics (HES), and Second Generation Surveillance System (SGSS) COVID-19 test results are securely provided to TPP and linked to primary care data. Detailed pseudonymised patient data are potentially re-identifiable and not shared.

Study population

We included adults 18-64 years registered with one GP for at least one year prior to their index date with information on age, sex, index of multiple deprivation (IMD) and the Sustainability and Transformation Partnership region (STP, an NHS administrative region). This age range was selected to represent people most likely to be in the workforce. From this source population, we identified three cohorts with recorded SARS-CoV-2 infection between 1 February and 30 November in each of 2020, 2021 and 2022 (hereafter referred to as the "COVID-19 cohorts"). They were identified through three routes: having a recorded positive test for SARS-CoV-2 based on SGSS data, which captures both polymerase chain reaction (PCR) and lateral flow tests (LFT); having a probable diagnostic code for COVID-19 in primary care records; or being hospitalised with a primary or secondary diagnosis for COVID-19 (ICD-10 codes U07.1 or U07.2) identified from HES. The index date was the earliest of these events. As comparators, we identified contemporary (2020, 2021, 2022) and one historical (2019) general population cohort who were frequency-matched on age, sex and STP to the COVID-19 cohorts. The 2019 general population cohort was matched with the 2020 COVID-19 cohort. For the comparator cohorts, the index date was randomly assigned and randomly distributed over the study period.

We also performed a secondary analysis among the subset of people hospitalised with COVID-19. Here, the index date was the date of admission. We compared these individuals with people hospitalised with pneumonia between 1 February 2019 and 30 November 2019. The pneumonia cohort was identified using the following ICD-10 codes identified in any diagnosis position: B01.2, B05.2, B20.6, B25.0, J10-J18, J85.1, U04. No contemporary pneumonia comparator cohort was included due to potential for misclassification with COVID-19. People could be included in multiple cohorts if they met the inclusion criteria.

Study measures

Primary outcome

We identified the first recorded fit note over patient follow-up. Fit notes were identified using Clinical Terms Version 3 (CTV3) codes. People who were required to isolate beyond 7 days due to having or living with someone with symptoms of COVID-19 would be issued an isolation note, rather than a fit note. Isolation notes did not require contact with a GP, and are not counted. Follow-up was censored at the end of the study period (30 November), death, or deregistration from their GP practice. The comparator cohorts were also censored if they had a recorded positive SARS-CoV-2 test or COVID-19 diagnosis.

For peer review only - http://bmjopen.bmj.com/site/about/guidelines.xhtml

Demographics and clinical characteristics

We characterised the demographic and clinical characteristics of patients who received a fit note. Measured demographic variables included age (18-24, 25-34, 35-44, 45-54, 55-64 years), sex (male, female), GP practice region (East Midlands, East, London, North East, North West, South East, South West, West Midlands, Yorkshire and The Humber), ethnicity (White, Asian and British Asian, Black, Other, Unknown), and Indices of Multiple Deprivation (IMD) quintiles.

Clinical variables included indicators for pre-existing conditions that may impact the severity of COVID-19, specifically: asthma, cancer (haematological, lung, other), chronic cardiac disease, chronic liver disease, chronic respiratory disease (excluding asthma), diabetes, asplenia, human immunodeficiency virus (HIV) infection, hypertension, obesity, organ transplant, neurological conditions, other permanent immunodeficiency (excluding HIV), smoking status (current, former or never), and autoimmune conditions (rheumatoid arthritis, systemic lupus erythematosus (SLE), psoriasis). Obesity and smoking status were identified using the most recent recorded information, while the remainder were identified at any time prior to the index date.

Statistical methods

Rate of first fit note

For each of the eleven cohorts (three COVID-19 cohorts [2020, 2021, 2022], four matched general population cohorts [2019, 2020, 2021, 2022], three hospitalised COVID-19 cohorts [2020, 2021, 2022] and one hospitalised pneumonia cohort [2019]), we reported the first fit note rate per 100 person months overall and stratified by demographics (age, sex, ethnicity, region, IMD quintile). To prevent disclosure, all counts in this manuscript are rounded to the nearest 7.

Cox regression

We used Cox regression models to estimate hazard ratios (HRs) and 95%Confidence intervals (CIs) comparing the first fit note rate between the COVID-19 cohorts and the comparator cohorts. All of the COVID-19 cohorts were compared with their contemporary general population cohort as well as the historical 2019 general population cohort. The hospitalised COVID-19 cohorts were compared with the hospitalised pneumonia cohort (2019) only.

We investigated crude univariable and two covariate-adjusted models: (1) adjusted for age (cubic splines with 4 knots) and sex; (2) adjusted for demographics and all clinical characteristics described above. While the same people can contribute person-time to multiple exposure groups, these periods are non-overlapping, so we applied robust standard errors.

To determine if the relative differences in fit note occurrence changed over time, we re-estimated crude and adjusted HRs and 95%CIs censoring follow-up at 30, 90, and 150 days, and examined whether HRs changed according to the duration of follow-up. We chose to

For peer review only - http://bmjopen.bmj.com/site/about/guidelines.xhtml

use this approach instead of estimating period-specific HRs in order to avoid selection bias due to depletion of susceptibles.(12) Similarly, to explore the impact within demographic categories, we estimated crude and adjusted HRs and 95%CIs stratified by age group, sex, ethnicity, IMD quintile, and region.

Software and reproducibility

Data management was performed using Python 3.8, with analyses carried out using Stata 16.1, R 4.3.0, and Python. Code for data management and analysis as well as all codelists used in this study are available online: https://github.com/opensafely/long-covid-sick-notes.

Patient and public involvement

We have a publicly available website https://opensafely.org/ through which we invite any patient or member of the public to contact us regarding this study or the broader OpenSAFELY project.

Results

Study population description

We identified 365,421 people with a recorded positive SARS-CoV-2 test or COVID-19 diagnosis in 2020, of which 22,015 (6.0%) were hospitalised; 1,206,555 people in 2021 (30,205 [2.5%] hospitalised); and 1,321,313 people in 2022 (34,692 [2.6%] hospitalised) (**Table 1**). The matched general population cohorts included 3.1 million people in 2019, 3.4 million in 2020, 4.6 million in 2021, and 4.8 million in 2022. For comparison with COVID-19 patients who were hospitalised, we identified 29,673 patients hospitalised with pneumonia in 2019. The majority of people in the COVID-19 cohorts were identified via SGSS testing (88.8% in 2020, 97.8% in 2021 and 96.5% in 2022) (**Supplementary Figure 1**).

Compared with 2020 and 2021, the 2022 COVID-19 cohort was older and more likely to be female, White ethnicity, and in less deprived IMD quintiles. Hospitalised COVID-19 cohorts tended to be older, and were more likely to be male, non-White ethnicity, and in the most deprived IMD quintile compared with the overall COVID-19 cohorts (**Supplementary Table 1**). When compared with the pneumonia cohort, hospitalised COVID-19 patients were more likely to be obese,less likely to be current smokers, and had lower rates of many chronic health conditions.

Overall fit note rates

A total of 34,377 (9.4%), 102,949 (8.5%) and 152,859 (11.6%) of people in the COVID-19 cohorts were issued fit notes during follow-up in 2020, 2021 and 2022 (**Supplementary Table 2**). However, after taking into account differences in follow-up time (**Supplementary Figure 2**), the fit note rate among the COVID-19 cohorts decreased over time, from 4.88 per 100 person-months in 2020 (95%CI 4.83-4.93), to 2.66 (95%CI 2.64-2.67) in 2021 and 1.73 (95%CI 1.72-1.73) in 2022 (**Table 2**).

For peer review only - http://bmjopen.bmj.com/site/about/guidelines.xhtml

The fit note rate was higher in the hospitalised cohorts, and was 6.78 per 100 person-months (95%CI 6.59-6.98) in 2020, 7.19 (95%CI 7.03 - 7.36) in 2021 and 4.13 (95%CI 4.03-4.22) in 2022. In contrast, the fit note rate in the pneumonia cohort was 7.17 (95%CI 7.01-7.34) (**Supplementary Table 3**).

Fit note rate by demographics

Among people in the COVID-19 cohorts, the first fit note rate was higher in 2020 and lower in 2022 for all demographic groups except people 18-24 years (**Table 2**). Generally, the fit note rate was higher in people aged ≥45 years, women, people of Asian or Asian British ethnicity, and lower in people 18-24 years and living in London. The fit note rate increased with greater deprivation as defined by the IMD quintile. In most cases these patterns reflected those in the general population.

A slightly different pattern was observed for the hospitalised cohorts (**Supplementary Table 3**). People 45-54 years were most likely to receive a fit note, which was older than the pneumonia cohort. Among hospitalised patients the relationship between sex and receiving a fit note differed by year, with women more likely to receive a fit note in 2020 and men more likely in 2021 and 2022. A linear relationship between lesser deprivation and a higher fit note rate was seen for the pneumonia cohort, but not the hospitalised COVID-19 cohorts.

Cox regression by follow-up period

The overall fit note rate was higher in all COVID-19 cohorts than either their contemporary or 2019 cohorts. Most people in the COVID-19 cohorts who were issued a first fit note received it in the first 30 days post-index date (**Table 3**). The fully adjusted HR representing the average effect over the entire study period was highest comparing the 2020 COVID-19 cohort to the 2020 general population (4.07, 95%CI 4.02-4.12) and lowest comparing the 2022 COVID-19 cohort to the 2022 general population (1.57, 95%CI 1.56-1.58) (**Table 3**). For all comparisons of the COVID-19 population with the general population, the HR was greatest when we restricted the analysis to the first 30 days from the index date, with fully adjusted HRs in this early follow-up period ranging from 5.94 (95%CI 5.85-6.03) comparing the 2020 COVID-19 cohort to the 2020 general population to 2.37 (95%CI 2.34-2.39) comparing the 2022 COVID-19 cohort to the 2022 general population. With longer follow-up periods, the HR attenuated but remained high.

For the hospitalised cohorts, the overall fit note rate was lower in the COVID-19 cohorts than the pneumonia cohort with the highest rates in the first 30 days. The average HR over all follow-up time ranged from 0.62 (95%CI 0.59-0.65) for the 2022 COVID-19 cohort to 0.81 (95%CI 0.77-0.86) for the 2021 cohort (**Supplementary Table 4**). The HR was relatively stable regardless of follow-up period used.

Cox regression stratified by demographic categories

For all demographics groups, the HR was greatest in 2020 when compared with either a contemporary or 2019 historical comparator (**Figure 1, Supplementary Tables 5-9**). In

For peer review only - http://bmjopen.bmj.com/site/about/guidelines.xhtml

2020, large relative increases in fit note rate were observed for people ≥35 years, women, and people of Black or Other ethnicity. The HR was also higher in people in less deprived IMD quintiles; when comparing the 2020 COVID-19 cohort to the 2020 general population, the fully adjusted HR ranged from 3.41 (95%CI 3.33-3.48) in the most deprived quintile to 5.15 (95%CI 4.98-5.32) in the least deprived quintile.

These HRs were lower in 2021 compared with 2020, and more so in 2022. The one exception was people 18-24 years, where the HR compared with the general population was 1.47 (95%CI 1.43-1.51) in 2022, compared with 1.24 (95%CI 1.22-1.27) in 2021. Other patterns that differed in 2022 compared with other years was that no meaningful variation by IMD quintile was observed, and a higher HR was seen in men compared with women. The findings were similar comparing the COVID-19 cohorts with the 2019 comparator.

Among the hospitalised cohorts, the HRs comparing them with the pneumonia cohort were similar in 2020 and 2021, but lower in 2022 (**Supplementary Figure 3, Supplementary Tables 4-8**). A lower fit note rate in the COVID-19 cohorts compared with the pneumonia cohort was observed for most demographic subgroups.

For peer review only

For peer review only - http://bmjopen.bmj.com/site/about/guidelines.xhtml

Table 1. Demographic and clinical characteristics of all cohorts, 1 Feb - 30 Nov of each year. All counts rounded to nearest 7.

	2019	2020		2021		2022	
	General population*	COVID-19 cohort	General population^	COVID-19 cohort	General population^	COVID-19 cohort	General population^
	n (%)	n (%)	n (%)	n (%)	n (%)	n (%)	n (%)
**Total**	3,140,326 (100.0)	365,421 (100.0)	3,439,534 (100.0)	1,206,555 (100.0)	4,571,469 (100.0)	1,321,313 (100.0)	4,818,870 (100.0)
**Age**							
18-24 y	496,825 (15.8)	61,355 (16.8)	545,286 (15.9)	196,616 (16.3)	699,993 (15.3)	111,342 (8.4)	375,704 (7.8)
25-34 y	663,495 (21.1)	77,686 (21.3)	726,712 (21.1)	264,159 (21.9)	989,506 (21.6)	258,314 (19.5)	915,152 (19.0)
35-44 y	644,945 (20.5)	74,767 (20.5)	706,692 (20.5)	294,238 (24.4)	1,116,283 (24.4)	312,732 (23.7)	1,142,764 (23.7)
45-54 y	719,663 (22.9)	83,832 (22.9)	787,465 (22.9)	271,418 (22.5)	1,042,601 (22.8)	328,510 (24.9)	1,197,693 (24.9)
55-64 y	615,405 (19.6)	67,781 (18.5)	673,372 (19.6)	180,117 (14.9)	723,093 (15.8)	310,422 (23.5)	1,187,557 (24.6)
**Sex**							
Female	1,732,654 (55.2)	201,824 (55.2)	1,898,351 (55.2)	645,666 (53.5)	2,447,200 (53.5)	823,438 (62.3)	3,001,922 (62.3)
Male	1,407,672 (44.8)	163,597 (44.8)	1,541,183 (44.8)	560,889 (46.5)	2,124,269 (46.5)	497,875 (37.7)	1,816,948 (37.7)
**Ethnicity**							
White	1,914,458 (61.0)	213,185 (58.3)	2,109,478 (61.3)	788,249 (65.3)	2,866,892 (62.7)	967,225 (73.2)	3,287,305 (68.2)
Asian or Asian British	225,316 (7.2)	45,101 (12.3)	254,884 (7.4)	68,915 (5.7)	315,854 (6.9)	52,101 (3.9)	300,167 (6.2)
Black	67,319 (2.1)	8099 (2.2)	76,622 (2.2)	21,399 (1.8)	103,117 (2.3)	18,340 (1.4)	105,322 (2.2)
Mixed	34,748 (1.1)	4298 (1.2)	39,872 (1.2)	13,937 (1.2)	56,644 (1.2)	14,077 (1.1)	58,527 (1.2)
Other	60,935 (1.9)	5502 (1.5)	70,427 (2.0)	14,910 (1.2)	102,585 (2.2)	19,425 (1.5)	105,007 (2.2)
Unknown	837,550 (26.7)	89,236 (24.4)	888,265 (25.8)	299,145 (24.8)	1,126,384 (24.6)	250,152 (18.9)	962,542 (20.0)
**IMD**							
1 (most deprived)	795,739 (25.3)	98,399 (26.9)	877,583 (25.5)	257,992 (21.4)	1,027,467 (22.5)	212,044 (16.0)	929,649 (19.3)
2	630,189 (20.1)	76,874 (21.0)	691,131 (20.1)	243,334 (20.2)	926,135 (20.3)	251,692 (19.0)	968,303 (20.1)
3	605,759 (19.3)	68,887 (18.9)	661,150 (19.2)	248,696 (20.6)	935,067 (20.5)	292,131 (22.1)	1,047,914 (21.7)
4	57,9614 (18.5)	64,064 (17.5)	633,059 (18.4)	235,473 (19.5)	877,170 (19.2)	286,538 (21.7)	975,576 (20.2)
5 (least deprived)	52,9025 (16.8)	57,190 (15.7)	576,618 (16.8)	221,067 (18.3)	805,623 (17.6)	278,915 (21.1)	897,421 (18.6)
**Region**							
East	432,551 (13.8)	50,246 (13.8)	473,928 (13.8)	240,170 (19.9)	910,567 (19.9)	327,096 (24.8)	1,184,981 (24.6)
East Midlands	645,218 (20.5)	75,040 (20.5)	706,370 (20.5)	227,577 (18.9)	863,884 (18.9)	222,397 (16.8)	814,919 (16.9)
London	145,264 (4.6)	17,073 (4.7)	158,844 (4.6)	56,693 (4.7)	210,623 (4.6)	71,113 (5.4)	255,997 (5.3)
North East	257,985 (8.2)	29,988 (8.2)	282,198 (8.2)	71,169 (5.9)	270,284 (5.9)	59,059 (4.5)	216,216 (4.5)
North West	426,615 (13.6)	49,623 (13.6)	467,383 (13.6)	129,934 (10.8)	494,144 (10.8)	108,857 (8.2)	399,168 (8.3)

For peer review only - http://bmjopen.bmj.com/site/about/guidelines.xhtml

South East	101,976 (3.2)	11,942 (3.3)	111,986 (3.3)	70,763 (5.9)	266,098 (5.8)	100,114 (7.6)	363,867 (7.6)
South West	220,759 (7.0)	25,641 (7.0)	241,500 (7.0)	160,678 (13.3)	608,790 (13.3)	227,731 (17.2)	833,742 (17.3)
West Midlands	175,581 (5.6)	20,559 (5.6)	192,836 (5.6)	51,737 (4.3)	194,593 (4.3)	40,880 (3.1)	147,791 (3.1)
Yorkshire & The Humber	734,370 (23.4)	85,309 (23.3)	804,496 (23.4)	197,827 (16.4)	752,486 (16.5)	164,073 (12.4)	602175 (12.5)
**Health conditions**							
Obesity	681,422 (21.7)	96,712 (26.5)	748,398 (21.8)	276,759 (22.9)	957,194 (20.9)	350,777 (26.5)	1,137,710 (23.6)
Hypertension	325,969 (10.4)	43,610 (11.9)	353,416 (10.3)	112,147 (9.3)	411,803 (9.0)	164,367 (12.4)	564,823 (11.7)
Diabetes	184,450 (5.9)	30,590 (8.4)	210,532 (6.1)	70,861 (5.9)	262,906 (5.8)	100,541 (7.6)	358,449 (7.4)
Asthma	538,657 (17.2)	68,208 (18.7)	595,763 (17.3)	236,439 (19.6)	807,618 (17.7)	279,944 (21.2)	852,586 (17.7)
Other chronic respiratory disease	55,265 (1.8)	6650 (1.8)	59,773 (1.7)	15,932 (1.3)	67,361 (1.5)	27,083 (2.0)	90,755 (1.9)
Chronic cardiac disease	79,667 (2.5)	11,039 (3.0)	86,933 (2.5)	26,320 (2.2)	100,527 (2.2)	38,906 (2.9)	127,764 (2.7)
Lung cancer	1442 (0.0)	280 (0.1)	1610 (0.0)	483 (0.0)	1764 (0.0)	1050 (0.1)	2730 (0.1)
Haematological cancer	9212 (0.3)	1316 (0.4)	10,101 (0.3)	3444 (0.3)	12,964 (0.3)	7854 (0.6)	16,142 (0.3)
Other cancer	63,987 (2.0)	7798 (2.1)	69,923 (2.0)	22,351 (1.9)	84,742 (1.9)	39,599 (3.0)	125,146 (2.6)
Chronic liver disease	14,672 (0.5)	2093 (0.6)	16,380 (0.5)	4452 (0.4)	20,860 (0.5)	7637 (0.6)	26,110 (0.5)
Other neurological disease	21,945 (0.7)	2499 (0.7)	23,730 (0.7)	6678 (0.6)	29,827 (0.7)	14,364 (1.1)	35,924 (0.7)
Organ transplant	3339 (0.1)	679 (0.2)	3591 (0.1)	1554 (0.1)	4494 (0.1)	3885 (0.3)	5257 (0.1)
Asplenia	3416 (0.1)	427 (0.1)	3675 (0.1)	1127 (0.1)	4662 (0.1)	2191 (0.2)	5446 (0.1)
HIV	5404 (0.2)	714 (0.2)	6027 (0.2)	1799 (0.1)	7973 (0.2)	3304 (0.3)	8799 (0.2)
Other permanent immunodeficiency	2338 (0.1)	336 (0.1)	2730 (0.1)	1071 (0.1)	3689 (0.1)	1869 (0.1)	4158 (0.1)
Rheumatoid arthritis / SLE / psoriasis	137,536 (4.4)	16,982 (4.6)	150,787 (4.4)	56,203 (4.7)	193,893 (4.2)	71,022 (5.4)	229,537 (4.8)
**Smoking status**							
Current	637,581 (20.3)	47,915 (13.1)	684,236 (19.9)	194,327 (16.1)	895,923 (19.6)	192,381 (14.6)	910,245 (18.9)
Former	802,263 (25.5)	103,810 (28.4)	880,390 (25.6)	358,526 (29.7)	1,189,923 (26.0)	426,041 (32.2)	1,426,432 (29.6)
Never	1,536,297 (48.9)	194,747 (53.3)	1,672,286 (48.6)	581,210 (48.2)	2,185,834 (47.8)	661,794 (50.1)	2,280,509 (47.3)
Missing	164,185 (5.2)	18,942 (5.2)	202,622 (5.9)	72,485 (6.0)	299,789 (6.6)	41,097 (3.1)	201,684 (4.2)

*age, sex and STP frequency matched with 2020 COVID-19 cohort; ^age, sex and STP frequency matched with contemporary COVID-19 cohort; HIV = human immunodeficiency virus; SLE = systemic lupus erythematosus

For peer review only

For peer review only - http://bmjopen.bmj.com/site/about/guidelines.xhtml

Table 2. Rate of first fit note per 100 person-months for each cohort by demographics

	2019	2020		2021		2022	
	General population^	COVID-19 cohort	General population^	COVID-19 cohort	General population^	COVID-19 cohort	General population^
	Rate per 100 person-months (95%CI)	Rate per 100 person-months (95%CI)	Rate per 100 person-months (95%CI)	Rate per 100 person-months (95%CI)	Rate per 100 person-months (95%CI)	Rate per 100 person-months (95%CI)	Rate per 100 person-months (95%CI)
**Total**	1.21 (1.21 - 1.21)	4.88 (4.83 - 4.93)	0.93 (0.93 - 0.93)	2.66 (2.64 - 2.67)	1.23 (1.23 - 1.23)	1.73 (1.72 - 1.73)	1.23 (1.23 - 1.23)
**Age**							
18-24 y	0.98 (0.97 - 0.99)	1.58 (1.50 - 1.65)	0.66 (0.65 - 0.66)	1.25 (1.23 - 1.28)	0.96 (0.95 - 0.98)	1.30 (1.28 - 1.33)	0.91 (0.90 - 0.92)
25-34 y	1.18 (1.16 - 1.19)	3.51 (3.42 - 3.61)	0.86 (0.85 - 0.87)	2.16 (2.13 - 2.19)	1.17 (1.16 - 1.18)	1.62 (1.60 - 1.64)	1.14 (1.13 - 1.15)
35-44 y	1.19 (1.18 - 1.20)	5.19 (5.07 - 5.31)	0.91 (0.90 - 0.92)	2.95 (2.92 - 2.99)	1.24 (1.23 - 1.25)	1.65 (1.63 - 1.66)	1.19 (1.18 - 1.20)
45-54 y	1.35 (1.34 - 1.37)	6.53 (6.40 - 6.65)	1.08 (1.07 - 1.09)	3.60 (3.55 - 3.64)	1.38 (1.37 - 1.39)	1.91 (1.90 - 1.93)	1.35 (1.34 - 1.36)
55-64 y	1.30 (1.28 - 1.31)	6.62 (6.48 - 6.75)	1.09 (1.07 - 1.10)	3.79 (3.74 - 3.85)	1.33 (1.32 - 1.35)	1.87 (1.85 - 1.89)	1.32 (1.32 - 1.33)
**Sex**							
Female	1.39 (1.38 - 1.40)	5.88 (5.80 - 5.95)	1.11 (1.10 - 1.11)	3.25 (3.22 - 3.27)	1.46 (1.46 - 1.47)	1.96 (1.95 - 1.97)	1.41 (1.41 - 1.42)
Male	0.99 (0.98 - 1.00)	3.61 (3.54 - 3.68)	0.72 (0.71 - 0.72)	2.03 (2.01 - 2.05)	0.96 (0.95 - 0.97)	1.35 (1.34 - 1.37)	0.94 (0.93 - 0.94)
**Ethnicity**							
White	1.30 (1.30 - 1.31)	5.17 (5.10 - 5.24)	1.00 (0.99 - 1.00)	2.79 (2.77 - 2.81)	1.31 (1.31 - 1.32)	1.73 (1.72 - 1.74)	1.29 (1.28 - 1.29)
Mixed	1.03 (1.01 - 1.04)	4.83 (4.69 - 4.97)	0.86 (0.84 - 0.87)	3.11 (3.05 - 3.18)	1.10 (1.08 - 1.12)	2.07 (2.02 - 2.12)	1.16 (1.14 - 1.18)
Asian / Asian British	1.24 (1.20 - 1.28)	5.51 (5.18 - 5.84)	0.91 (0.88 - 0.94)	3.47 (3.34 - 3.61)	1.29 (1.25 - 1.32)	2.22 (2.14 - 2.31)	1.42 (1.38 - 1.45)
Black	1.22 (1.16 - 1.27)	4.55 (4.11 - 4.98)	0.92 (0.88 - 0.96)	2.65 (2.51 - 2.80)	1.19 (1.15 - 1.23)	1.74 (1.66 - 1.83)	1.25 (1.21 - 1.29)
Other	0.62 (0.59 - 0.65)	3.91 (3.56 - 4.25)	0.47 (0.44 - 0.49)	2.31 (2.18 - 2.44)	0.66 (0.64 - 0.69)	1.30 (1.24 - 1.37)	0.75 (0.72 - 0.77)
Unknown	1.09 (1.08 - 1.10)	4.23 (4.13 - 4.33)	0.83 (0.82 - 0.84)	2.19 (2.16 - 2.22)	1.10 (1.09 - 1.11)	1.62 (1.60 - 1.64)	1.09 (1.08 - 1.10)
**Region**							
East Midlands	1.05 (1.03 - 1.06)	4.06 (3.94 - 4.17)	0.76 (0.75 - 0.77)	2.39 (2.36 - 2.43)	1.05 (1.04 - 1.05)	1.53 (1.51 - 1.54)	1.08 (1.07 - 1.09)
East	1.21 (1.19 - 1.22)	4.79 (4.67 - 4.91)	0.92 (0.91 - 0.93)	2.74 (2.71 - 2.78)	1.29 (1.28 - 1.30)	1.89 (1.87 - 1.91)	1.34 (1.33 - 1.35)
London	0.71 (0.69 - 0.73)	3.08 (2.90 - 3.26)	0.49 (0.48 - 0.51)	2.07 (2.01 - 2.14)	0.71 (0.69 - 0.73)	1.22 (1.19 - 1.25)	0.76 (0.74 - 0.77)
North East	1.34 (1.32 - 1.36)	5.32 (5.13 - 5.51)	1.06 (1.05 - 1.08)	2.88 (2.81 - 2.95)	1.43 (1.41 - 1.45)	2.09 (2.04 - 2.14)	1.52 (1.50 - 1.55)
North West	1.39 (1.38 - 1.41)	5.76 (5.61 - 5.92)	1.12 (1.11 - 1.14)	2.81 (2.76 - 2.86)	1.51 (1.50 - 1.53)	2.11 (2.08 - 2.14)	1.53 (1.51 - 1.54)
South East	0.96 (0.93 - 0.98)	3.73 (3.50 - 3.96)	0.72 (0.70 - 0.74)	2.24 (2.17 - 2.30)	1.00 (0.98 - 1.02)	1.44 (1.41 - 1.47)	1.03 (1.02 - 1.05)
South West	1.07 (1.05 - 1.09)	4.55 (4.37 - 4.74)	0.81 (0.79 - 0.82)	2.57 (2.52 - 2.62)	1.12 (1.11 - 1.13)	1.61 (1.59 - 1.63)	1.15 (1.14 - 1.16)

For peer review only - http://bmjopen.bmj.com/site/about/guidelines.xhtml

West Midlands	1.32 (1.29 - 1.34)	5.63 (5.38 - 5.88)	0.97 (0.95 - 0.99)	3.00 (2.92 - 3.07)	1.37 (1.34 - 1.39)	2.20 (2.15 - 2.26)	1.49 (1.46 - 1.52)
Yorkshire & The Humber	1.31 (1.30 - 1.33)	5.46 (5.34 - 5.58)	1.03 (1.02 - 1.04)	2.93 (2.89 - 2.97)	1.41 (1.39 - 1.42)	2.01 (1.98 - 2.03)	1.44 (1.43 - 1.45)
**IMD quintile**							
1 (most deprived)	1.58 (1.57 - 1.59)	5.27 (5.17 - 5.38)	1.20 (1.19 - 1.21)	3.13 (3.10 - 3.17)	1.66 (1.65 - 1.67)	2.45 (2.42 - 2.48)	1.71 (1.69 - 1.72)
2	1.33 (1.32 - 1.34)	5.07 (4.96 - 5.18)	1.02 (1.01 - 1.03)	2.84 (2.80 - 2.88)	1.35 (1.34 - 1.36)	2.01 (1.99 - 2.03)	1.38 (1.37 - 1.39)
3	1.12 (1.11 - 1.13)	4.84 (4.72 - 4.95)	0.87 (0.86 - 0.88)	2.57 (2.54 - 2.61)	1.13 (1.12 - 1.14)	1.65 (1.64 - 1.67)	1.15 (1.14 - 1.16)
4	1.00 (0.99 - 1.01)	4.65 (4.53 - 4.77)	0.77 (0.76 - 0.78)	2.38 (2.34 - 2.41)	1.02 (1.01 - 1.03)	1.50 (1.48 - 1.52)	1.03 (1.02 - 1.04)
5 (least deprived)	0.86 (0.85 - 0.88)	4.23 (4.11 - 4.35)	0.66 (0.65 - 0.67)	2.18 (2.14 - 2.22)	0.88 (0.87 - 0.89)	1.27 (1.26 - 1.29)	0.90 (0.89 - 0.91)

For peer review only

Table 3. Crude and adjusted HRs for receipt of first fit note, overall and by follow-up period post-index date

Comparison and follow-up period post-index date	Fit note rate per 100 person-months (95%CI)		Crude HR (95%CI)	Age-sex adjusted HR (95%CI)	Fully adjusted HR* (95%CI)
	COVID-19 cohort	General population			
**2020 COVID-19 cohort vs 2019 general population**					
0-30 days	9.35 (9.24 - 9.46)	1.99 (1.98 - 2.01)	4.59 (4.52 - 4.65)	4.77 (4.70 - 4.84)	4.68 (4.61 - 4.75)
0-90 days	6.20 (6.14 - 6.27)	1.48 (1.47 - 1.48)	3.59 (3.55 - 3.64)	3.69 (3.64 - 3.73)	3.63 (3.58 - 3.68)
0-150 days	5.39 (5.33 - 5.45)	1.31 (1.30 - 1.32)	3.35 (3.31 - 3.39)	3.41 (3.37 - 3.45)	3.36 (3.32 - 3.40)
Overall	4.88 (4.83 - 4.93)	1.21 (1.20 - 1.22)	3.20 (3.16 - 3.24)	3.23 (3.20 - 3.27)	3.19 (3.15 - 3.23)
**2020 COVID-19 cohort vs 2020 general population**					
0-30 days	9.35 (9.24 - 9.46)	1.57 (1.55 - 1.58)	5.83 (5.74 - 5.92)	6.14 (6.05 - 6.24)	5.94 (5.85 - 6.03)
0-90 days	6.20 (6.14 - 6.27)	1.12 (1.11 - 1.13)	4.65 (4.59 - 4.71)	4.83 (4.77 - 4.89)	4.67 (4.61 - 4.73)
0-150 days	5.39 (5.33 - 5.45)	0.99 (0.99 - 1.00)	4.35 (4.30 - 4.40)	4.46 (4.40 - 4.51)	4.32 (4.27 - 4.38)
Overall	4.88 (4.83 - 4.93)	0.93 (0.93 - 0.93)	4.13 (4.08 - 4.18)	4.19 (4.14 - 4.24)	4.07 (4.02 - 4.12)
**2021 COVID-19 cohort vs 2019 general population**					
0-30 days	6.51 (6.46 - 6.56)	1.99 (1.98 - 2.01)	3.24 (3.21 - 3.28)	3.33 (3.29 - 3.37)	3.34 (3.30 - 3.38)
0-90 days	3.50 (3.48 - 3.52)	1.48 (1.47 - 1.48)	2.27 (2.25 - 2.29)	2.39 (2.37 - 2.41)	2.38 (2.35 - 2.41)
0-150 days	2.91 (2.90 - 2.93)	1.31 (1.30 - 1.32)	2.06 (2.04 - 2.07)	2.17 (2.15 - 2.18)	2.15 (2.13 - 2.17)
Overall	2.66 (2.64 - 2.67)	1.21 (1.20 - 1.22)	1.96 (1.95 - 1.98)	2.04 (2.03 - 2.06)	2.02 (2.00 - 2.04)
**2021 COVID-19 cohort vs 2021 general population**					
0-30 days	6.51 (6.46 - 6.56)	1.97 (1.96 - 1.99)	3.27 (3.24 - 3.31)	3.43 (3.40 - 3.47)	3.37 (3.33 - 3.40)
0-90 days	3.50 (3.48 - 3.52)	1.47 (1.46 - 1.48)	2.29 (2.28 - 2.31)	2.40 (2.38 - 2.42)	2.35 (2.33 - 2.37)
0-150 days	2.91 (2.90 - 2.93)	1.32 (1.31 - 1.33)	2.07 (2.06 - 2.09)	2.17 (2.16 - 2.19)	2.12 (2.10 - 2.13)
Overall	2.66 (2.64 - 2.67)	1.23 (1.22 - 1.23)	1.97 (1.96 - 1.99)	2.06 (2.04 - 2.07)	1.99 (1.98 - 2.01)
**2022 COVID-19 cohort vs 2019 general population**					

For peer review only - http://bmjopen.bmj.com/site/about/guidelines.xhtml

Comparison and follow-up period post-index date	Fit note rate per 100 person-months (95%CI)		Crude HR (95%CI)	Age-sex adjusted HR (95%CI)	Fully adjusted HR* (95%CI)
	COVID-19 cohort	General population			
0-30 days	4.87 (4.83 - 4.91)	1.99 (1.98 - 2.01)	2.46 (2.43 - 2.48)	2.14 (2.11 - 2.17)	2.27 (2.24 - 2.30)
0-90 days	2.57 (2.55 - 2.58)	1.48 (1.47 - 1.48)	1.79 (1.77 - 1.81)	1.63 (1.62 - 1.65)	1.73 (1.72 - 1.75)
0-150 days	2.05 (2.04 - 2.06)	1.31 (1.30 - 1.32)	1.65 (1.64 - 1.66)	1.53 (1.52 - 1.55)	1.63 (1.61 - 1.64)
Overall	1.73 (1.72 - 1.73)	1.21 (1.20 - 1.22)	1.56 (1.55 - 1.57)	1.46 (1.45 - 1.47)	1.55 (1.54 - 1.57)
**2022 COVID-19 cohort vs 2022 general population**					
0-30 days	4.87 (4.83 - 4.91)	2.09 (2.07 - 2.10)	2.35 (2.32 - 2.37)	2.35 (2.33 - 2.38)	2.37 (2.34 - 2.39)
0-90 days	2.57 (2.55 - 2.58)	1.53 (1.52 - 1.54)	1.73 (1.71 - 1.74)	1.73 (1.72 - 1.74)	1.73 (1.73 - 1.76)
0-150 days	2.05 (2.04 - 2.06)	1.34 (1.39 - 1.35)	1.62 (1.60 - 1.63)	1.62 (1.61 - 1.63)	1.64 (1.62 - 1.65)
Overall	1.73 (1.72 - 1.73)	1.23 (1.22 - 1.23)	1.55 (1.54 - 1.56)	1.55 (1.54 - 1.56)	1.57 (1.56 - 1.58)

^General population age, sex and STP frequency matched.

*Fully adjusted models include age, sex, IMD quintile, region, ethnicity, obesity, smoking status, hypertension, diabetes, chronic respiratory disease, asthma, chronic cardiac disease, lung cancer, haematological cancer, other cancer, chronic liver disease,other neurological disease, organ transplant, asplenia, HIV, permanent immunodeficiency, and rheumatoid arthritis/systemic lupus erythematosus/psoriasis.

For peer review only

Figure 1. Fully adjusted hazard ratio of first fit note comparing COVID-19 cohorts to their contemporary or historical 2019 general population, stratified by demographic categories.

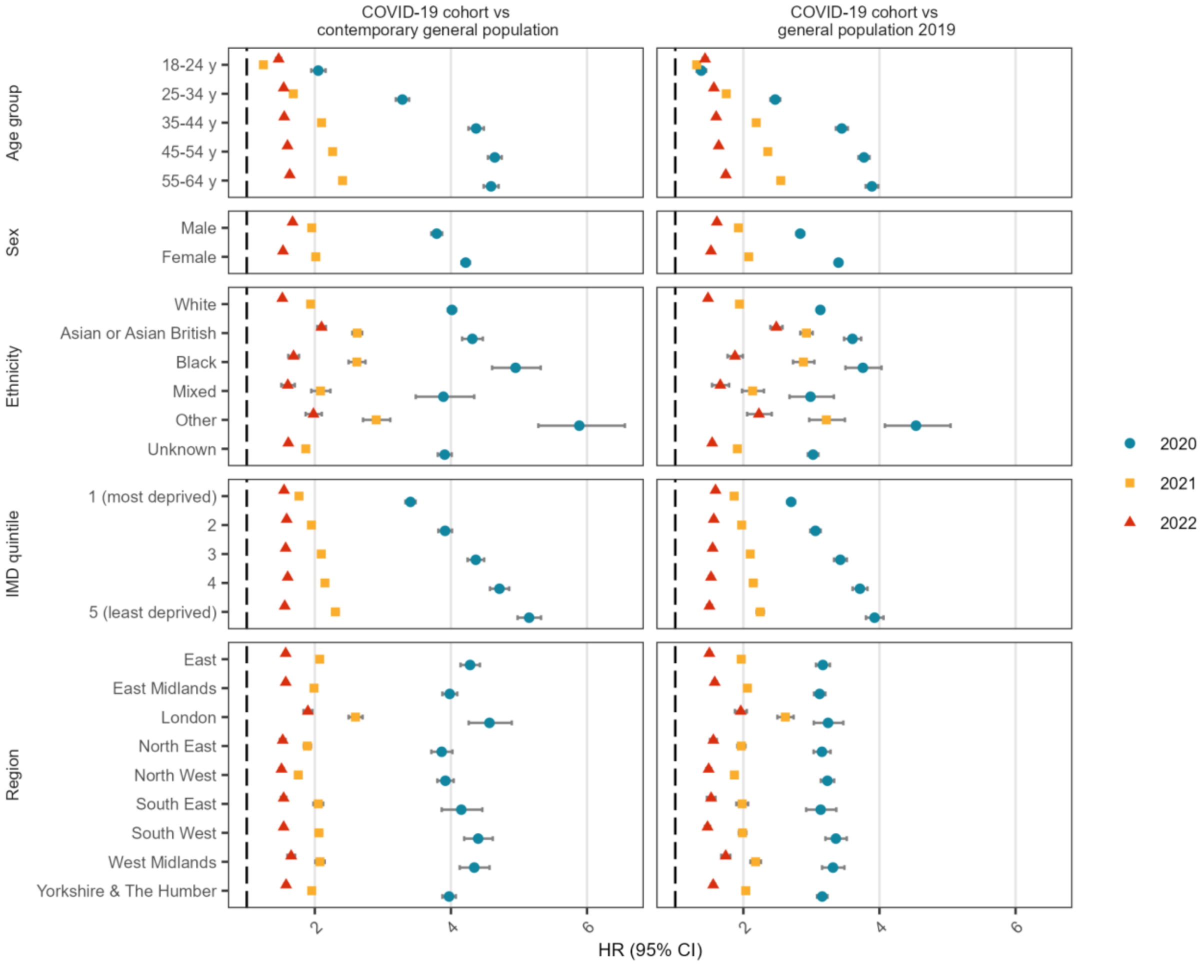

*All models are adjusted for age, sex, ethnicity, IMD quintile and region, but exclude the stratification variable. Models are additionally adjusted for obesity, smoking status, hypertension, diabetes, chronic respiratory disease, asthma, chronic cardiac disease, lung cancer, haematological cancer, other cancer, chronic liver disease, other neurological disease, organ transplant, asplenia, HIV, permanent immunodeficiency, and rheumatoid arthritis/systemic lupus erythematosus/psoriasis

For peer review only - http://bmjopen.bmj.com/site/about/guidelines.xhtml

Discussion

Summary

We found that people with a recorded positive SARS-CoV-2 test or COVID-19 diagnosis had a higher fit note rate than the general population, even after adjusting for demographics and a wide range of clinical characteristics. This increase was greatest in 2020 but continued even into 2022 despite the introduction of vaccines and other effective outpatient COVID-19 treatments. The fit note rate was highest in the first 30 days post-diagnosis, but an increased risk persisted over all follow-up in all years. These findings provide further evidence for the long-term health and economic impact of COVID-19. In contrast, people hospitalised with COVID-19 were less likely to be issued a fit note than people with pneumonia, especially in 2022, suggesting the long-term effects are not worse than comparable serious respiratory infections requiring hospitalisation.

Strengths and weaknesses

Our data come from a representative sample of 40% of the English population.(13) To better understand fit note patterns, we used COVID-19 cohorts from three different pandemic time periods, with both contemporary and historical comparators, to account for changes in preventive measures (e.g. lockdowns, vaccines) and other disruptions to working patterns (e.g. furlough) that would have impacted on receipt of fit notes over time.

However, there is bias in who gets tested or seeks care for COVID-19 which will have changed as the pandemic evolved,(14) especially after cessation of free testing in April 2022. It is unclear how this would correlate with the likelihood of requesting or requiring a fit note. To mitigate this, we relied on three different methods for identifying COVID-19 cases (positive PCR or LFT test, primary care diagnosis, hospitalisation), but many mild or asymptomatic cases, and cases among people choosing not to get tested or not to record their LFT result will be missed.

We also could not identify whether people were participating in the workforce and included everyone of working age. From 2019 to 2022, the employment rate was 62% in people 18-24 yrs, 85% in people 25-49 years, and 72% in people 50-64 years.(15) It is therefore likely that the true absolute fit note rate will be substantially attenuated, especially among the younger age groups. However, this will only impact on the relative effects if the employment rate differs substantially between the COVID-19 cohorts and general population.

Findings in context

The pandemic has evolved over time, both in the number of cases, disease severity, and demographic groups most affected. The introduction of vaccination programmes and differences in circulating variants lead to changes in severity and transmissibility of SARS-CoV-2.(16) Additionally, the ending of lockdowns and other preventive measures, cessation of free testing, and differential uptake of vaccines by demographic and clinical subgroups will have impacted on the characteristics of people testing positive for SARS-CoV-2.(17) Consistent with previously reported data,(5,18) we observed that the number of people with

For peer review only - http://bmjopen.bmj.com/site/about/guidelines.xhtml

evidence of a positive SARS-CoV-2 test was much greater in 2021 and 2022 compared with 2020. Thus, while the fit note rate was nearly twice as high in 2020, the actual number of people issued a fit note was 4.5 times higher in 2022.

In contrast, the fit note rate was lower in the general population in 2020, compared with other years. This is consistent with a previous study of NHS workers which identified a decrease in fit notes for non-COVID related episodes in 2020.(19) There are likely to be several explanations such as changes to working patterns, including furlough and remote working, which would have reduced the need for fit notes. There were also fewer circulating non-COVID-19 respiratory infections in 2020 due to public health measures.(20) It is also important to note that people who were required to isolate due to COVID-19 would be issued an isolation note, rather than a fit note and thus are not counted.

Policy implications and future research

Now several years into the pandemic, vaccines have led to reduced severity of COVID-19, but the number of daily cases remains high,(17) potentially putting many people at risk of long-term symptoms. The incidence of repeat infections is also increasing;(21) while a repeat infection appears to lead to milder acute symptoms,(22,23) other studies have found that the long-term symptom burden increases with the number of reinfections.(24,25) In our study, we did not try and link the reason for the fit note to COVID-19. However, most studies of long-term effects have focussed on surveys and self-report, which can be unrepresentative(26–28) and long COVID is not well coded in electronic health records.(11) Thus, our findings provide a different perspective on the potential long-term consequences of COVID-19 that does not rely on accurate coding of COVID-19 related symptoms.

A previous study identified that while the fit note rate decreased overall in 2020, fit notes associated with certain diagnoses, specifically asthma, respiratory conditions, and mental health increased during the COVID-19 pandemic.(19) Similarly, a study of NHS workers found that sickness absences due to psychiatric illness was higher in 2022 compared with pre-pandemic.(29) Further investigation into the duration of fit notes, as well as the diagnoses associated with the issued fit note, whether these changed over time from diagnosis, and how these compare to fit notes issued to people without COVID-19 will help shed light on the most common long-term symptoms experienced.

Conclusion

Despite undercapture of people with COVID-19 and overestimation of the number of people in the workforce, we have identified a considerable increased risk of fit notes among people with COVID-19 compared with the general population. This extends into an era of high vaccination rates and when the severity of illness from COVID-19 is decreasing. The majority of fit notes were issued within the first 30 days post-diagnosis, suggesting that most COVID-19 related sick leave is associated with the acute phase of the disease. However, a persistent increased risk up to 10 months after the illness demonstrates the ongoing health and economic impact of COVID-19.

For peer review only - http://bmjopen.bmj.com/site/about/guidelines.xhtml

Ethics approval

This study was approved by the Health Research Authority (Research Ethics Committee reference 20/LO/0651) and by the London School of Hygiene and Tropical Medicine Ethics Board (reference 21863).

Acknowledgements

We are very grateful for all the support received from the TPP Technical Operations team throughout this work, and for generous assistance from the information governance and database teams at NHS England and the NHS England Transformation Directorate

Members of OpenSAFELY Collaborative:

Sebastian CJ Bacon, Lucy Bridges, Benjamin FC Butler-Cole, Simon Davy, Iain Dillingham, David Evans, Ben Goldacre, Liam Hart, George Hickman, Peter Inglesby, Steven Maude, Jessica Morley, Amir Mehrkar, Thomas O'Dwyer, Rebecca M Smith, Tom Ward, Jon Massey, Milan Wiedemann, Christopher Bates, Jonathan Cockburn, Sam Harper, Frank Hester, John Parry

Members of National Core Studies Collaborative:

Nishi Chaturvedi, Chloe Park, Alisia Carnemolla, Dylan Williams, Anika Knueppel, Andy Boyd, Emma L Turner, Katharine M Evans, Richard Thomas, Samantha Berman, Stela McLachlan, Matthew Crane, Rebecca Whitehorn, Jacqui Oakley, Diane Foster, Hannah Woodward, Kirsteen C Campbell, Nicholas Timpson, Alex Kwong, Ana Goncalves Soares, Gareth Griffith, Renin Toms, Louise Jones, Annie Herbert, Ruth Mitchell, Tom Palmer, Jonathan Sterne, Venexia Walker, Lizzie Huntley, Laura Fox, Rachel Denholm, Rochelle Knight, Kate, Northstone, Arun, Kanagaratnam, Elsie Horne, Harriet Forbes, Teri North , Kurt, Taylor, Marwa AL Arab, Scott Walker, Jose IC Coronado, Arun S Karthikeyan, George, Ploubidis, Bettina Moltrecht, Charlotte Booth, Sam Parsons, Bozena Wielgoszewska, Charis Bridger-Staatz, Claire Steves, Ellen Thompson, Paz Garcia, Nathan Cheetham, Ruth Bowyer, Maxim Freydin, Amy Roberts, Ben Goldacre, Alex Walker, Jess Morley, William Hulme, Linda Nab, Louis Fisher, Brian MacKenna, Colm Andrews, Helen Curtis, Lisa Hopcroft, Amelia Green, Praveetha Patalay, Jane Maddock, Kishan Patel, Jean Stafford, Wels Jacques, Kate Tilling, John Macleod, Eoin McElroy, Anoop Shah Richard Silverwood, Spiros Denaxas, Robin Flaig, Daniel McCartney, Archie Campbell, Laurie Tomlinson, John Tazare, Bang Zheng, Liam Smeeth, Emily Herrett, Thomas Cowling, Kate Mansfield, Ruth E Costello, Kevin Wang, Kathryn Mansfield, Viyaasan Mahalingasivam, Ian Douglas, Sinead Langan, Sinead Brophy, Michael Parker, Jonathan Kennedy, Rosie McEachan, John Wright, Kathryn Willan, Ellena Badrick, Gillian Santorelli, Tiffany Yang, Bo Hou, Andrew Steptoe, Giorgio Di Gessa, Jingmin Zhu, Paola Zaninotto, Angela Wood, Genevieve Cezard, Samantha Ip, Tom Bolton, Alexia Sampri, Elena Rafeti, Fatima Almaghrabi, Aziz Sheikh, Syed A Shah, Vittal Katikireddi, Richard Shaw, Olivia Hamilton, Michael Green, Theocharis Kromydas, Daniel Kopasker, Felix Greaves, Robert Willans, Fiona Glen, Steve Sharp, Alun Hughes, Andrew Wong, Lee Hamill Howes, Alicja Rapala, Lidia Nigrelli, Fintan McArdle, Chelsea Beckford, Betty Raman, Richard Dobson, Amos Folarin, Callum Stewart, Yatharth Ranjan, Jd Carpentieri, Laura Sheard, Chao Fang, Sarah Baz, Andy Gibson, John Kellas, Stefan Neubauer, Stefan Piechnik, Elena Lukaschuk, Laura C Saunders, James M Wild, Stephen Smith, Peter Jezzard, Elizabeth Tunnicliffe, Zeena-Britt Sanders, Lucy Finnigan, Vanessa Ferreira, Mark Green, Rebecca Rhead, Milla Kibble, Yinghui Wei, Agnieszka Lemanska, Francisco Perez-Reche, Dominik Piehlmaier, Lucy Teece, Edward Parker

For peer review only - http://bmjopen.bmj.com/site/about/guidelines.xhtml

References

1. NHS Digital. Fit Notes Issued by GP Practices, England, September 2022 [Internet]. [cited 2023 Mar 6]. Available from: https://digital.nhs.uk/data-and-information/publications/statistical/fit-notes-issued-by-gp-practices/september-2022
2. Dorrington S, Roberts E, Mykletun A, Hatch S, Madan I, Hotopf M. Systematic review of fit note use for workers in the UK. Occup Environ Med. 2018 Jul 1;75(7):530–9.
3. Henderson M, Clark C, Stansfeld S, Hotopf M. A lifecourse approach to long-term sickness absence--a cohort study. PloS One. 2012;7(5):e36645.
4. UK Department for Work and Pensions. An evaluation of the Statement of Fitness for Work (fit note): Survey of employees (RR 840) [Internet]. [cited 2023 Mar 6]. Report No.: 840. Available from: https://www.gov.uk/government/publications/an-evaluation-of-the-statement-of-fitness-for-work-fit-note-survey-of-employees-rr-840
5. Office for National Statistics. Coronavirus (COVID-19) Infection Survey technical article: Cumulative incidence of the percentage of people who have been infected with COVID-19 by variant and age, England [Internet]. 2023 [cited 2023 Jul 6]. Available from: https://www.ons.gov.uk/peoplepopulationandcommunity/healthandsocialcare/conditionsanddiseases/articles/coronaviruscovid19infectionsurveytechnicalarticlecumulativeincidenceofthenumberofpeoplewhohavebeeninfectedwithcovid19byvariantandageengland/9february2023
6. Office for National Statistics. Cumulative COVID-19 infections time series, England [Internet]. 2023 [cited 2023 Jul 6]. Available from: https://www.ons.gov.uk/peoplepopulationandcommunity/healthandsocialcare/conditionsanddiseases/datasets/cumulativecovid19infectionstimeseriesengland
7. Office for National Statistics. Prevalence of ongoing symptoms following coronavirus (COVID-19) infection in the UK [Internet]. 2023 [cited 2023 Mar 31]. Available from: https://www.ons.gov.uk/peoplepopulationandcommunity/healthandsocialcare/conditionsanddiseases/bulletins/prevalenceofongoingsymptomsfollowingcoronaviruscovid19infectionintheuk/latest
8. Dorrington S, Carr E, Polling C, Stevelink S, Ashworth M, Roberts E, et al. Health condition at first fit note and number of fit notes: a longitudinal study of primary care records in south London. BMJ Open. 2021 Mar 1;11(3):e043889.
9. Michelen M, Manoharan L, Elkheir N, Cheng V, Dagens A, Hastie C, et al. Characterising long COVID: a living systematic review. BMJ Glob Health. 2021 Sep 1;6(9):e005427.
10. Davis HE, McCorkell L, Vogel JM, Topol EJ. Long COVID: major findings, mechanisms and recommendations. Nat Rev Microbiol. 2023 Mar;21(3):133–46.
11. Walker AJ, MacKenna B, Inglesby P, Tomlinson L, Rentsch CT, Curtis HJ, et al. Clinical coding of long COVID in English primary care: a federated analysis of 58 million patient records in situ using OpenSAFELY. Br J Gen Pract. 2021 Nov 1;71(712):e806–14.
12. Hernán MA. The Hazards of Hazard Ratios. Epidemiol Camb Mass. 2010 Jan;21(1):13–5.
13. Andrews C, Schultze A, Curtis H, Hulme W, Tazare J, Evans S, et al. OpenSAFELY: Representativeness of electronic health record platform OpenSAFELY-TPP data compared to the population of England. Wellcome Open Res. 2022;7:191.
14. Griffith GJ, Morris TT, Tudball MJ, Herbert A, Mancano G, Pike L, et al. Collider bias undermines our understanding of COVID-19 disease risk and severity. Nat Commun. 2020 Nov 12;11(1):5749.
15. Office for National Statistics. A05 SA: Employment, unemployment and economic inactivity by age group (seasonally adjusted) [Internet]. [cited 2023 May 17]. Available from: https://www.ons.gov.uk/employmentandlabourmarket/peopleinwork/employmentandemployeetypes/datasets/employmentunemploymentandeconomicinactivitybyagegroupseasonallyadjusteda05sa

For peer review only - http://bmjopen.bmj.com/site/about/guidelines.xhtml

16. Nab L, Parker EPK, Andrews CD, Hulme WJ, Fisher L, Morley J, et al. Changes in COVID-19-related mortality across key demographic and clinical subgroups in England from 2020 to 2022: a retrospective cohort study using the OpenSAFELY platform. Lancet Public Health. 2023 May 1;8(5):e364–77.
17. UK Health Security Agency. England Summary | Coronavirus (COVID-19) in the UK [Internet]. 2023 [cited 2023 Mar 6]. Available from: https://coronavirus.data.gov.uk
18. Office for National Statistics. Coronavirus (COVID-19) latest insights [Internet]. 2023 [cited 2023 Mar 31]. Available from: https://www.ons.gov.uk/peoplepopulationandcommunity/healthandsocialcare/conditionsanddiseases/articles/coronaviruscovid19latestinsights/infections
19. Edge R, van der Plaat DA, Parsons V, Coggon D, van Tongeren M, Muiry R, et al. Changing patterns of sickness absence among healthcare workers in England during the COVID-19 pandemic. J Public Health Oxf Engl. 2022 Mar 7;44(1):e42–50.
20. Kadambari S, Goldacre R, Morris E, Goldacre MJ, Pollard AJ. Indirect effects of the covid-19 pandemic on childhood infection in England: population based observational study. BMJ. 2022 Jan 12;376:e067519.
21. Coronavirus (COVID-19) Infection Survey, characteristics of people testing positive for COVID-19, UK - Office for National Statistics [Internet]. [cited 2023 May 17]. Available from: https://www.ons.gov.uk/peoplepopulationandcommunity/healthandsocialcare/conditionsanddiseases/bulletins/coronaviruscovid19infectionsurveycharacteristicsofpeopletestingpositiveforcovid19uk/21september2022
22. Abu-Raddad LJ, Chemaitelly H, Bertollini R, National Study Group for COVID-19 Epidemiology. Severity of SARS-CoV-2 Reinfections as Compared with Primary Infections. N Engl J Med. 2021 Dec 23;385(26):2487–9.
23. Mensah AA, Lacy J, Stowe J, Seghezzo G, Sachdeva R, Simmons R, et al. Disease severity during SARS-COV-2 reinfection: a nationwide study. J Infect. 2022 Apr;84(4):542–50.
24. Willyard C. Are repeat COVID infections dangerous? What the science says. Nature. 2023 Apr 26;616(7958):650–2.
25. Bowe B, Xie Y, Al-Aly Z. Acute and postacute sequelae associated with SARS-CoV-2 reinfection. Nat Med. 2022 Nov;28(11):2398–405.
26. Sudre CH, Murray B, Varsavsky T, Graham MS, Penfold RS, Bowyer RC, et al. Attributes and predictors of long COVID. Nat Med. 2021 Apr;27(4):626–31.
27. Whitaker M, Elliott J, Chadeau-Hyam M, Riley S, Darzi A, Cooke G, et al. Persistent COVID-19 symptoms in a community study of 606,434 people in England. Nat Commun. 2022 Apr 12;13(1):1957.
28. Thompson EJ, Williams DM, Walker AJ, Mitchell RE, Niedzwiedz CL, Yang TC, et al. Long COVID burden and risk factors in 10 UK longitudinal studies and electronic health records. Nat Commun. 2022 Jun 28;13(1):3528.
29. Palmer W, Rolewicz L. Nuffield Trust. 2023 [cited 2023 Jun 30]. All is not well: Sickness absence in the NHS in England. Available from: https://www.nuffieldtrust.org.uk/resource/all-is-not-well-sickness-absence-in-the-nhs-in-england

For peer review only - http://bmjopen.bmj.com/site/about/guidelines.xhtml

Administrative

Author statement

Conceptualization: AJW, LAT, JM, FG, SD; Data curation: ALS, RYP, AJW, SCJB, ID, CB, BG; Formal analysis: ALS, RYP, AJW, JT; Funding acquisition: BG; Investigation: ALS, AJW, RYP; Methodology: ALS, AJW, LAT, RYP, JM, FG, JT, KB; Project administration: AJW, LAT, AM, BG; Resources: AJW, ID, SCJB, BG; Software: ALS, RYP, AJW, JT, SCJB, ID, CB; Supervision: AJW, LAT, BMK, AM, BG; Validation: ALS, RYP, AJW; Visualisation: ALS, RYP, AJW; Writing - original draft: ALS, RYP, AJW; Writing - review & editing: All authors. All authors gave final approval of the version to be published and agree to be accountable for all aspects of the work. All authors confirm that they had full access to all the data in the study and accept responsibility to submit for publication.

Conflicts of interest

Over the past five years BG has received research funding from the Laura and John Arnold Foundation, the NHS National Institute for Health Research (NIHR), the NIHR School of Primary Care Research, the NIHR Oxford Biomedical Research Centre, the Mohn-Westlake Foundation, NIHR Applied Research Collaboration Oxford and Thames Valley, the Wellcome Trust, the Good Thinking Foundation, Health Data Research UK (HDRUK), the Health Foundation, and the World Health Organisation; he also receives personal income from speaking and writing for lay audiences on the misuse of science. CB is an employee of TPP. BMK is also employed by NHS England working on medicines policy and clinical lead for primary care medicines data.

Funding

The OpenSAFELY Platform is supported by grants from the Wellcome Trust (222097/Z/20/Z) and MRC (MR/V015737/1, MC_PC_200009, MR/W016729/1). In addition, development of OpenSAFELY has been funded by the Longitudinal Health and Wellbeing strand of the National Core Studies programme (MC_PC_20030, MC_PC_20059), the NIHR funded CONVALESCENCE programme (COV-LT-0009), NIHR (NIHR135559, COV-LT2-0073), and the Data and Connectivity National Core Study funded by UK Research and Innovation (MC_PC_20058), and Health Data Research UK (HDRUK2021.000, 2021.0157). BG's work on better use of data in healthcare more broadly is currently funded in part by: the Wellcome Trust, NIHR Oxford Biomedical Research Centre, NIHR Applied Research Collaboration Oxford and Thames Valley, the Mohn-Westlake Foundation; all University of Oxford, Bennett Institute for Applied Data Science staff are supported by BG's grants on this work. RYP is supported by the EPSRC Centre for Doctoral Training in Health Data Science (EP/S02428X/1). FG is supported by the NIHR Applied Research Collaborative for North West London. SD is supported by: a) the BHF Data Science Centre led by HDR UK (grant SP/19/3/34678), b) NIHR University College London Hospitals Biomedical Research Centre, c) BHF Accelerator Award (AA/18/6/24223), d) the NIHR-UKRI CONVALESCENCE study. KB is funded by a Wellcome Senior Research Fellowship (220283/Z/20/Z).

The views expressed are those of the authors and not necessarily those of the NIHR, NHS England, Public Health England or the Department of Health and Social Care. Funders had

For peer review only

no role in the study design, collection, analysis, and interpretation of data; in the writing of the report; and in the decision to submit the article for publication.

Information governance and ethical approval

NHS England is the data controller of the NHS England OpenSAFELY COVID-19 Service; TPP is the data processor; all study authors using OpenSAFELY have the approval of NHS England.(1) This implementation of OpenSAFELY is hosted within the TPP environment which is accredited to the ISO 27001 information security standard and is NHS IG Toolkit compliant.(2)

Patient data has been pseudonymised for analysis and linkage using industry standard cryptographic hashing techniques; all pseudonymised datasets transmitted for linkage onto OpenSAFELY are encrypted; access to the NHS England OpenSAFELY COVID-19 service is via a virtual private network (VPN) connection; the researchers hold contracts with NHS England and only access the platform to initiate database queries and statistical models; all database activity is logged; only aggregate statistical outputs leave the platform environment following best practice for anonymisation of results such as statistical disclosure control for low cell counts.(3)

The service adheres to the obligations of the UK General Data Protection Regulation (UK GDPR) and the Data Protection Act 2018. The service previously operated under notices initially issued in February 2020 by the the Secretary of State under Regulation 3(4) of the Health Service (Control of Patient Information) Regulations 2002 (COPI Regulations), which required organisations to process confidential patient information for COVID-19 purposes; this set aside the requirement for patient consent.(4) As of 1 July 2023, the Secretary of State has requested that NHS England continue to operate the Service under the COVID-19 Directions 2020.(5) In some cases of data sharing, the common law duty of confidence is met using, for example, patient consent or support from the Health Research Authority Confidentiality Advisory Group.(6)

Taken together, these provide the legal bases to link patient datasets using the service. GP practices, which provide access to the primary care data, are required to share relevant health information to support the public health response to the pandemic, and have been informed of how the service operates.

(1) NHS Digital. The NHS England OpenSAFELY COVID-19 service - privacy notice [Internet]. 2023 [cited 2023 Jul 5]. Available from: https://digital.nhs.uk/coronavirus/coronavirus-covid-19-response-information-governance-hub/the-nhs-england-opensafely-covid-19-service-privacy-notice

(2) NHS Digital. NHS Digital. 2023 [cited 2023 Jul 5]. Data Security and Protection Toolkit. Available from: https://digital.nhs.uk/data-and-information/looking-after-information/data-security-and-information-governance/data-security-and-protection-toolkit

(3) NHS Digital [Internet]. [cited 2023 Mar 6]. ISB1523: Anonymisation Standard for Publishing Health and Social Care Data. Available from: https://digital.nhs.uk/data-and-information/information-standards/information-standards-and-data-collections-including-extractions/publications-and-notifications/standards-and-collections/isb1523-anonymisation-standard-for-publishing-health-and-social-care-data

For peer review only - http://bmjopen.bmj.com/site/about/guidelines.xhtml

(4) UK Department of Health and Social Care. GOV.UK. 2022 [cited 2023 Jul 5]. [Withdrawn] Coronavirus (COVID-19): notice under regulation 3(4) of the Health Service (Control of Patient Information) Regulations 2002 – general. Available from: https://www.gov.uk/government/publications/coronavirus-covid-19-notification-of-data-controllers-to-share-information/coronavirus-covid-19-notice-under-regulation-34-of-the-health-service-control-of-patient-information-regulations-2002-general--2

(5) NHS Digital. NHS Digital. 2022 [cited 2023 Jul 5]. Secretary of State for Health and Social Care: COVID-19 Public Health Directions 2020. Available from: https://digital.nhs.uk/about-nhs-digital/corporate-information-and-documents/directions-and-data-provision-notices/secretary-of-state-directions/covid-19-public-health-directions-2020

(6) NHS Health Research Authority. Health Research Authority. [cited 2023 Jul 5]. Confidentiality Advisory Group. Available from: https://www.hra.nhs.uk/about-us/committees-and-services/confidentiality-advisory-group/

For peer review only

Supplementary Files

Supplementary Figure 1. Source of first recorded positive SARS-CoV-2 test or COVID-19 diagnosis, by year. Some people have been identified by multiple sources and therefore the percentages within years may not add up to 100%. SGSS = Second Generation Surveillance System.

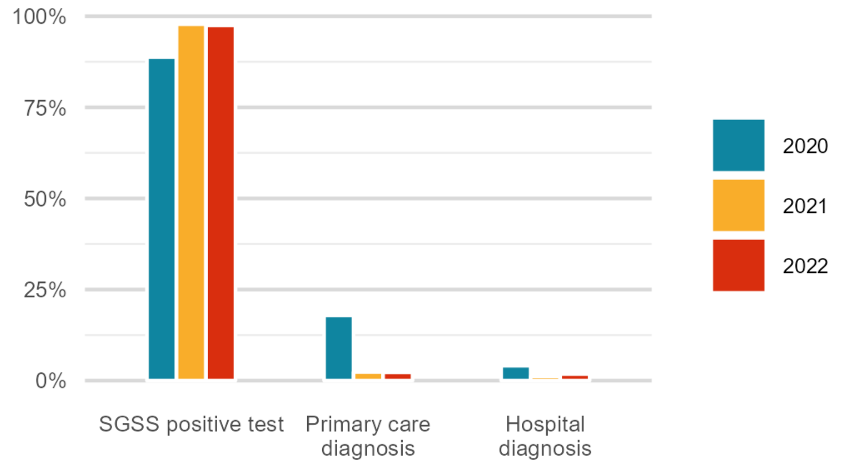

Supplementary Figure 2. Month of first recorded positive SARS-CoV-2 test or COVID-19 diagnosis by year

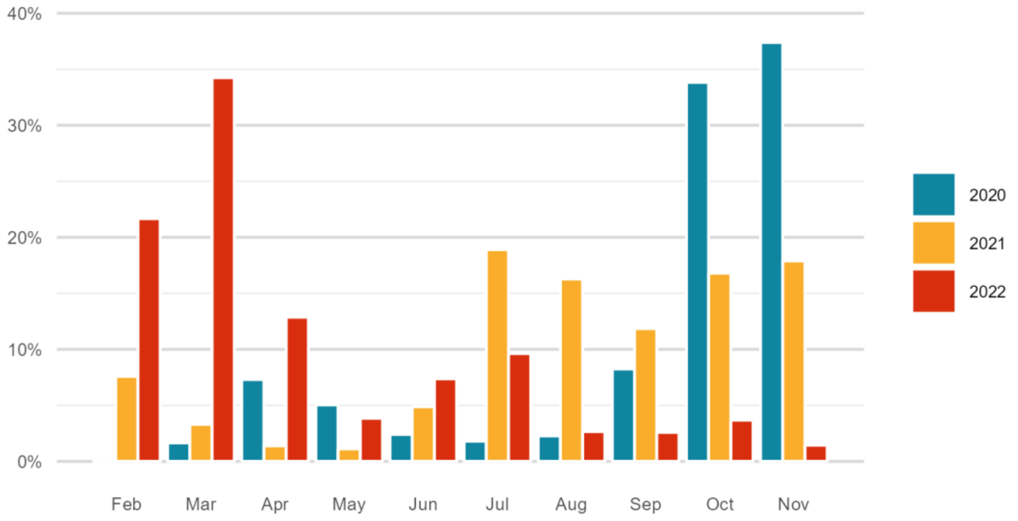

For peer review only - http://bmjopen.bmj.com/site/about/guidelines.xhtml

Supplementary Table 1. Demographic and clinical characteristics of hospitalised cohorts, 1 Feb - 30 Nov of each year. All counts rounded to nearest 7.

	COVID-19 hospitalised cohorts			Pneumonia hospitalised cohort
	2020	2021	2022	2019
	n (%)	n (%)	n (%)	n (%)
**Total**	22,015 (100.0)	30,205 (100.0)	34,692 (100.0)	29,673 (100.0)
**Age**				
18-24 y	819 (3.7)	2107 (7.0)	2485 (7.2)	1106 (3.7)
25-34 y	2429 (11.0)	5635 (18.7)	6419 (18.5)	2618 (8.8)
35-44 y	3465 (15.7)	6307 (20.9)	6006 (17.3)	4158 (14.0)
45-54 y	6328 (28.7)	7329 (24.3)	7623 (22.0)	7798 (26.3)
55-64 y	8974 (40.8)	8827 (29.2)	12,166 (35.1)	13,986 (47.1)
**Sex**				
Female	10,059 (45.7)	15,442 (51.1)	20,531 (59.2)	14,294 (48.2)
Male	11,956 (54.3)	14,763 (48.9)	14,161 (40.8)	15,379 (51.8)
**Ethnicity**				
White	12,376 (56.2)	18,305 (60.6)	24,542 (70.7)	19,817 (66.8)
Asian or Asian British	3395 (15.4)	3374 (11.2)	2289 (6.6)	1589 (5.4)
Black	1008 (4.6)	1260 (4.2)	805 (2.3)	553 (1.9)
Mixed	343 (1.6)	532 (1.8)	406 (1.2)	217 (0.7)
Other	532 (2.4)	623 (2.1)	511 (1.5)	273 (0.9)
Unknown	4361 (19.8)	6118 (20.3)	6146 (17.7)	7217 (24.3)
**IMD**				
1 (most deprived)	7077 (32.1)	10,199 (33.8)	9478 (27.3)	8974 (30.2)
2	5040 (22.9)	6839 (22.6)	7525 (21.7)	6636 (22.4)
3	3962 (18.0)	5446 (18.0)	6951 (20.0)	5775 (19.5)
4	3269 (14.8)	4319 (14.3)	5957 (17.2)	4662 (15.7)
5 (least deprived)	2667 (12.1)	3395 (11.2)	4774 (13.8)	3626 (12.2)
**Region**				
East	3885 (17.6)	5341 (17.7)	7238 (20.9)	5866 (19.8)
East Midlands	4375 (19.9)	6090 (20.2)	6776 (19.5)	5698 (19.2)
London	1680 (7.6)	1715 (5.7)	1848 (5.3)	1624 (5.5)
North East	1568 (7.1)	2044 (6.8)	2219 (6.4)	1715 (5.8)
North West	2457 (11.2)	3339 (11.1)	3542 (10.2)	2926 (9.9)
South East	854 (3.9)	1421 (4.7)	2359 (6.8)	1701 (5.7)
South West	1631 (7.4)	3080 (10.2)	4326 (12.5)	3857 (13.0)
West Midlands	1547 (7.0)	2037 (6.7)	1771 (5.1)	1463 (4.9)
Yorkshire & The Humber	4025 (18.3)	5145 (17.0)	4613 (13.3)	4816 (16.2)
**Health conditions**				
Obesity	9870 (44.8)	12,600 (41.7)	11,452 (33.0)	9877 (33.3)
Hypertension	6594 (30.0)	6552 (21.7)	8435 (24.3)	8386 (28.3)
Diabetes	5726 (26.0)	5817 (19.3)	6846 (19.7)	6146 (20.7)
Asthma	4907 (22.3)	6755 (22.4)	8694 (25.1)	7581 (25.5)
Other chronic respiratory disease	1645 (7.5)	1722 (5.7)	3304 (9.5)	5460 (18.4)
Chronic cardiac disease	2324 (10.6)	2198 (7.3)	3780 (10.9)	4095 (13.8)
Lung cancer	154 (0.7)	126 (0.4)	301 (0.9)	735 (2.5)
Haematological cancer	371 (1.7)	469 (1.6)	1162 (3.3)	882 (3.0)
Other cancer	1323 (6.0)	1358 (4.5)	3199 (9.2)	3325 (11.2)
Chronic liver disease	623 (2.8)	595 (2.0)	1582 (4.6)	1477 (5.0)
Other neurological disease	651 (3.0)	595 (2.0)	1841 (5.3)	1260 (4.2)
Organ transplant	280 (1.3)	455 (1.5)	1253 (3.6)	490 (1.7)
Asplenia	77 (0.3)	91 (0.3)	203 (0.6)	189 (0.6)
HIV	14 (0.1)	14 (0.0)	49 (0.1)	21 (0.1)

For peer review only - http://bmjopen.bmj.com/site/about/guidelines.xhtml

Other permanent immunodeficiency	49 (0.2)	70 (0.2)	196 (0.6)	91 (0.3)
Rheumatoid arthritis / SLE / Psoriasis	1505 (6.8)	1967 (6.5)	3066 (8.8)	2569 (8.7)
**Smoking status**				
Current	3171 (14.4)	4788 (15.9)	9093 (26.2)	11,039 (37.2)
Former	7791 (35.4)	10,654 (35.3)	11,277 (32.5)	9233 (31.1)
Never	10,752 (48.8)	14,021 (46.4)	13,636 (39.3)	9058 (30.5)
Missing	301 (1.4)	742 (2.5)	679 (2.0)	336 (1.1)

^age, sex and STP frequency matched; HIV = human immunodeficiency virus; SLE = systemic lupus erythematosus

For peer review only

Supplementary Table 2. Number and percentage of people with a fit note in each cohort by demographic categories. The denominator is all people within each category. All counts rounded to the nearest 7.

	2021	2020		2021		2022	
	General population*	COVID-19 cohort	General population^	COVID-19 cohort	General population^	COVID-19 cohort	General population^
	n (%)	n (%)	n (%)	n (%)	n (%)	n (%)	n (%)
**Total**	181,167 (5.8)	34,377 (9.4)	153,447 (4.5)	102,949 (8.5)	259,812 (5.7)	152,859 (11.6)	273,889 (5.7)
**Age**							
18-24 y	23,807 (4.8)	1687 (2.7)	17,556 (3.2)	9765 (5.0)	31,458 (4.5)	10,262 (9.2)	16,499 (4.4)
25-34 y	37,212 (5.6)	5264 (6.8)	30,051 (4.1)	20,307 (7.7)	53,704 (5.4)	29,078 (11.3)	48,804 (5.3)
35-44 y	36,575 (5.7)	7413 (9.9)	30,919 (4.4)	25,732 (8.7)	63,924 (5.7)	35,672 (11.4)	62,881 (5.5)
45-54 y	46,347 (6.4)	10,843 (12.9)	40,586 (5.2)	27,489 (10.1)	66,381 (6.4)	41,195 (12.5)	74,326 (6.2)
55-64 y	37,226 (6.0)	9177 (13.5)	34,335 (5.1)	19,649 (10.9)	44,345 (6.1)	36,652 (11.8)	71,372 (6.0)
**Sex**							
Female	114,191 (6.6)	23,191 (11.5)	100,037 (5.3)	65,037 (10.1)	164,451 (6.7)	106,610 (12.9)	193,816 (6.5)
Male	66,976 (4.8)	11,186 (6.8)	53,410 (3.5)	37,912 (6.8)	95,361 (4.5)	46,249 (9.3)	80,080 (4.4)
**Ethnicity**							
White	118,272 (6.2)	20,622 (9.7)	100,681 (4.8)	67,522 (8.6)	173,194 (6.0)	112,063 (11.6)	193,599 (5.9)
Mixed	11,060 (4.9)	4662 (10.3)	10,409 (4.1)	8169 (11.9)	16,219 (5.1)	7000 (13.4)	16,310 (5.4)
Asian / Asian British	3955 (5.9)	1078 (13.3)	3325 (4.3)	2632 (12.3)	6160 (6.0)	2576 (14.0)	6895 (6.5)
Black	2016 (5.8)	420 (9.8)	1764 (4.4)	1274 (9.1)	3108 (5.5)	1624 (11.5)	3381 (5.8)
Other	1820 (3.0)	490 (8.9)	1596 (2.3)	1211 (8.1)	3241 (3.2)	1701 (8.8)	3689 (3.5)
Unknown	44,051 (5.3)	7112 (8.0)	35,672 (4.0)	22,127 (7.4)	57,890 (5.1)	27,888 (11.1)	50,022 (5.2)
**Region**							
East Midlands	21693 (5.0)	4767 (9.5)	17,423 (3.7)	17,577 (7.3)	44,625 (4.9)	34,139 (10.4)	59,633 (5.0)
East	37,121 (5.8)	6608 (8.8)	31,213 (4.4)	20426 (9.0)	51,457 (6.0)	27,720 (12.5)	50,442 (6.2)
London	4998 (3.4)	1148 (6.7)	3801 (2.4)	4053 (7.1)	7084 (3.4)	5957 (8.4)	9163 (3.6)
North East	16,373 (6.3)	3087 (10.3)	14,329 (5.1)	6979 (9.8)	17,724 (6.6)	7833 (13.3)	15,092 (7.0)
North West	28,168 (6.6)	5313 (10.7)	25,025 (5.4)	12,558 (9.7)	33,950 (6.9)	14,805 (13.6)	27,965 (7.0)

For peer review only

South East	4676 (4.6)	987 (8.3)	3913 (3.5)	4599 (6.5)	12,425 (4.7)	9996 (10.0)	17,430 (4.8)
South West	11,354 (5.1)	2254 (8.8)	9401 (3.9)	11,382 (7.1)	31,787 (5.2)	25,158 (11.0)	44,107 (5.3)
West Midlands	10,941 (6.2)	2002 (9.7)	8939 (4.6)	5481 (10.6)	12,229 (6.3)	5705 (14.0)	10,094 (6.8)
Yorkshire & The Humber	45,843 (6.2)	8204 (9.6)	39,403 (4.9)	19,887 (10.1)	48,524 (6.4)	21,539 (13.1)	39,956 (6.6)
**IMD quintile**							
1 (most deprived)	59,052 (7.4)	9989 (10.2)	50,148 (5.7)	28,728 (11.1)	77,714 (7.6)	33,229 (15.7)	72,597 (7.8)
2	39,767 (6.3)	7581 (9.9)	33,586 (4.9)	22,827 (9.4)	57,729 (6.2)	33,320 (13.2)	61,446 (6.3)
3	32,417 (5.4)	6503 (9.4)	27,699 (4.2)	20,083 (8.1)	49,301 (5.3)	32,578 (11.2)	55,762 (5.3)
4	27,853 (4.8)	5698 (8.9)	23,506 (3.7)	17,087 (7.3)	41,846 (4.8)	29,288 (10.2)	46,494 (4.8)
5 (least deprived)	22,078 (4.2)	4606 (8.1)	18,494 (3.2)	14,224 (6.4)	33,222 (4.1)	24,444 (8.8)	37,597 (4.2)

*age, sex and STP frequency matched with 2020 COVID-19 cohort; ^age, sex and STP frequency matched with contemporary COVID-19 cohort

For peer review only

Supplementary Table 3. Number of people issued a fit note and rate of first fit note per 100 person-months for hospitalised cohorts by demographic categories. The denominator for the percentages is all people within each category. All counts rounded to the nearest 7.

	COVID-19 hospitalised cohorts						Pneumonia hospitalised cohort 2019	
	2020		2021		2022		2019	
	n (%)	Rate per 100 person-months (95%CI)	n (%)	Rate per 100 person-months (95%CI)	n (%)	Rate per 100 person-months (95%CI)	n (%)	Rate per 100 person-months (95%CI)
**Total**	4606 (20.9)	6.78 (6.59 - 6.98)	7231 (23.9)	7.19 (7.03 - 7.36)	6664 (19.2)	4.13 (4.03 - 4.22)	7448 (25.1)	7.17 (7.01 - 7.34)
**Age**								
18-24 y	91 (11.1)	3.58 (2.84 - 4.31)	336 (15.9)	4.26 (3.80 - 4.71)	392 (15.8)	3.02 (2.72 - 3.31)	266 (24.1)	6.29 (5.53 - 7.04)
25-34 y	399 (16.4)	4.98 (4.49 - 5.47)	1050 (18.6)	5.27 (4.95 - 5.58)	1029 (16.0)	3.04 (2.86 - 3.23)	798 (30.5)	8.46 (7.88 - 9.05)
35-44 y	791 (18.1)	6.88 (6.41 - 7.36)	1589 (25.2)	7.75 (7.37 - 8.13)	1218 (20.3)	4.14 (3.90 - 4.37)	1253 (30.1)	8.66 (8.18 - 9.13)
45-54 y	1498 (23.7)	7.50 (7.12 - 7.88)	2107 (28.7)	9.07 (8.68 - 9.46)	1722 (22.6)	5.16 (4.92 - 5.41)	2100 (26.9)	7.76 (7.42 - 8.09)
55-64 y	1834 (20.4)	7.09 (6.76 - 7.41)	2149 (24.3)	7.42 (7.11 - 7.73)	2310 (19.0)	4.45 (4.27 - 4.63)	3031 (21.7)	6.23 (6.01 - 6.46)
**Sex**								
Female	2212 (22.0)	7.29 (6.99 - 7.60)	3430 (22.2)	6.64 (6.42 - 6.86)	3731 (18.2)	3.74 (3.62 - 3.86)	3556 (24.9)	7.00 (6.77 - 7.23)
Male	2394 (20.0)	6.37 (6.12 - 6.63)	3801 (25.7)	7.78 (7.53 - 8.03)	2940 (20.8)	4.75 (4.58 - 4.92)	3899 (25.4)	7.36 (7.12 - 7.59)
**Ethnicity**								
White	2611 (21.1)	6.99 (6.73 - 7.26)	4459 (24.4)	7.63 (7.41 - 7.86)	4704 (19.2)	4.12 (4.00 - 4.23)	4914 (24.8)	7.08 (6.88 - 7.28)
Mixed	651 (19.2)	6.28 (5.80 - 6.76)	721 (21.4)	5.36 (4.97 - 5.75)	427 (18.7)	4.07 (3.68 - 4.45)	350 (22.0)	5.76 (5.16 - 6.37)
Asian / Asian British	245 (24.3)	6.79 (5.94 - 7.64)	336 (26.7)	8.30 (7.41 - 9.18)	175 (21.7)	4.78 (4.07 - 5.49)	147 (26.6)	7.38 (6.19 - 8.57)
Black	77 (22.4)	6.79 (5.28 - 8.31)	112 (21.1)	6.28 (5.12 - 7.44)	77 (19.0)	3.89 (3.02 - 4.76)	56 (25.8)	7.17 (5.29 - 9.04)
Other	105 (19.7)	5.41 (4.37 - 6.44)	119 (19.1)	4.81 (3.94 - 5.67)	84 (16.4)	3.57 (2.81 - 4.34)	63 (23.1)	7.27 (5.48 - 9.06)
Unknown	910 (20.9)	6.74 (6.30 - 7.18)	1484 (24.3)	7.30 (6.92 - 7.67)	1204 (19.6)	4.18 (3.95 - 4.42)	1925 (26.7)	7.79 (7.44 - 8.13)
**Region**								
East Midlands	819 (21.1)	5.79 (5.39 - 6.18)	1246 (23.3)	7.35 (6.94 - 7.75)	1358 (18.8)	3.88 (3.67 - 4.09)	1512 (25.8)	7.30 (6.94 - 7.67)
East	854 (19.5)	6.39 (5.96 - 6.82)	1428 (23.4)	7.02 (6.65 - 7.38)	1372 (20.2)	4.48 (4.24 - 4.71)	1449 (25.4)	7.35 (6.97 - 7.73)
London	287 (17.1)	4.26 (3.77 - 4.75)	329 (19.2)	5.31 (4.74 - 5.89)	294 (15.9)	3.17 (2.81 - 3.54)	350 (21.6)	5.95 (5.33 - 6.57)
North East	322 (20.5)	7.79 (6.94 - 8.63)	497 (24.3)	6.91 (6.30 - 7.51)	392 (17.7)	3.81 (3.43 - 4.18)	420 (24.5)	7.11 (6.43 - 7.79)
North West	581 (23.6)	9.11 (8.37 - 9.85)	861 (25.8)	7.49 (6.99 - 7.99)	721 (20.4)	4.55 (4.22 - 4.88)	735 (25.1)	7.32 (6.79 - 7.85)
South East	168 (19.7)	5.20 (4.42 - 5.99)	329 (23.2)	6.87 (6.13 - 7.62)	427 (18.1)	3.65 (3.30 - 3.99)	434 (25.5)	7.16 (6.48 - 7.83)

South West	413 (25.3)	8.04 (7.27 - 8.82)	735 (23.9)	8.69 (8.06 - 9.32)	819 (18.9)	3.91 (3.64 - 4.18)	994 (25.8)	7.14 (6.69 - 7.58)
West Midlands	301 (20.7)	6.28 (5.57 - 6.99)	497 (24.4)	6.71 (6.12 - 7.30)	357 (20.2)	4.66 (4.18 - 5.15)	343 (23.4)	6.69 (5.98 - 7.40)
Yorkshire & The Humber	854 (21.2)	8.58 (8.00 - 9.15)	1316 (25.6)	7.45 (7.05 - 7.85)	938 (20.3)	4.64 (4.34 - 4.94)	1211 (25.1)	7.35 (6.93 - 7.76)
**IMD quintile**								
1 (most deprived)	1337 (18.9)	6.50 (6.16 - 6.85)	2352 (23.1)	6.54 (6.27 - 6.80)	1743 (20.6)	4.01 (3.82 - 4.20)	1967 (21.9)	6.13 (5.86 - 6.40)
2	1043 (20.7)	6.69 (6.28 - 7.10)	1645 (24.1)	7.35 (7.00 - 7.70)	1449 (19.3)	4.18 (3.96 - 4.39)	1673 (25.2)	7.16 (6.82 - 7.50)
3	896 (22.6)	7.22 (6.75 - 7.69)	1379 (25.3)	7.74 (7.33 - 8.14)	1330 (19.1)	4.05 (3.83 - 4.26)	1512 (26.2)	7.51 (7.13 - 7.88)
4	756 (23.1)	7.29 (6.77 - 7.81)	1057 (24.5)	7.71 (7.24 - 8.17)	1183 (19.9)	4.24 (3.99 - 4.48)	1288 (27.6)	8.08 (7.64 - 8.52)
5 (least deprived)	574 (21.5)	6.40 (5.88 - 6.93)	798 (23.5)	7.51 (6.99 - 8.03)	952 (19.9)	4.22 (3.95 - 4.49)	1015 (28.0)	8.25 (7.75 - 8.76)

For peer review only

Supplementary Table 4. Crude and adjusted HRs for receipt of first fit note for comparisons of hospitalised cohorts, overall and by follow-up time post-diagnosis

Time period	Fit note rate per 100 person-months (95%CI)		Crude HR (95%CI)	Age-sex adjusted HR (95%CI)	Fully adjusted HR* (95%CI)
	COVID-19 cohort	Comparator cohort			
**2020 COVID-19 cohort vs 2019 pneumonia cohort**					
0-30 days	18.84 (18.18 - 19.49)	22.06 (21.46 - 22.66)	0.85 (0.81 - 0.88)	0.83 (0.78 - 0.88)	0.72 (0.68 - 0.77)
0-90 days	11.44 (11.09 - 11.79)	12.47 (12.17 - 12.76)	0.86 (0.83 - 0.89)	0.83 (0.78 - 0.88)	0.74 (0.69 - 0.78)
0-150 days	8.66 (8.40 - 8.92)	9.28 (9.06 - 9.49)	0.88 (0.85 - 0.92)	0.84 (0.79 - 0.88)	0.75 (0.71 - 0.80)
Overall	6.78 (6.59 - 6.98)	7.17 (7.01 - 7.34)	0.90 (0.86 - 0.93)	0.85 (0.80 - 0.89)	0.77 (0.73 - 0.81)
**2021 COVID-19 cohort vs 2019 pneumonia cohort**					
0-30 days	21.39 (20.81 - 21.96)	22.06 (21.46 - 22.66)	0.97 (0.93 - 1.00)	0.98 (0.92 - 1.03)	0.83 (0.78 - 0.88)
0-90 days	11.65 (11.37 - 11.93)	12.47 (12.17 - 12.76)	0.93 (0.90 - 0.96)	0.92 (0.88 - 0.97)	0.81 (0.77 - 0.85)
0-150 days	9.03 (8.82 - 9.24)	9.28 (9.06 - 0.49)	0.93 (0.90 - 0.96)	0.91 (0.87 - 0.96)	0.80 (0.77 - 0.85)
Overall	7.19 (7.03 - 7.36)	7.17 (7.01 - 7.34)	0.94 (0.91 - 0.97)	0.92 (0.87 - 0.96)	0.81 (0.77 - 0.86)
**2022 COVID-19 cohort vs 2019 pneumonia cohort**					
0-30 days	12.92 (12.52 - 13.32)	22.06 (21.46 - 22.66)	0.59 (0.57 - 0.62)	0.58 (0.54 - 0.61)	0.56 (0.53 - 0.60)
0-90 days	7.05 (6.87 - 7.24)	12.47 (12.17 - 12.75)	0.61 (0.59 - 0.62)	0.59 (0.56 - 0.62)	0.58 (0.56 - 0.61)
0-150 days	5.22 (5.09 - 5.35)	9.28 (9.06 - 9.49)	0.63 (0.61 - 0.65)	0.61 (0.58 - 0.64)	0.60 (0.58 - 0.63)
Overall	4.13 (4.03 - 4.22)	7.17 (7.01 - 7.34)	0.65 (0.63 - 0.67)	0.63 (0.60 - 0.66)	0.62 (0.59 - 0.65)

*Fully adjusted models include age, sex, IMD quintile, region, ethnicity, obesity, smoking status, hypertension, diabetes, chronic respiratory disease, asthma, chronic cardiac disease, lung cancer, haematological cancer, other cancer, chronic liver disease, other neurological disease, organ transplant, asplenia, HIV, permanent immunodeficiency, and rheumatoid arthritis/systemic lupus erythematosus/psoriasis.

For peer review only

Supplementary Table 5. Crude and adjusted hazard ratio for first fit note for all comparisons, stratified by age group.

Age group	Crude HR (95%CI)	Fully adjusted HR (95%CI)
**2020 COVID-19 cohort vs 2019 general population**		
18-24 y	1.31 (1.25 - 1.38)	1.38 (1.31 - 1.45)
25-34 y	2.44 (2.37 - 2.51)	2.47 (2.40 - 2.54)
35-44 y	3.47 (3.38 - 3.56)	3.45 (3.36 - 3.54)
45-54 y	3.83 (3.75 - 3.91)	3.77 (3.69 - 3.85)
55-64 y	4.05 (3.95 - 4.14)	3.89 (3.80 - 3.98)
**2020 COVID-19 cohort vs 2020 general population**		
18-24 y	2.01 (1.91 - 2.12)	2.05 (1.95 - 2.16)
25-34 y	3.31 (3.22 - 3.41)	3.29 (3.19 - 3.39)
35-44 y	4.46 (4.35 - 4.58)	4.37 (4.26 - 4.48)
45-54 y	4.77 (4.67 - 4.87)	4.64 (4.54 - 4.75)
55-64 y	4.80 (4.69 - 4.91)	4.59 (4.48 - 4.70)
**2021 COVID-19 cohort vs 2019 general population**		
18-24 y	1.22 (1.19 - 1.25)	1.06 (1.00 - 1.12)
25-34 y	1.71 (1.68 - 1.74)	1.31 (1.28 - 1.35)
35-44 y	2.16 (2.13 - 2.19)	1.75 (1.72 - 1.78)
45-54 y	2.26 (2.23 - 2.29)	2.19 (2.15 - 2.22)
55-64 y	2.51 (2.47 - 2.56)	2.36 (2.32 - 2.39)
**2021 COVID-19 cohort vs 2021 general population**		
18-24 y	1.25 (1.23 - 1.28)	1.24 (1.22 - 1.27)
25-34 y	1.73 (1.71 - 1.76)	1.68 (1.66 - 1.71)
35-44 y	2.13 (2.10 - 2.17)	2.10 (2.07 - 2.13)
45-54 y	2.27 (2.24 - 2.30)	2.26 (2.23 - 2.29)
55-64 y	2.48 (2.44 - 2.52)	2.41 (2.37 - 2.45)
**2022 COVID-19 cohort vs 2019 general population**		
18-24 y	1.41 (1.38 - 1.45)	1.44 (1.40 - 1.47)
25-34 y	1.49 (1.47 - 1.51)	1.57 (1.54 - 1.59)
35-44 y	1.52 (1.50 - 1.54)	1.60 (1.57 - 1.63)
45-54 y	1.55 (1.53 - 1.57)	1.64 (1.61 - 1.66)
55-64 y	1.60 (1.57 - 1.62)	1.74 (1.71 - 1.77)
**2022 COVID-19 cohort vs 2022 general population**		
18-24 y	1.53 (1.49 - 1.57)	1.47 (1.43 - 1.51)

For peer review only

For peer review only - http://bmjopen.bmj.com/site/about/guidelines.xhtml

25-34 y	1.55 (1.52 - 1.57)	1.54 (1.52 - 1.57)
35-44 y	1.53 (1.51 - 1.55)	1.55 (1.53 - 1.57)
45-54 y	1.57 (1.55 - 1.59)	1.60 (1.58 - 1.62)
55-64 y	1.56 (1.54 - 1.59)	1.63 (1.61 - 1.65)
**2020 hospitalised COVID-19 cohort vs 2019 hospitalised pneumonia cohort**		
18-24 y	0.50 (0.39 - 0.63)	0.48 (0.37 - 0.61)
25-34 y	0.54 (0.48 - 0.61)	0.54 (0.47 - 0.61)
35-44 y	0.77 (0.71 - 0.84)	0.73 (0.66 - 0.80)
45-54 y	0.94 (0.88 - 1.00)	0.81 (0.76 - 0.88)
55-64 y	0.50 (0.39 - 0.63)	0.94 (0.88 - 0.99)
**2021 hospitalised COVID-19 cohort vs 2019 hospitalised pneumonia cohort**		
18-24 y	0.64 (0.54 - 0.75)	0.61 (0.51 - 0.72)
25-34 y	0.57 (0.52 - 0.63)	0.57 (0.51 - 0.63)
35-44 y	0.82 (0.76 - 0.88)	0.74 (0.68 - 0.80)
45-54 y	1.09 (1.03 - 1.16)	0.94 (0.88 - 1.01)
55-64 y	1.16 (1.09 - 1.22)	0.61 (0.51 - 0.72)
**2022 hospitalised COVID-19 cohort vs 2019 hospitalised pneumonia cohort**		
18-24 y	0.54 (0.46 - 0.63)	0.54 (0.46 - 0.64)
25-34 y	0.42 (0.39 - 0.47)	0.44 (0.39 - 0.48)
35-44 y	0.56 (0.52 - 0.61)	0.56 (0.52 - 0.61)
45-54 y	0.73 (0.68 - 0.78)	0.72 (0.68 - 0.77)
55-64 y	0.76 (0.72 - 0.80)	0.78 (0.74 - 0.82)

*Fully adjusted models include age, sex, IMD quintile, region, ethnicity, obesity, smoking status, hypertension, diabetes, chronic respiratory disease, asthma, chronic cardiac disease, lung cancer, haematological cancer, other cancer, chronic liver disease, other neurological disease, organ transplant, asplenia, HIV, permanent immunodeficiency, and rheumatoid arthritis/systemic lupus erythematosus/psoriasis.

For peer review only

Supplementary Table 6. Crude and adjusted hazard ratio of first fit note for all comparisons, stratified by sex

Sex	Crude HR (95%CI)	Fully adjusted HR (95%CI)
**2020 COVID-19 cohort vs 2022 general population**		
Female	3.39 (3.34 - 3.44)	3.40 (3.35 - 3.45)
Male	2.86 (2.80 - 2.92)	2.84 (2.78 - 2.89)
**2020 COVID-19 cohort vs 2020 general population**		
Female	4.22 (4.16 - 4.29)	4.22 (4.15 - 4.28)
Male	3.91 (3.83 - 4.00)	3.79 (3.71 - 3.87)
**2021 COVID-19 cohort vs 2019 general population**		
Female	2.08 (2.06 - 2.10)	2.08 (2.06 - 2.10)
Male	1.84 (1.82 - 1.86)	1.93 (1.90 - 1.95)
**2021 COVID-19 cohort vs 2021 general population**		
Female	2.01 (1.99 - 2.03)	2.01 (2.00 - 2.03)
Male	1.93 (1.91 - 1.95)	1.95 (1.93 - 1.98)
**2022 COVID-19 cohort vs 2019 general population**		
Female	1.54 (1.52 - 1.55)	1.52 (1.51 - 1.54)
Male	1.50 (1.48 - 1.52)	1.61 (1.59 - 1.64)
**2022 COVID-19 cohort vs 2022 general population**		
Female	1.53 (1.51 - 1.54)	1.53 (1.52 - 1.54)
Male	1.60 (1.58 - 1.62)	1.67 (1.65 - 1.69)
**2029 hospitalised COVID-19 cohort vs 2019 hospitalised pneumonia cohort**		
Female	0.95 (0.91 - 1.01)	0.78 (0.72 - 0.85)
Male	0.85 (0.81 - 0.89)	0.75 (0.69 - 0.81)
**2021 hospitalised COVID-19 cohort vs 2019 hospitalised pneumonia cohort**		
Female	0.87 (0.83 - 0.92)	0.76 (0.71 - 0.82)
Male	1.02 (0.97 - 1.06)	0.88 (0.82 - 0.94)
**2022 hospitalised COVID-19 cohort vs 2019 hospitalised pneumonia cohort**		
Female	0.61 (0.59 - 0.64)	0.57 (0.53 - 0.61)
Male	0.70 (0.67 - 0.74)	0.70 (0.66 - 0.75)

*Fully adjusted models include age, sex, IMD quintile, region, ethnicity, obesity, smoking status, hypertension, diabetes, chronic respiratory disease, asthma, chronic cardiac disease, lung cancer, haematological cancer, other cancer, chronic liver disease,other neurological disease, organ transplant, asplenia, HIV, permanent immunodeficiency, and rheumatoid arthritis/systemic lupus erythematosus/psoriasis.

For peer review only - http://bmjopen.bmj.com/site/about/guidelines.xhtml

Supplementary Table 7. Crude and adjusted hazard ratio of first fit note for all comparisons, stratified by ethnicity

Ethnicity	Crude HR (95%CI)	Fully adjusted HR (95%CI)
**2020 COVID-19 cohort vs 2019 general population**		
White	3.13 (3.08 - 3.18)	3.13 (3.08 - 3.18)
Asian or Asian British	3.71 (3.59 - 3.84)	3.60 (3.48 - 3.73)
Black	3.81 (3.56 - 4.08)	3.76 (3.50 - 4.03)
Mixed	3.04 (2.73 - 3.38)	2.99 (2.68 - 3.33)
Other	5.33 (4.83 - 5.89)	4.54 (4.08 - 5.05)
Not stated	3.08 (3.00 - 3.15)	3.02 (2.95 - 3.10)
**2020 COVID-19 cohort vs 2020 general population**		
White	4.05 (3.99 - 4.11)	4.01 (3.95 - 4.08)
Asian or Asian British	4.44 (4.30 - 4.60)	4.31 (4.16 - 4.47)
Black	5.12 (4.78 - 5.49)	4.95 (4.60 - 5.32)
Mixed	4.00 (3.59 - 4.45)	3.89 (3.48 - 4.34)
Other	6.74 (6.09 - 7.46)	5.89 (5.28 - 6.56)
Not stated	4.02 (3.91 - 4.12)	3.91 (3.81 - 4.01)
**2021 COVID-19 cohort vs 2019 general population**		
White	1.89 (1.87 - 1.91)	1.94 (1.92 - 1.96)
Asian or Asian British	2.84 (2.76 - 2.92)	2.93 (2.84 - 3.02)
Black	2.59 (2.47 - 2.72)	2.88 (2.73 - 3.04)
Mixed	2.01 (1.87 - 2.15)	2.14 (1.98 - 2.30)
Other	3.42 (3.18 - 3.67)	3.22 (2.97 - 3.49)
Not stated	1.82 (1.79 - 1.85)	1.91 (1.88 - 1.94)
**2021 COVID-19 cohort vs 2021 general population**		
White	1.91 (1.90 - 1.93)	1.94 (1.92 - 1.96)
Asian or Asian British	2.69 (2.62 - 2.76)	2.62 (2.55 - 2.70)
Black	2.54 (2.42 - 2.66)	2.62 (2.50 - 2.74)
Mixed	2.09 (1.95 - 2.23)	2.08 (1.95 - 2.23)
Other	3.23 (3.03 - 3.45)	2.90 (2.71 - 3.11)
Not stated	1.84 (1.81 - 1.87)	1.87 (1.84 - 1.90)
**2022 COVID-19 cohort vs 2019 general population**		
White	1.46 (1.45 - 1.47)	1.48 (1.47 - 1.50)
Asian or Asian British	2.21 (2.15 - 2.28)	2.48 (2.40 - 2.58)
Black	1.94 (1.85 - 2.04)	1.88 (1.77 - 1.99)
Mixed	1.57 (1.47 - 1.68)	1.66 (1.54 - 1.79)

For peer review only

For peer review only - http://bmjopen.bmj.com/site/about/guidelines.xhtml

Other	2.32 (2.16 - 2.48)	2.23 (2.06 - 2.42)
Not stated	1.62 (1.59 - 1.64)	1.54 (1.51 - 1.57)
**2022 COVID-19 cohort vs 2022 general population**		
White	1.49 (1.48 - 1.50)	2.10 (2.04 - 2.16)
Asian or Asian British	1.97 (1.91 - 2.02)	1.69 (1.61 - 1.77)
Black	1.71 (1.64 - 1.80)	1.60 (1.51 - 1.71)
Mixed	1.54 (1.45 - 1.64)	1.98 (1.86 - 2.10)
Other	1.93 (1.82 - 2.04)	1.61 (1.59 - 1.63)
Not stated	1.63 (1.61 - 1.66)	2.10 (2.04 - 2.16)
**2020 hospitalised COVID-19 cohort vs 2019 hospitalised pneumonia cohort**		
White	0.93 (0.89 - 0.97)	0.78 (0.72 - 0.83)
Asian or Asian British	0.98 (0.86 - 1.12)	0.90 (0.76 - 1.08)
Black	0.97 (0.79 - 1.19)	0.80 (0.60 - 1.07)
Mixed	0.96 (0.68 - 1.35)	0.58 (0.35 - 0.94)
Other	0.83 (0.61 - 1.14)	0.77 (0.51 - 1.18)
Not stated	0.83 (0.77 - 0.90)	0.74 (0.65 - 0.83)
**2021 hospitalised COVID-19 cohort vs 2019 hospitalised pneumonia cohort**		
White	0.99 (0.95 - 1.03)	0.81 (0.76 - 0.87)
Asian or Asian British	0.94 (0.83 - 1.07)	1.02 (0.86 - 1.22)
Black	1.03 (0.85 - 1.25)	0.90 (0.69 - 1.17)
Mixed	0.83 (0.60 - 1.14)	0.55 (0.35 - 0.84)
Other	0.70 (0.52 - 0.96)	0.73 (0.47 - 1.12)
Not stated	0.88 (0.82 - 0.94)	0.76 (0.69 - 0.85)
**2022 hospitalised COVID-19 cohort vs 2019 hospitalised pneumonia cohort**		
White	0.65 (0.63 - 0.68)	0.62 (0.58 - 0.65)
Asian or Asian British	0.75 (0.65 - 0.86)	0.75 (0.63 - 0.90)
Black	0.72 (0.58 - 0.90)	0.68 (0.52 - 0.90)
Mixed	0.62 (0.44 - 0.88)	0.51 (0.32 - 0.80)
Other	0.58 (0.42 - 0.80)	0.61 (0.41 - 0.91)
Not stated	0.61 (0.57 - 0.66)	0.60 (0.54 - 0.66)

*Fully adjusted models include age, sex, IMD quintile, region, ethnicity, obesity, smoking status, hypertension, diabetes, chronic respiratory disease, asthma, chronic cardiac disease, lung cancer, haematological cancer, other cancer, chronic liver disease,other neurological disease, organ transplant, asplenia, HIV, permanent immunodeficiency, and rheumatoid arthritis/systemic lupus erythematosus/psoriasis.

For peer review only - http://bmjopen.bmj.com/site/about/guidelines.xhtml

Supplementary Table 8. Crude and adjusted hazard ratio of first fit note for all comparisons, stratified by IMD quintile

IMD quintile	Crude HR (95%CI)	Fully adjusted HR (95%CI)
**2020 COVID-19 cohort vs 2019 general population**		
1 (most deprived)	2.64 (2.59 - 2.70)	2.70 (2.64 - 2.76)
2	3.05 (2.98 - 3.13)	3.06 (2.98 - 3.14)
3	3.45 (3.36 - 3.54)	3.43 (3.33 - 3.52)
4	3.71 (3.61 - 3.82)	3.71 (3.61 - 3.82)
5 (least deprived)	3.86 (3.74 - 3.99)	3.93 (3.80 - 4.06)
**2020 COVID-19 cohort vs 2020 general population**		
1 (most deprived)	3.44 (3.36 - 3.51)	3.41 (3.33 - 3.48)
2	3.96 (3.86 - 4.06)	3.91 (3.81 - 4.02)
3	4.41 (4.29 - 4.53)	4.36 (4.24 - 4.49)
4	4.72 (4.59 - 4.86)	4.71 (4.57 - 4.86)
5 (least deprived)	5.02 (4.86 - 5.18)	5.15 (4.98 - 5.32)
**2021 COVID-19 cohort vs 2019 general population**		
1 (most deprived)	1.84 (1.81 - 1.86)	1.86 (1.84 - 1.89)
2	1.93 (1.90 - 1.96)	1.98 (1.94 - 2.01)
3	2.04 (2.00 - 2.07)	2.10 (2.06 - 2.14)
4	2.09 (2.05 - 2.13)	2.15 (2.10 - 2.19)
5 (least deprived)	2.18 (2.14 - 2.23)	2.25 (2.20 - 2.30)
**2021 COVID-19 cohort vs 2021 general population**		
1 (most deprived)	1.77 (1.75 - 1.79)	1.77 (1.74 - 1.79)
2	1.93 (1.90 - 1.96)	1.95 (1.92 - 1.98)
3	2.06 (2.02 - 2.09)	2.09 (2.06 - 2.13)
4	2.09 (2.05 - 2.12)	2.15 (2.11 - 2.19)
5 (least deprived)	2.21 (2.17 - 2.26)	2.30 (2.26 - 2.35)
**2022 COVID-19 cohort vs 2019 general population**		
1 (most deprived)	1.68 (1.66 - 1.70)	1.59 (1.57 - 1.62)
2	1.64 (1.62 - 1.67)	1.57 (1.54 - 1.59)
3	1.62 (1.60 - 1.65)	1.55 (1.52 - 1.58)
4	1.64 (1.62 - 1.67)	1.53 (1.49 - 1.56)
5 (least deprived)	1.62 (1.59 - 1.65)	1.50 (1.47 - 1.54)
**2022 COVID-19 cohort vs 2022 general population**		
1 (most deprived)	1.58 (1.55 - 1.60)	1.55 (1.53 - 1.57)

For peer review only - http://bmjopen.bmj.com/site/about/guidelines.xhtml

2	1.60 (1.58 - 1.62)	1.59 (1.56 - 1.61)
3	1.58 (1.56 - 1.61)	1.57 (1.55 - 1.59)
4	1.61 (1.59 - 1.64)	1.60 (1.58 - 1.63)
5 (least deprived)	1.56 (1.54 - 1.59)	1.56 (1.53 - 1.58)
**2020 hospitalised COVID-19 cohort vs 2019 hospitalised pneumonia cohort**		
1 (most deprived)	0.96 (0.89 - 1.03)	0.76 (0.68 - 0.84)
2	0.9 (0.83 - 0.97)	0.73 (0.65 - 0.82)
3	0.93 (0.85 - 1.01)	0.83 (0.73 - 0.94)
4	0.88 (0.81 - 0.96)	0.84 (0.73 - 0.96)
5 (least deprived)	0.78 (0.70 - 0.86)	0.71 (0.60 - 0.83)
**2021 hospitalised COVID-19 cohort vs 2019 hospitalised pneumonia cohort**		
1 (most deprived)	1.02 (0.97 - 1.09)	0.85 (0.78 - 0.93)
2	0.96 (0.90 - 1.03)	0.80 (0.72 - 0.89)
3	0.96 (0.90 - 1.04)	0.89 (0.79 - 0.99)
4	0.88 (0.81 - 0.96)	0.79 (0.70 - 0.90)
5 (least deprived)	0.82 (0.75 - 0.90)	0.69 (0.60 - 0.79)
**2022 hospitalised COVID-19 cohort vs 2019 hospitalised pneumonia cohort**		
1 (most deprived)	0.71 (0.67 - 0.76)	0.66 (0.60 - 0.72)
2	0.65 (0.61 - 0.70)	0.63 (0.57 - 0.70)
3	0.62 (0.57 - 0.66)	0.63 (0.57 - 0.70)
4	0.60 (0.56 - 0.65)	0.57 (0.51 - 0.64)
5 (least deprived)	0.60 (0.55 - 0.65)	0.59 (0.52 - 0.67)

*Fully adjusted models include age, sex, IMD quintile, region, ethnicity, obesity, smoking status, hypertension, diabetes, chronic respiratory disease, asthma, chronic cardiac disease, lung cancer, haematological cancer, other cancer, chronic liver disease,other neurological disease, organ transplant, asplenia, HIV, permanent immunodeficiency, and rheumatoid arthritis/systemic lupus erythematosus/psoriasis.

For peer review only

Supplementary Table 9. Crude and adjusted hazard ratio of first fit note for all comparisons, stratified by region

Region	Crude HR (95%CI)	Fully adjusted HR (95%CI)
**2020 COVID-19 cohort vs 2019 general population**		
East Midlands	3.13 (3.05 - 3.22)	3.12 (3.04 - 3.21)
East	3.34 (3.24 - 3.45)	3.17 (3.07 - 3.27)
London	3.68 (3.45 - 3.92)	3.24 (3.03 - 3.47)
North East	3.12 (3.00 - 3.24)	3.15 (3.03 - 3.28)
North West	3.20 (3.11 - 3.30)	3.24 (3.14 - 3.33)
South East	3.28 (3.06 - 3.52)	3.14 (2.92 - 3.37)
South West	3.44 (3.29 - 3.61)	3.36 (3.21 - 3.52)
West Midlands	3.29 (3.14 - 3.46)	3.32 (3.16 - 3.49)
Yorkshire & The Humber	3.15 (3.07 - 3.22)	3.16 (3.08 - 3.24)
**2020 COVID-19 cohort vs 2029 general population**		
East Midlands	4.05 (3.94 - 4.16)	3.98 (3.87 - 4.09)
East	4.56 (4.42 - 4.71)	4.28 (4.14 - 4.43)
London	5.22 (4.89 - 5.58)	4.57 (4.26 - 4.89)
North East	3.86 (3.71 - 4.02)	3.86 (3.71 - 4.02)
North West	3.94 (3.82 - 4.06)	3.92 (3.80 - 4.04)
South East	4.34 (4.04 - 4.65)	4.15 (3.86 - 4.46)
South West	4.55 (4.35 - 4.77)	4.40 (4.19 - 4.61)
West Midlands	4.37 (4.16 - 4.59)	4.34 (4.13 - 4.57)
Yorkshire & The Humber	4.02 (3.92 - 4.12)	3.97 (3.88 - 4.07)
**2021 COVID-19 cohort vs 2019 general population**		
East Midlands	2.05 (2.02 - 2.09)	2.06 (2.02 - 2.10)
East	2.01 (1.97 - 2.05)	1.97 (1.93 - 2.01)
London	2.63 (2.53 - 2.74)	2.62 (2.50 - 2.74)
North East	1.96 (1.91 - 2.01)	1.97 (1.91 - 2.03)
North West	1.85 (1.81 - 1.88)	1.87 (1.83 - 1.91)
South East	2.02 (1.94 - 2.10)	1.98 (1.90 - 2.07)
South West	2.03 (1.98 - 2.09)	1.99 (1.93 - 2.04)
West Midlands	2.10 (2.03 - 2.17)	2.18 (2.1 - 2.26)
Yorkshire & The Humber	2.03 (1.99 - 2.06)	2.04 (2.00 - 2.07)
**2021 COVID-19 cohort vs 2021 general population**		
East Midlands	1.95 (1.92 - 1.98)	1.99 (1.96 - 2.02)
East	2.07 (2.03 - 2.11)	2.07 (2.04 - 2.11)

For peer review only - http://bmjopen.bmj.com/site/about/guidelines.xhtml

London	2.69 (2.59 - 2.79)	2.60 (2.50 - 2.70)
North East	1.85 (1.80 - 1.90)	1.89 (1.84 - 1.94)
North West	1.73 (1.69 - 1.76)	1.76 (1.72 - 1.79)
South East	2.02 (1.95 - 2.09)	2.05 (1.98 - 2.12)
South West	2.02 (1.97 - 2.06)	2.06 (2.02 - 2.11)
West Midlands	2.04 (1.98 - 2.11)	2.08 (2.01 - 2.14)
Yorkshire & The Humber	1.92 (1.89 - 1.95)	1.95 (1.92 - 1.99)
**2022 COVID-19 cohort vs 2019 general population**		
East Midlands	1.71 (1.68 - 1.73)	1.58 (1.55 - 1.61)
East	1.61 (1.58 - 1.64)	1.50 (1.47 - 1.53)
London	1.91 (1.84 - 1.98)	1.96 (1.88 - 2.05)
North East	1.68 (1.63 - 1.73)	1.56 (1.51 - 1.61)
North West	1.63 (1.60 - 1.66)	1.49 (1.46 - 1.53)
South East	1.67 (1.61 - 1.73)	1.53 (1.47 - 1.59)
South West	1.66 (1.62 - 1.69)	1.47 (1.44 - 1.51)
West Midlands	1.80 (1.75 - 1.86)	1.74 (1.68 - 1.81)
Yorkshire & The Humber	1.65 (1.63 - 1.68)	1.56 (1.53 - 1.59)
**2022 COVID-19 cohort vs 2022 general population**		
East Midlands	1.55 (1.53 - 1.58)	1.57 (1.55 - 1.60)
East	1.55 (1.53 - 1.58)	1.57 (1.55 - 1.59)
London	1.79 (1.74 - 1.86)	1.90 (1.83 - 1.96)
North East	1.50 (1.46 - 1.54)	1.53 (1.49 - 1.57)
North West	1.51 (1.48 - 1.54)	1.51 (1.48 - 1.54)
South East	1.54 (1.50 - 1.58)	1.54 (1.50 - 1.58)
South West	1.54 (1.52 - 1.57)	1.54 (1.52 - 1.57)
West Midlands	1.62 (1.57 - 1.67)	1.65 (1.60 - 1.71)
Yorkshire & The Humber	1.53 (1.51 - 1.56)	1.58 (1.55 - 1.61)
**2020 hospitalised COVID-19 cohort vs 2019 hospitalised pneumonia cohort**		
East Midlands	0.83 (0.77 - 0.91)	0.69 (0.61 - 0.78)
East	0.78 (0.67 - 0.91)	0.74 (0.65 - 0.84)
London	0.94 (0.82 - 1.09)	0.65 (0.51 - 0.83)
North East	1.06 (0.95 - 1.18)	0.81 (0.65 - 1.02)
North West	0.75 (0.63 - 0.90)	0.89 (0.76 - 1.04)
South East	1.10 (0.98 - 1.23)	0.58 (0.45 - 0.76)

For peer review only - http://bmjopen.bmj.com/site/about/guidelines.xhtml

South West	0.88 (0.75 - 1.02)	1.08 (0.91 - 1.28)
West Midlands	0.96 (0.88 - 1.05)	0.74 (0.59 - 0.93)
Yorkshire & The Humber	0.83 (0.77 - 0.91)	0.78 (0.69 - 0.90)
**2021 hospitalised COVID-19 cohort vs 2019 hospitalised pneumonia cohort**		
East Midlands	0.91 (0.85 - 0.98)	0.78 (0.69 - 0.87)
East	0.92 (0.85 - 0.99)	0.79 (0.70 - 0.88)
London	0.86 (0.74 - 1.00)	0.80 (0.65 - 1.00)
North East	0.94 (0.83 - 1.08)	0.83 (0.68 - 1.03)
North West	0.98 (0.89 - 1.09)	0.81 (0.70 - 0.95)
South East	0.90 (0.78 - 1.04)	0.79 (0.63 - 0.99)
South West	0.99 (0.90 - 1.09)	0.82 (0.71 - 0.95)
West Midlands	0.96 (0.84 - 1.11)	0.92 (0.74 - 1.15)
Yorkshire & The Humber	0.98 (0.91 - 1.06)	0.86 (0.77 - 0.97)
**2022 hospitalised COVID-19 cohort vs 2019 hospitalised pneumonia cohort**		
East Midlands	0.68 (0.63 - 0.73)	0.68 (0.61 - 0.75)
East	0.60 (0.56 - 0.65)	0.60 (0.54 - 0.66)
London	0.61 (0.52 - 0.71)	0.72 (0.58 - 0.90)
North East	0.59 (0.52 - 0.68)	0.58 (0.48 - 0.70)
North West	0.69 (0.63 - 0.77)	0.61 (0.53 - 0.71)
South East	0.58 (0.51 - 0.66)	0.56 (0.46 - 0.67)
South West	0.63 (0.58 - 0.69)	0.58 (0.51 - 0.67)
West Midlands	0.74 (0.64 - 0.86)	0.72 (0.58 - 0.88)
Yorkshire & The Humber	0.69 (0.64 - 0.75)	0.61 (0.54 - 0.68)

*Fully adjusted models include age, sex, IMD quintile, region, ethnicity, obesity, smoking status, hypertension, diabetes, chronic respiratory disease, asthma, chronic cardiac disease, lung cancer, haematological cancer, other cancer, chronic liver disease, other neurological disease, organ transplant, asplenia, HIV, permanent immunodeficiency, and rheumatoid arthritis/systemic lupus erythematosus/psoriasis.

For peer review only

Supplementary Figure 3. Adjusted hazard ratio of first fit note comparing hospitalised COVID-19 cohorts to people hospitalised with pneumonia in 2019, stratified by demographic categories and year

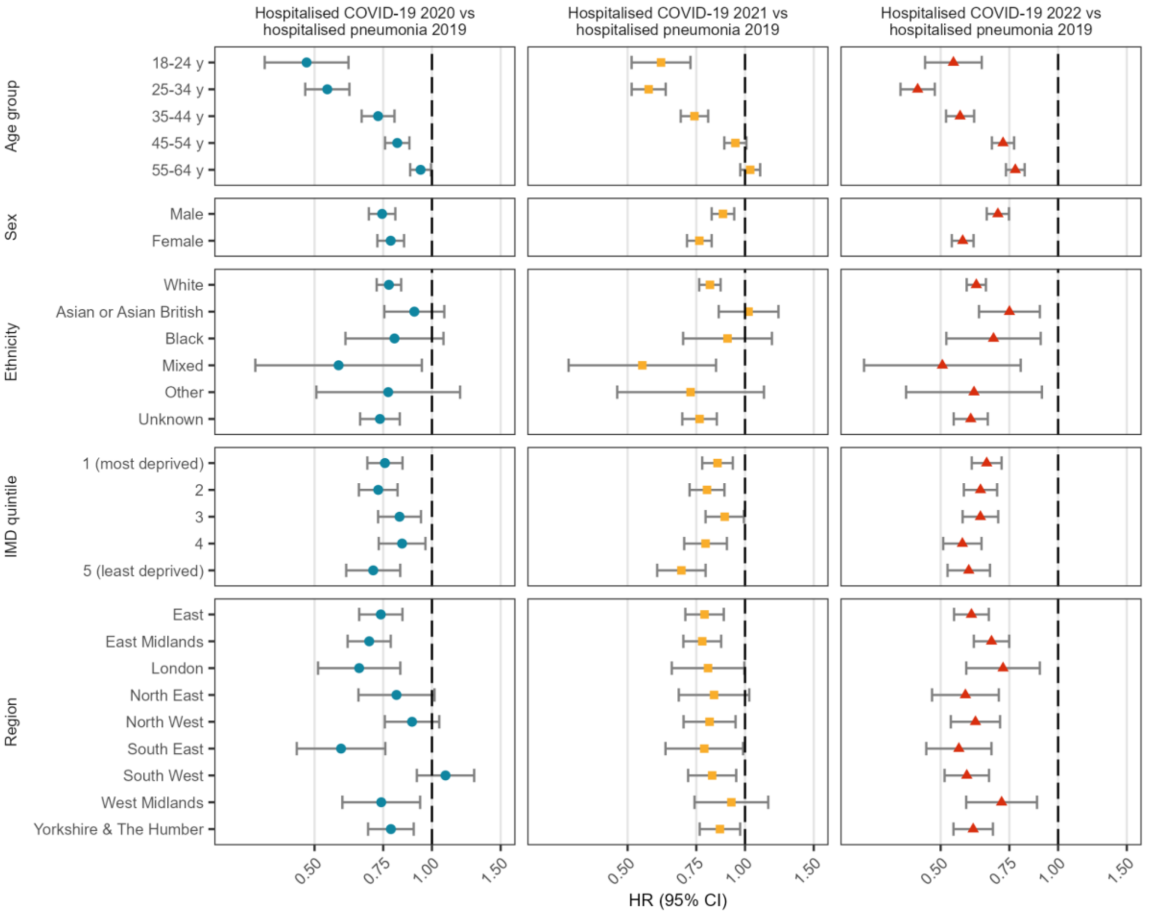

*All models are adjusted for age, sex, ethnicity, IMD quintile and region, excluding the stratification variable. Models are additionally adjusted for obesity, smoking status, hypertension, diabetes, chronic respiratory disease, asthma, chronic cardiac disease, lung cancer, haematological cancer, other cancer, chronic liver disease, other neurological disease, organ transplant, asplenia, HIV, permanent immunodeficiency, and rheumatoid arthritis/systemic lupus erythematosus/psoriasis

For peer review only - http://bmjopen.bmj.com/site/about/guidelines.xhtml

The RECORD statement – checklist of items, extended from the STROBE statement, that should be reported in observational studies using routinely collected health data.

	Item No.	STROBE items	Location in manuscript where items are reported	RECORD items	Location in manuscript where items are reported
**Title and abstract**					
	1	(a) Indicate the study's design with a commonly used term in the title or the abstract (b) Provide in the abstract an informative and balanced summary of what was done and what was found		RECORD 1.1: The type of data used should be specified in the title or abstract. When possible, the name of the databases used should be included.<br><br>RECORD 1.2: If applicable, the geographic region and timeframe within which the study took place should be reported in the title or abstract.<br><br>RECORD 1.3: If linkage between databases was conducted for the study, this should be clearly stated in the title or abstract.	1.1<br>Title<br>Page 6 (Data Source and Data Sharing)<br><br>1.2 Abstract
**Introduction**					
Background rationale	2	Explain the scientific background and rationale for the investigation being reported			Background
Objectives	3	State specific objectives, including any prespecified hypotheses			Last paragraph of Background
**Methods**					
Study Design	4	Present key elements of study design early in the paper			Title<br>Methods - Study design
Setting	5	Describe the setting, locations, and relevant dates, including periods of recruitment, exposure, follow-up, and data collection			Methods - Study population)<br>Methods - Study measures

For peer review only

Participants	6	*(a) Cohort study* - Give the eligibility criteria, and the sources and methods of selection of participants. Describe methods of follow-up<br>*Case-control study* - Give the eligibility criteria, and the sources and methods of case ascertainment and control selection. Give the rationale for the choice of cases and controls<br>*Cross-sectional study* - Give the eligibility criteria, and the sources and methods of selection of participants<br><br>*(b) Cohort study* - For matched studies, give matching criteria and number of exposed and unexposed<br>*Case-control study* - For matched studies, give matching criteria and the number of controls per case		RECORD 6.1: The methods of study population selection (such as codes or algorithms used to identify subjects) should be listed in detail. If this is not possible, an explanation should be provided.<br><br>RECORD 6.2: Any validation studies of the codes or algorithms used to select the population should be referenced. If validation was conducted for this study and not published elsewhere, detailed methods and results should be provided.<br><br>RECORD 6.3: If the study involved linkage of databases, consider use of a flow diagram or other graphical display to demonstrate the data linkage process, including the number of individuals with linked data at each stage.	6.1 A link to all code and codelists used in the study is provided in the Methods<br><br>6.2 N/A<br><br>6.3 N/A
Variables	7	Clearly define all outcomes, exposures, predictors, potential confounders, and effect modifiers. Give diagnostic criteria, if applicable.		RECORD 7.1: A complete list of codes and algorithms used to classify exposures, outcomes, confounders, and effect modifiers should be provided. If these cannot be reported, an explanation should be provided.	A link to all code and codelists used in the study is provided in the Methods
Data sources/ measurement	8	For each variable of interest, give sources of data and details of methods of assessment (measurement).<br>Describe comparability of assessment methods if there is more than one group			Methods - Study measures;<br>A link to all code and codelists used in the study is provided in the Methods

For peer review only

Bias	9	Describe any efforts to address potential sources of bias			Methods – Study population<br>Methods - Cox regression<br>Discussion – Strengths and Weaknesses
Study size	10	Explain how the study size was arrived at			Methods - Study population
Quantitative variables	11	Explain how quantitative variables were handled in the analyses. If applicable, describe which groupings were chosen, and why			Methods - Cox regression
Statistical methods	12	(a) Describe all statistical methods, including those used to control for confounding<br>(b) Describe any methods used to examine subgroups and interactions<br>(c) Explain how missing data were addressed<br>(d) *Cohort study* - If applicable, explain how loss to follow-up was addressed<br>*Case-control study* - If applicable, explain how matching of cases and controls was addressed<br>*Cross-sectional study* - If applicable, describe analytical methods taking account of sampling strategy<br>(e) Describe any sensitivity analyses			Methods - Statistical methods
Data access and cleaning methods		..		RECORD 12.1: Authors should describe the extent to which the investigators had access to the database	Data access and verification

For peer review only

				population used to create the study population.<br><br>RECORD 12.2: Authors should provide information on the data cleaning methods used in the study.	
Linkage		..		RECORD 12.3: State whether the study included person-level, institutional-level, or other data linkage across two or more databases. The methods of linkage and methods of linkage quality evaluation should be provided.	Methods - Data source
**Results**					
Participants	13	(a) Report the numbers of individuals at each stage of the study (*e.g.*, numbers potentially eligible, examined for eligibility, confirmed eligible, included in the study, completing follow-up, and analysed)<br>(b) Give reasons for non-participation at each stage.<br>(c) Consider use of a flow diagram		RECORD 13.1: Describe in detail the selection of the persons included in the study (*i.e.*, study population selection) including filtering based on data quality, data availability and linkage. The selection of included persons can be described in the text and/or by means of the study flow diagram.	Results - Study population description
Descriptive data	14	(a) Give characteristics of study participants (*e.g.*, demographic, clinical, social) and information on exposures and potential confounders<br>(b) Indicate the number of participants with missing data for each variable of interest<br>(c) *Cohort study* - summarise follow-up time (*e.g.*, average and total amount)			Results - Study population description)<br>Table 1<br>Supplementary Table 1

For peer review only

Outcome data	15	*Cohort study* - Report numbers of outcome events or summary measures over time<br>*Case-control study* - Report numbers in each exposure category, or summary measures of exposure<br>*Cross-sectional study* - Report numbers of outcome events or summary measures			Results - Overall fit note rates<br>Supplementary Table 2<br>Supplementary Table 3
Main results	16	(a) Give unadjusted estimates and, if applicable, confounder-adjusted estimates and their precision (e.g., 95% confidence interval). Make clear which confounders were adjusted for and why they were included<br>(b) Report category boundaries when continuous variables were categorized<br>(c) If relevant, consider translating estimates of relative risk into absolute risk for a meaningful time period			Table 2<br>Supplementary Table 3<br>Figure 1
Other analyses	17	Report other analyses done—e.g., analyses of subgroups and interactions, and sensitivity analyses			
**Discussion**					
Key results	18	Summarise key results with reference to study objectives			Discussion - Summary
Limitations	19	Discuss limitations of the study, taking into account sources of potential bias or imprecision. Discuss both direction and magnitude of any potential bias		RECORD 19.1: Discuss the implications of using data that were not created or collected to answer the specific research question(s). Include discussion of misclassification bias, unmeasured confounding, missing data, and changing eligibility over	Discussion - Strengths and weaknesses

For peer review only

				time, as they pertain to the study being reported.	
Interpretation	20	Give a cautious overall interpretation of results considering objectives, limitations, multiplicity of analyses, results from similar studies, and other relevant evidence			Discussion - Policy implications and interpretation
Generalisability	21	Discuss the generalisability (external validity) of the study results			Discussion - Strengths and weaknesses
**Other Information**					
Funding	22	Give the source of funding and the role of the funders for the present study and, if applicable, for the original study on which the present article is based			Methods – Role of the funding source Funding
Accessibility of protocol, raw data, and programming code		..		RECORD 22.1: Authors should provide information on how to access any supplemental information such as the study protocol, raw data, or programming code.	A link to the study’s Github repo, including protocol, code and codelists, is provided in the Methods

*Reference: Benchimol EI, Smeeth L, Guttmann A, Harron K, Moher D, Petersen I, Sørensen HT, von Elm E, Langan SM, the RECORD Working Committee. The REporting of studies Conducted using Observational Routinely-collected health Data (RECORD) Statement. *PLoS Medicine* 2015; in press.

*Checklist is protected under Creative Commons Attribution (CC BY) license.

BMJ Open

Changes in sick notes associated with COVID-19 from 2020 to 2022: A cohort study in 24 million primary care patients in OpenSAFELY-TPP

Journal:	*BMJ Open*
Manuscript ID	bmjopen-2023-080600.R1
Article Type:	Original research
Date Submitted by the Author:	20-May-2024

Complete List of Authors:	Schaffer, Andrea; University of Oxford, Nuffield Department of Primary Care Health Sciences<br>Park, Robin Y; University of Oxford, Nuffield Department of Primary Care Health Sciences<br>Tazare, John; London School of Hygiene & Tropical Medicine<br>Bhaskaran, Krishnan; LSHTM, NCDE<br>MacKenna, Brian; University of Oxford, Nuffield Department of Primary Care Health Sciences<br>Denaxas, S; UCL, Institute of Health Informatics; NIHR University College London Hospitals Biomedical Research Centre<br>Dillingham, Iain; University of Oxford, Nuffield Department of Primary Care Health Sciences<br>Bacon, Seb; University of Oxford, Nuffield Department of Primary Care Health Sciences<br>Mehrkar, Amir; University of Oxford Nuffield Department of Primary Care Health Sciences<br>Bates, Christopher; TPP<br>Goldacre, Ben; University of Oxford, Primary Care Health Sciences<br>Greaves, Felix; National Institute for Health and Care Excellence; Imperial College London Department of Primary Care and Public Health<br>Macleod, John; NIHR Applied Research Collaboration West<br>Consortium, The OpenSAFELY ; Oxford University<br>Collaborative, National Core Studies; University of Oxford Nuffield Department of Primary Care Health Sciences, Bennett Institute for Applied Data Science<br>Tomlinson, Laurie; London School of Hygiene and Tropical Medicine, Epidemiology and Population Health<br>Walker, Alex; University of Oxford, Nuffield Department of Primary Care Health Sciences

**Primary Subject Heading**:	Epidemiology
Secondary Subject Heading:	General practice / Family practice
Keywords:	COVID-19, EPIDEMIOLOGY, PUBLIC HEALTH, Primary Care < Primary Health Care, SARS-CoV-2 Infection

SCHOLARONE™
Manuscripts

I, the Submitting Author has the right to grant and does grant on behalf of all authors of the Work (as defined in the below author licence), an exclusive licence and/or a non-exclusive licence for contributions from authors who are: i) UK Crown employees; ii) where BMJ has agreed a CC-BY licence shall apply, and/or iii) in accordance with the terms applicable for US Federal Government officers or employees acting as part of their official duties; on a worldwide, perpetual, irrevocable, royalty-free basis to BMJ Publishing Group Ltd ("BMJ") its licensees and where the relevant Journal is co-owned by BMJ to the co-owners of the Journal, to publish the Work in this journal and any other BMJ products and to exploit all rights, as set out in our licence.

The Submitting Author accepts and understands that any supply made under these terms is made by BMJ to the Submitting Author unless you are acting as an employee on behalf of your employer or a postgraduate student of an affiliated institution which is paying any applicable article publishing charge ("APC") for Open Access articles. Where the Submitting Author wishes to make the Work available on an Open Access basis (and intends to pay the relevant APC), the terms of reuse of such Open Access shall be governed by a Creative Commons licence – details of these licences and which Creative Commons licence will apply to this Work are set out in our licence referred to above.

Other than as permitted in any relevant BMJ Author's Self Archiving Policies, I confirm this Work has not been accepted for publication elsewhere, is not being considered for publication elsewhere and does not duplicate material already published. I confirm all authors consent to publication of this Work and authorise the granting of this licence.

For peer review only

Changes in sick notes associated with COVID-19 from 2020 to 2022: A cohort study in 24 million primary care patients in OpenSAFELY-TPP

Andrea L Schaffer[1*], Robin Y Park[1], John Tazare[2], Krishnan Bhaskaran[2], Brian MacKenna[1], Spiros Denaxas[3,4,5], Iain Dillingham[1], Sebastian CJ Bacon[1], Amir Mehrkar[1], Chris Bates[6], Ben Goldacre[1], Felix Greaves[7,8], John Macleod[9], The OpenSAFELY Collaborative^, National Core Studies Collaborative^, Laurie A Tomlinson[2], Alex J Walker[1],

[1] Bennett Institute for Applied Data Science, Nuffield Department of Primary Care Health Sciences, University of Oxford, Oxford, UK

[2] London School of Hygiene and Tropical Medicine, London, UK

[3] Institute of Health Informatics, University College London, London, UK

[4] University College London Hospitals Biomedical Research Centre, London, UK

[5] BHF Data Science Centre, Health Data Research UK, London, UK

[6] TPP, TPP House, Leeds, UK

[7] Department of Primary Care and Public Health, Imperial College London, London, UK

[8] National Institute for Health and Care Excellence, London, UK

[9] NIHR Applied Research Collaboration (ARC) West, Bristol, UK

*Corresponding author:

Dr Andrea Schaffer andrea.schaffer@phc.ox.ac.uk

For peer review only

Abstract

Objectives: Long-term sickness absence from employment has negative consequences for the economy and can lead to widened health inequalities. Sick notes (also called “fit notes”) are issued by general practitioners (GPs) when a person can’t work for health reasons for more than seven days. We quantified the sick note rate in people with evidence of COVID-19 in 2020, 2021, and 2022, as an indication of the burden for people recovering from COVID-19.

Design: Cohort study.

Setting: With NHS England approval, we used routine clinical data (primary care, hospital and COVID-19 testing records) within the OpenSAFELY-TPP database

Participants: People 18-64 years with a recorded positive test or diagnosis of COVID-19 in 2020 (n=365,421), 2021 (n=1,206,555) or 2022 (n=1,321,313); general population matched on age, sex and region in 2019 (n=3,140,326), 2020 (n=3,439,534), 2021 (n=4,571,469), and 2022 (n=4,818,870); people hospitalised with pneumonia in 2019 (n=29,673).

Primary outcome measure: Receipt of a sick note in primary care.

Results: Among people with a positive SARS-CoV-2 test or COVID-19 diagnosis, the sick note rate was 4.88 per 100 person-months (95%CI 4.83-4.93) in 2020, 2.66 (95%CI 2.64-2.67) in 2021, and 1.73 (95%CI 1.72-1.73) in 2022. Compared with the age, sex and region-matched general population, the adjusted hazard ratio (HR) for receipt of a sick note over the entire follow-up period (up to 10 months) was 4.07 (95%CI 4.02-4.12) in 2020 decreasing to 1.57 (95%CI 1.56-1.58) in 2022. The HR was highest in the first 30 days post-diagnosis in all years. Among people hospitalised with COVID-19, after adjustment the sick note rate was lower than in people hospitalised with pneumonia.

Conclusions: Given the under-recording of post-acute COVID-19-related symptoms, these findings contribute a valuable perspective on the long-term effects of COVID-19. Despite likely underestimation of the sick note rate, sick notes were issued more frequently to people with COVID-19 compared to those without, even in an era when most people are vaccinated. Most sick notes occurred in the first 30 days post-diagnosis, but the increased risk several months post-diagnosis may provide further evidence of the long-term impact.

Keywords

sars-cov-2, covid-19, long covid, fit notes, sick notes, sick leave, primary care, epidemiology, public health

For peer review only

Strengths and limitations

- Our data come from a large, representative sample of 40% of primary care patients in England.
- To better understand sick note patterns, we identified COVID-19 cohorts from three different pandemic time periods, with both contemporary and historical comparators, to account for changes in preventive measures and disruptions to working patterns that would have impacted on receipt of sick notes over time.
- We relied on three different methods for identifying COVID-19 cases (positive PCR or LFT test, primary care diagnosis, hospitalisation), but there is still bias in who gets tested or seeks care for COVID-19 which will have changed as the pandemic evolved. It is unclear how this would correlate with the likelihood of requesting or requiring a sick note.
- We could not identify whether people were participating in the workforce and included everyone of working age. It is therefore likely that the true absolute sick note rate is higher than estimated.

For peer review only

Background

In primary care, a doctor may issue a sick note (commonly referred to as a “fit note” in the UK) after the first seven days of sickness absence if the doctor assesses that the patient’s health affects their fitness for work. In 2021-22, over 11 million sick notes were issued in England.(1) Long-term sickness absence from employment has negative consequences for the broader economy and can lead to widened health inequalities, financial insecurity, and reduced social participation.(2) Improving the health and productivity of the population and reducing welfare benefit claims are important policy objectives.(3,4)

The Office of National Statistics (ONS) estimated that 82% of the English population had been infected with SARS-CoV-2 by November 2022.(5) Additionally, an estimated 1.9 million people self-reported as experiencing long COVID symptoms in the UK in the four weeks prior to March 2023, with two-thirds experiencing symptoms for at least one year.(6) The prevalence of long-term symptoms was greatest in people aged 35-69 years, women, and people living in more deprived areas.(6)) Previous UK research has also shown that receipt of sick notes varies by demographics, with women and people in manual and service occupations more likely to be issued a sick note.(4) To date, there has been limited research on sick notes issued to patients recovering from COVID-19 in England.

The pandemic has evolved over time, both in the number of cases, disease severity, and demographic groups most affected.(7) Given the risk of long-term symptoms following COVID-19 (“long COVID”), it is of public health interest to quantify the impact of infection and recovery on the workforce and how that has changed over the pandemic. However, long COVID is heterogeneous and difficult to define.(8,9) Furthermore, coding of long COVID in primary care is very low,(10) and cannot be relied on to identify people suffering from persistent symptoms. Therefore, our objectives were to: 1) describe the demographic and clinical characteristics of people given a sick note following a documented SARS-CoV-2 infection or COVID-19 diagnosis; 2) determine how the sick note rate varies over time post-diagnosis; and 3) quantify the difference in sick note rate in people with SARS-CoV-2 infection or COVID-19 diagnosis compared with the general population and people hospitalised with pneumonia.

For peer review only

Methods

Study design

We conducted an observational cohort study using general practice primary care electronic health record (EHR) data from primary care practices in England.

Data source and data sharing

We used primary care data from approximately 40% of the English population currently registered with GP surgeries using TPP SystmOne software. All data were linked, stored and analysed securely using the OpenSAFELY platform, https://www.opensafely.org/, as part of the NHS England OpenSAFELY COVID-19 service. Data include pseudonymised data such as coded diagnoses, medications and physiological parameters. No free text data are included. All code is shared openly for review and re-use under MIT open license (https://github.com/opensafely/long-covid-sick-notes). Similarly, pseudonymised datasets including ONS registered deaths, hospital episode statistics (HES), and Second Generation Surveillance System (SGSS) COVID-19 test results are securely provided to TPP and linked to primary care data. Detailed pseudonymised patient data are potentially re-identifiable and not shared.

Study population

We included adults 18-64 years registered with one GP for at least one year prior to their index date with information on age, sex, index of multiple deprivation (IMD) and the Sustainability and Transformation Partnership region (STP, an NHS administrative region). This age range was selected to represent people most likely to be in the workforce. From this source population, we identified three cohorts with recorded SARS-CoV-2 infection between 1 February and 30 November in each of 2020, 2021 and 2022 (hereafter referred to as the "COVID-19 cohorts"). We used the period February to November (instead of full years) to allow a wash-out period between years.

The COVID-19 cohorts were identified through three routes: having a recorded positive test for SARS-CoV-2 based on SGSS data, which captures both polymerase chain reaction (PCR) and lateral flow tests (LFT); having a probable diagnostic code for COVID-19 in primary care records; or being hospitalised with a primary or secondary diagnosis for COVID-19 (ICD-10 codes U07.1 or U07.2) identified from HES. The index date was the earliest of these events. As comparators, we identified contemporary and one historical (2019) general population cohort who were frequency-matched on age, sex and STP to the COVID-19 cohorts. The 2019 general population cohort was matched with the 2020 COVID-19 cohort. For the comparator cohorts, the index date was randomly assigned and randomly distributed over the study period.

We also performed a secondary analysis among the subset of people hospitalised with COVID-19. Here, the index date was the date of admission. We compared these individuals with people hospitalised with pneumonia between 1 February 2019 and 30 November 2019. We chose pneumonia as a comparator to provide context, as it is a common, serious respiratory disease with which clinicians and policy makers will be familiar. The pneumonia cohort was identified using the following ICD-10 codes in any diagnosis position:

For peer review only - http://bmjopen.bmj.com/site/about/guidelines.xhtml

B01.2, B05.2, B20.6, B25.0, J10-J18, J85.1, U04. No contemporary pneumonia comparator cohort was included due to potential for misclassification with COVID-19. People could be included in multiple cohorts if they met the inclusion criteria. A figure depicting the 11 cohorts is in **Figure 1**.

Study measures

In England, employees can self-certify for the first seven days of sickness; after this, they can receive a Statement of Fitness for Work (also called a fit note or sick note) from their general practitioner (GP) if they determine that the patient's health affects their fitness for work. Unemployed patients can also receive a sick note to support claims for health-related benefits. Sick notes can initially be issued for up to 3 months, and then periodically reviewed if needed. During this time, people can receive statutory sick pay.(11)

We identified the first recorded sick note over patient follow-up. Sick notes were identified using Clinical Terms Version 3 (CTV3) codes. Sick notes issued due to COVID-19 did not differ from sick notes issues at other times or for other health conditions. People who were required to isolate beyond seven days due to having or living with someone with symptoms of COVID-19 would be issued an isolation note, rather than a sick note. Isolation notes did not require contact with a GP and are not counted. Follow-up was censored at the end of the study period (30 November), death, or deregistration from their GP practice. The comparator cohorts were also censored if they had a recorded positive SARS-CoV-2 test or COVID-19 diagnosis.

We characterised the demographic and clinical characteristics of patients who received a sick note. Measured demographic variables included age (18-24, 25-34, 35-44, 45-54, 55-64 years), sex (male, female), GP practice region (East Midlands, East, London, North East, North West, South East, South West, West Midlands, Yorkshire and The Humber), ethnicity (White, Asian and British Asian, Black, Other, Unknown), and Indices of Multiple Deprivation (IMD) quintiles.

Clinical variables included indicators for pre-existing conditions that may impact the severity of COVID-19, specifically: asthma, cancer (haematological, lung, other), chronic cardiac disease, chronic liver disease, chronic respiratory disease (excluding asthma), diabetes, asplenia, human immunodeficiency virus (HIV) infection, hypertension, obesity, organ transplant, neurological conditions, other permanent immunodeficiency (excluding HIV), smoking status (current, former or never), and autoimmune conditions (rheumatoid arthritis, systemic lupus erythematosus (SLE), psoriasis). Obesity and smoking status were identified using the most recent recorded information, while the remainder were identified at any time prior to the index date.

Statistical methods

For each of the eleven cohorts, we reported the first sick note rate per 100 person months overall and stratified by demographics (age, sex, ethnicity, region, IMD quintile). To prevent disclosure, all counts in this manuscript are rounded to the nearest seven.

For peer review only - http://bmjopen.bmj.com/site/about/guidelines.xhtml

We used Cox regression models to estimate hazard ratios (HRs) and 95% confidence intervals (CIs) comparing the first sick note rate between the COVID-19 cohorts and the comparator cohorts. We investigated crude univariable and two covariate-adjusted models: (1) adjusted for age (cubic splines with 4 knots) and sex; (2) adjusted for demographics and all clinical characteristics described above. While the same people can contribute person-time to multiple exposure groups, these periods are non-overlapping, so we applied robust standard errors. To determine if the relative differences in sick note occurrence changed over time, we estimated crude and adjusted HRs and 95%CIs both over the entire follow-up period (up to 10 months) and censoring follow-up at 30, 90, and 150 days, and examined whether HRs changed according to the duration of follow-up. We chose this approach instead of estimating period-specific HRs in order to avoid selection bias due to depletion of susceptibles.(12) Similarly, to explore the impact within demographic categories, we estimated crude and adjusted HRs and 95%CIs stratified by age group, sex, ethnicity, IMD quintile, and region.

Software and reproducibility

Data management was performed using Python 3.8, with analyses carried out using Stata 16.1, R 4.3.0, and Python. Code for data management and analysis as well as all codelists used in this study are available online: https://github.com/opensafely/long-covid-sick-notes.

Patient and Public Involvement

We have involved patients and the public in various ways: we developed a public website that provides a detailed description of the platform in language suitable for a lay audience (https://opensafely.org); we have participated in two citizen juries exploring public trust in OpenSAFELY; we co-developed an explainer video; we have patient representation who are experts by experience on our OpenSAFELY Oversight Board; we have partnered with Understanding Patient Data to produce lay explainers on the importance of large datasets for research; we have presented at various online public engagement events to key communities; and more. To ensure the patient voice is represented, we are working closely to decide on language choices with appropriate medical research charities.

Results

Study population

We identified 365,421 people with a recorded positive SARS-CoV-2 test or COVID-19 diagnosis in 2020, of which 22,015 (6.0%) were hospitalised; 1,206,555 people in 2021 (30,205 [2.5%] hospitalised); and 1,321,313 people in 2022 (34,692 [2.6%] hospitalised) (**Table 1**). For comparison with COVID-19 patients who were hospitalised, we identified 29,673 patients hospitalised with pneumonia in 2019. Most people in the COVID-19 cohorts were identified via SGSS testing (88.8% in 2020, 97.8% in 2021 and 96.5% in 2022) (**Supplementary Figure 1**). Compared with earlier years, the 2022 COVID-19 cohort was older and more likely to be female, White ethnicity, and in less deprived IMD quintiles (**Table 1**, **Supplementary Table 1**). Hospitalised COVID-19 cohorts tended to be older, and were more likely to be male, non-White ethnicity, and in the most deprived IMD quintile compared

For peer review only - http://bmjopen.bmj.com/site/about/guidelines.xhtml

with the overall COVID-19 cohorts. When compared with the pneumonia cohort, hospitalised COVID-19 patients were more likely to be obese, less likely to be current smokers, and had lower rates of many chronic health conditions (**Supplementary Table 2**).

Overall sick note rates

A total of 34,377 (9.4%), 102,949 (8.5%) and 152,859 (11.6%) of people in the COVID-19 cohorts were issued sick notes during follow-up in 2020, 2021 and 2022 (**Supplementary Table 3**). However, after taking into account differences in follow-up time (**Supplementary Figure 2**), the sick note rate among the COVID-19 cohorts decreased over time, from 4.88 per 100 person-months in 2020 (95%CI 4.83-4.93), to 2.66 (95%CI 2.64-2.67) in 2021 and 1.73 (95%CI 1.72-1.73) in 2022 (**Table 2**). The sick note rate was higher in the hospitalised cohorts: 6.78 per 100 person-months (95%CI 6.59-6.98) in 2020, 7.19 (95%CI 7.03 - 7.36) in 2021 and 4.13 (95%CI 4.03-4.22) in 2022. In contrast, the sick note rate in the pneumonia cohort was 7.17 (95%CI 7.01-7.34) (**Supplementary Table 4**).

Sick note rate by demographics

Among people in the COVID-19 cohorts, the sick note rate was higher in 2020 and lower in 2022 for all demographic groups except people 18-24 years (**Table 2**). Generally, the sick note rate was higher in people aged ≥45 years, women, people of Asian or Asian British ethnicity, and lower in people 18-24 years and living in London. The sick note rate increased with greater deprivation as defined by the IMD quintile. In most cases these patterns reflected those in the general population. A slightly different pattern was observed for the hospitalised cohorts (**Supplementary Table 4**). People 45-54 years were most likely to receive a sick note, which was older than the pneumonia cohort. Among hospitalised patients the relationship between sex and receiving a sick note differed by year, with women more likely to receive a sick note in 2020 and men more likely in 2021 and 2022. A linear relationship between lesser deprivation and a higher sick note rate was seen for the pneumonia cohort, but not the hospitalised COVID-19 cohorts.

Cox regression by follow-up period

Most people in the COVID-19 cohorts who were issued a first sick note received it in the first 30 days post-index date (**Table 3**). The fully adjusted HR representing the average effect over the entire study period was highest comparing the 2020 COVID-19 cohort to the 2020 general population (4.07, 95%CI 4.02-4.12) and lowest comparing the 2022 COVID-19 cohort to the 2022 general population (1.57, 95%CI 1.56-1.58) (**Table 3**). The crude and age-sex adjusted HRs are in **Supplementary Table 5**. For all comparisons the HR was greatest in first 30 days from the index date, with fully adjusted HRs ranging from 5.94 (95%CI 5.85-6.03) comparing the 2020 COVID-19 cohort to the 2020 general population to 2.37 (95%CI 2.34-2.39) comparing the 2022 COVID-19 cohort to the 2022 general population. With longer follow-up periods, the HR attenuated but remained high. For the hospitalised cohorts, the overall sick note rate was lower in the COVID-19 cohorts than the pneumonia cohort with the highest rates in the first 30 days. The average HR over all follow-up time ranged from 0.62 (95%CI 0.59-0.65) for the 2022 COVID-19 cohort to 0.81 (95%CI

For peer review only - http://bmjopen.bmj.com/site/about/guidelines.xhtml

0.77-0.86) for the 2021 cohort (**Supplementary Table 6)**. The HR was relatively stable regardless of follow-up period used.

Cox regression stratified by demographic categories

For all demographics groups, the HR was greatest in 2020 when compared with either a contemporary or 2019 historical comparator (**Figure 2, Supplementary Tables 7-11**). In 2020, large relative increases in sick note rate were observed for people ≥35 years, women, and people of Black or Other ethnicity. The HR was also higher in people in less deprived IMD quintiles; when comparing the 2020 COVID-19 cohort to the 2020 general population, the fully adjusted HR ranged from 3.41 (95%CI 3.33-3.48) in the most deprived quintile to 5.15 (95%CI 4.98-5.32) in the least deprived quintile. Most HRs were lower in 2021 compared with 2020, and more so in 2022. The one exception was people 18-24 years, where the HR compared with the general population was 1.47 (95%CI 1.43-1.51) in 2022, compared with 1.24 (95%CI 1.22-1.27) in 2021. Other patterns that differed in 2022 compared with other years was that no meaningful variation by IMD quintile was observed, and a higher HR was seen in men compared with women. The findings were similar comparing the COVID-19 cohorts with the 2019 comparator. Among the hospitalised cohorts, the HRs comparing them with the pneumonia cohort were similar in 2020 and 2021, but lower in 2022 (**Supplementary Figure 3, Supplementary Tables 7-11**). A lower sick note rate in the COVID-19 cohorts compared with the pneumonia cohort was observed for most demographic subgroups.

For peer review only

Discussion

Summary

We found that people with a recorded positive SARS-CoV-2 test or COVID-19 diagnosis had a higher sick note rate than the general population, even after adjusting for demographics and a wide range of clinical characteristics. This increase was greatest in 2020 but continued even into 2022 despite the introduction of vaccines and other effective outpatient COVID-19 treatments. The sick note rate was highest in the first 30 days post-diagnosis, but an increased risk persisted over all follow-up in all years. These findings provide further evidence for the long-term health and economic impact of COVID-19. In contrast, people hospitalised with COVID-19 were less likely to be issued a sick note than people with pneumonia, especially in 2022.

Strengths and weaknesses

Our data come from a representative sample of 40% of the English population.(13) To better understand sick note patterns, we used COVID-19 cohorts from three different pandemic time periods, with both contemporary and historical comparators, to account for changes in preventive measures (e.g. lockdowns, vaccines) and other disruptions to working patterns (e.g. furlough) that would have impacted on receipt of sick notes over time. However, there is bias in who gets tested or seeks care for COVID-19 which will have changed as the pandemic evolved,(14) especially after cessation of free testing in April 2022. It is unclear how this would correlate with the likelihood of requesting or requiring a sick note. To mitigate this, we relied on three different methods for identifying COVID-19 cases (positive PCR or LFT test, primary care diagnosis, hospitalisation), but many mild or asymptomatic cases, and cases among people choosing not to get tested or not to record their LFT result will be missed.

We also could not identify whether people were participating in the workforce and included everyone of working age. From 2019 to 2022, the employment rate was 62% in people 18-24 years, 85% in people 25-49 years, and 72% in people 50-64 years.(15) It is therefore likely that the true absolute sick note rate will be substantially attenuated, especially among the younger age groups. However, this will only impact on the relative effects if the employment rate differs substantially between the COVID-19 cohorts and general population. Last, people with very severe symptoms who need to leave the workforce completely may also not be captured in our data, but these counts are likely to be small.

Findings in context

The introduction of vaccination programmes and differences in circulating variants lead to changes in severity and transmissibility of SARS-CoV-2. While infection rate went up during the study period, the COVID-19-related mortality rate has decreased in each subsequent wave, in large part due to greater vaccination coverage.(7) This reduced severity helps explain the lower sick note rate in later years. Additionally, the ending of lockdowns and other preventive measures, cessation of free testing, and differential uptake of vaccines by demographic and clinical subgroups will have impacted on the characteristics of people

For peer review only - http://bmjopen.bmj.com/site/about/guidelines.xhtml

testing positive for SARS-CoV-2.(16) Consistent with previously reported data,(17) we observed that the number of people with evidence of a positive SARS-CoV-2 test was much greater in 2021 and 2022 compared with 2020. Thus, while the sick note rate was nearly twice as high in 2020, the actual number of people issued a sick note was 4.5 times higher in 2022. Although people hospitalised with pneumonia were older and had more comorbidities, after adjustment for demographics and clinical characteristics people hospitalised with COVID-19 were still less likely to be issued a sick note than people with pneumonia, especially in 2022 suggesting the long-term effects are not worse than comparable serious respiratory infections requiring hospitalisation.

In contrast, the sick note rate was lower in the general population in 2020, compared with other years. This is consistent with a previous study of NHS workers which identified a decrease in sick notes for non-COVID-related episodes in 2020.(18) There are likely to be several explanations such as changes to working patterns, including furlough and remote working, which would have reduced the need for sick notes. There were also fewer circulating non-COVID-19 respiratory infections in 2020 due to public health measures.(19) However, a previous study identified that while the sick note rate decreased overall in 2020, sick notes associated with certain diagnoses, specifically asthma, respiratory conditions, and mental health increased during the COVID-19 pandemic.(18) Similarly, a study of NHS workers found that sickness absences due to psychiatric illness was higher in 2022 compared with pre-pandemic.(20)

Direct comparisons with studies of sickness absences in other jurisdictions are difficult due to differences in study populations, follow-up periods, and how sick leave is defined. In a large Swedish study (n=661,780), 6% experienced long-term sick leave associated with COVID-19, more common in women, older people, and people with lower incomes, more comorbidities, or hospitalised with COVID-19.(21) Other studies focussed either on people with COVID-19 only, or certain populations. In Wales, 15% of domiciliary care workers (n=15,931) were issued a sick note between March 2020 and November 2021, and they were more common in women and older people.(22) In Germany, a study of 30,950 people with COVID-19 found that 6% experienced long-term sick leave.(23)

Policy implications and future research

Now several years into the pandemic, vaccines have led to reduced severity of COVID-19, but the number of daily cases remains high,(16) potentially putting many people at risk of long-term symptoms. The incidence of repeat infections is also increasing;(24) while a repeat infection appears to lead to milder acute symptoms,(25,26) other studies have found that the long-term symptom burden increases with the number of reinfections.(27,28) In our study, we did not try and link the reason for the sick note to COVID-19. However, most studies of long-term effects have focussed on surveys and self-report, which can be unrepresentative(29–31) and long COVID is not well coded in electronic health records.(10) Thus, our findings provide a different perspective on the potential long-term consequences of COVID-19 that does not rely on accurate coding of COVID-19 related symptoms.

In the UK, the sickness absence rate in 2022 is the highest it has been since 2004.(32) The populations with the highest absolute rates of sick notes after COVID-19 (women, people living in areas of greater deprivation) are those over-represented in low-income and/or

For peer review only - http://bmjopen.bmj.com/site/about/guidelines.xhtml

public-facing jobs, which have higher rates of sickness absences.(33) Mental health problems are both a common cause of long-term sickness absence(1,34) and a predictor of long COVID(31), pointing to unmet needs in these populations. However, although people living in areas of least deprivation with COVID-19 had the lowest absolute sick note rate, they had the greatest relative increase compared with the general population in earlier years, reflecting their lower baseline rate; this pattern disappeared in 2022.

Pressures on primary care are also increasing, and over the past decade the number of GPs has gone down and each GP is thus responsible for a greater number of patients.(35) It's unclear to what extent the increasing number of people experiencing long-term post-acute symptoms contributes to the burden on primary care. Thus, we need to understand not only how people with long COVID symptoms interact with primary care, but how their symptoms are recorded in electronic health records to inform future studies. In addition, further investigation into the duration of sick notes, as well as the diagnoses associated with the issued sick note, whether these changed over time from diagnosis, and how these compare to sick notes issued to people without COVID-19 will help shed light on the most common long-term symptoms experienced.

Conclusion

Despite undercapture of people with COVID-19 and overestimation of the number of people in the workforce, we have identified a considerable increased risk of sick notes among people with COVID-19 compared with the general population. This extends into an era of high vaccination rates and when the severity of illness from COVID-19 is decreasing. The majority of sick notes were issued within the first 30 days post-diagnosis, suggesting that most COVID-19 related sick leave is associated with the acute phase of the disease. However, a persistent increased risk up to 10 months after the illness demonstrates the ongoing health and economic impact of COVID-19.

Ethics approval

This study was approved by the Health Research Authority (Research Ethics Committee reference 20/LO/0651) and by the London School of Hygiene and Tropical Medicine Ethics Board (reference 21863).

Acknowledgements

We are very grateful for all the support received from the TPP Technical Operations team throughout this work, and for generous assistance from the information governance and database teams at NHS England and the NHS England Transformation Directorate

For peer review only

References

1. NHS Digital. Fit Notes Issued by GP Practices, England, September 2022 [Internet]. [cited 2023 Mar 17]. Available from: https://digital.nhs.uk/data-and-information/publications/statistical/fit-notes-issued-by-gp-practices/september-2022

2. Dorrington S, Roberts E, Mykletun A, Hatch S, Madan I, Hotopf M. Systematic review of fit note use for workers in the UK. Occup Environ Med. 2018 Jul 1;75(7):530–9.

3. Henderson M, Clark C, Stansfeld S, Hotopf M. A lifecourse approach to long-term sickness absence--a cohort study. PLoS One. 2012;7(5):e36645.

4. UK Department for Work and Pensions. An evaluation of the Statement of Fitness for Work (fit note): Survey of employees (RR 840) [Internet]. [cited 2023 Mar 6]. Report No.: 840. Available from: https://www.gov.uk/government/publications/an-evaluation-of-the-statement-of-fitness-for-work-fit-note-survey-of-employees-rr-840

5. Office for National Statistics. Cumulative COVID-19 infections time series, England [Internet]. 2023 [cited 2023 Jul 6]. Available from: https://www.ons.gov.uk/peoplepopulationandcommunity/healthandsocialcare/conditionsanddiseases/datasets/cumulativecovid19infectionstimeseriesengland

6. Office for National Statistics. Prevalence of ongoing symptoms following coronavirus (COVID-19) infection in the UK [Internet]. 2023 [cited 2023 Mar 31]. Available from: https://www.ons.gov.uk/peoplepopulationandcommunity/healthandsocialcare/conditionsanddiseases/bulletins/prevalenceofongoingsymptomsfollowingcoronaviruscovid19infectionintheuk/latest

7. Nab L, Parker EPK, Andrews CD, Hulme WJ, Fisher L, Morley J, et al. Changes in COVID-19-related mortality across key demographic and clinical subgroups in England from 2020 to 2022: a retrospective cohort study using the OpenSAFELY platform. The Lancet Public Health. 2023 May 1;8(5):e364–77.

8. Michelen M, Manoharan L, Elkheir N, Cheng V, Dagens A, Hastie C, et al. Characterising long COVID: a living systematic review. BMJ Global Health. 2021 Sep 1;6(9):e005427.

9. Davis HE, McCorkell L, Vogel JM, Topol EJ. Long COVID: major findings, mechanisms and recommendations. Nat Rev Microbiol. 2023 Mar;21(3):133–46.

10. Walker AJ, MacKenna B, Inglesby P, Tomlinson L, Rentsch CT, Curtis HJ, et al. Clinical coding of long COVID in English primary care: a federated analysis of 58 million patient records in situ using OpenSAFELY. Br J Gen Pract. 2021 Nov 1;71(712):e806–14.

11. UK Department for Work and Pensions. GOV.UK. 2023 [cited 2024 May 9]. The fit note: guidance for patients and employees. Available from: https://www.gov.uk/government/publications/the-fit-note-a-guide-for-patients-and-employees/the-fit-note-guidance-for-patients-and-employees

12. Hernán MA. The Hazards of Hazard Ratios. Epidemiology. 2010 Jan;21(1):13–5.

13. Andrews C, Schultze A, Curtis H, Hulme W, Tazare J, Evans S, et al. OpenSAFELY: Representativeness of electronic health record platform OpenSAFELY-TPP data compared to the population of England. Wellcome Open Res. 2022;7:191.

For peer review only - http://bmjopen.bmj.com/site/about/guidelines.xhtml

14. Griffith GJ, Morris TT, Tudball MJ, Herbert A, Mancano G, Pike L, et al. Collider bias undermines our understanding of COVID-19 disease risk and severity. Nat Commun. 2020 Nov 12;11(1):5749.

15. Office for National Statistics. A05 SA: Employment, unemployment and economic inactivity by age group (seasonally adjusted) [Internet]. [cited 2023 May 17]. Available from: https://www.ons.gov.uk/employmentandlabourmarket/peopleinwork/employmentandemployeetypes/datasets/employmentunemploymentandeconomicinactivitybyagegroupseasonallyadjusteda05sa

16. UK Health Security Agency. England Summary | Coronavirus (COVID-19) in the UK [Internet]. 2023 [cited 2023 Mar 6]. Available from: https://coronavirus.data.gov.uk

17. Office for National Statistics. Coronavirus (COVID-19) latest insights [Internet]. 2023 [cited 2023 Mar 31]. Available from: https://www.ons.gov.uk/peoplepopulationandcommunity/healthandsocialcare/conditionsanddiseases/articles/coronaviruscovid19latestinsights/infections

18. Edge R, van der Plaat DA, Parsons V, Coggon D, van Tongeren M, Muiry R, et al. Changing patterns of sickness absence among healthcare workers in England during the COVID-19 pandemic. J Public Health (Oxf). 2022 Mar 7;44(1):e42–50.

19. Kadambari S, Goldacre R, Morris E, Goldacre MJ, Pollard AJ. Indirect effects of the covid-19 pandemic on childhood infection in England: population based observational study. BMJ. 2022 Jan 12;376:e067519.

20. Palmer W, Rolewicz L. Nuffield Trust. 2023 [cited 2023 Jun 30]. All is not well: Sickness absence in the NHS in England. Available from: https://www.nuffieldtrust.org.uk/resource/all-is-not-well-sickness-absence-in-the-nhs-in-england

21. Spetz M, Natt och Dag Y, Li H, Nwaru C, Santosa A, Nyberg F, et al. The sociodemographic patterning of sick leave and determinants of longer sick leave after mild and severe COVID-19: a nationwide register-based study in Sweden. European Journal of Public Health. 2024 Feb 1;34(1):121–8.

22. Cannings-John R, Schoenbuchner S, Jones H, Lugg-Widger FV, Akbari A, Brookes-Howell L, et al. Impact of the COVID-19 pandemic on domiciliary care workers in Wales, UK: a data linkage cohort study using the SAIL Databank. BMJ Open. 2023 Jun 1;13(6):e070637.

23. Jacob L, Haro JM, Koyanagi A. Physical multimorbidity and subjective cognitive complaints among adults in the United Kingdom: a cross-sectional community-based study. Sci Rep. 2019 Aug 27;9(1):12417.

24. Coronavirus (COVID-19) Infection Survey, characteristics of people testing positive for COVID-19, UK - Office for National Statistics [Internet]. [cited 2023 May 17]. Available from: https://www.ons.gov.uk/peoplepopulationandcommunity/healthandsocialcare/conditionsanddiseases/bulletins/coronaviruscovid19infectionsurveycharacteristicsofpeopletestingpositiveforcovid19uk/21september2022

For peer review only

25. Abu-Raddad LJ, Chemaitelly H, Bertollini R, National Study Group for COVID-19 Epidemiology. Severity of SARS-CoV-2 Reinfections as Compared with Primary Infections. N Engl J Med. 2021 Dec 23;385(26):2487–9.

26. Mensah AA, Lacy J, Stowe J, Seghezzo G, Sachdeva R, Simmons R, et al. Disease severity during SARS-COV-2 reinfection: a nationwide study. J Infect. 2022 Apr;84(4):542–50.

27. Willyard C. Are repeat COVID infections dangerous? What the science says. Nature. 2023 Apr 26;616(7958):650–2.

28. Bowe B, Xie Y, Al-Aly Z. Acute and postacute sequelae associated with SARS-CoV-2 reinfection. Nat Med. 2022 Nov;28(11):2398–405.

29. Sudre CH, Murray B, Varsavsky T, Graham MS, Penfold RS, Bowyer RC, et al. Attributes and predictors of long COVID. Nat Med. 2021 Apr;27(4):626–31.

30. Whitaker M, Elliott J, Chadeau-Hyam M, Riley S, Darzi A, Cooke G, et al. Persistent COVID-19 symptoms in a community study of 606,434 people in England. Nat Commun. 2022 Apr 12;13(1):1957.

31. Thompson EJ, Williams DM, Walker AJ, Mitchell RE, Niedzwiedz CL, Yang TC, et al. Long COVID burden and risk factors in 10 UK longitudinal studies and electronic health records. Nat Commun. 2022 Jun 28;13(1):3528.

32. Sickness absence in the UK labour market - Office for National Statistics [Internet]. [cited 2023 Oct 13]. Available from: https://www.ons.gov.uk/employmentandlabourmarket/peopleinwork/labourproductivity/articles/sicknessabsenceinthelabourmarket/2022

33. Office for National Statistics. Half a million more people are out of the labour force because of long-term sickness [Internet]. 2022 [cited 2024 May 9]. Available from: https://www.ons.gov.uk/employmentandlabourmarket/peoplenotinwork/economicinactivity/articles/halfamillionmorepeopleareoutofthelabourforcebecauseoflongtermsickness/2022-11-10

34. Dorrington S, Carr E, Polling C, Stevelink S, Ashworth M, Roberts E, et al. Health condition at first fit note and number of fit notes: a longitudinal study of primary care records in south London. BMJ Open. 2021 Mar 1;11(3):e043889.

35. British Medical Association. Pressures in general practice data analysis [Internet]. 2024 [cited 2024 May 9]. Available from: https://www.bma.org.uk/advice-and-support/nhs-delivery-and-workforce/pressures/pressures-in-general-practice-data-analysis

For peer review only

Administrative

Author contributions

Conceptualization: AJW, LAT, JM, FG, SD; Data curation: ALS, RYP, AJW, SCJB, ID, CB, BG; Formal analysis: ALS, RYP, AJW, JT; Funding acquisition: BG; Investigation: ALS, AJW, RYP; Methodology: ALS, AJW, LAT, RYP, JM, FG, JT, KB; Project administration: AJW, LAT, AM, BG; Resources: AJW, ID, SCJB, BG; Software: ALS, RYP, AJW, JT, SCJB, ID, CB; Supervision: AJW, LAT, BMK, AM, BG; Validation: ALS, RYP, AJW; Visualisation: ALS, RYP, AJW; Writing - original draft: ALS, RYP, AJW; Writing - review & editing: All authors. All authors gave final approval of the version to be published and agree to be accountable for all aspects of the work. All authors confirm that they had full access to all the data in the study and accept responsibility to submit for publication.

Conflicts of interest

Over the past five years BG has received research funding from the Laura and John Arnold Foundation, the NHS National Institute for Health Research (NIHR), the NIHR School of Primary Care Research, the NIHR Oxford Biomedical Research Centre, the Mohn-Westlake Foundation, NIHR Applied Research Collaboration Oxford and Thames Valley, the Wellcome Trust, the Good Thinking Foundation, Health Data Research UK (HDRUK), the Health Foundation, and the World Health Organisation; he also receives personal income from speaking and writing for lay audiences on the misuse of science. CB is an employee of TPP. BMK is also employed by NHS England working on medicines policy and clinical lead for primary care medicines data.

Funding

The OpenSAFELY Platform is supported by grants from the Wellcome Trust (222097/Z/20/Z) and MRC (MR/V015737/1, MC_PC_20059, MR/W016729/1). In addition, development of OpenSAFELY has been funded by the Longitudinal Health and Wellbeing strand of the National Core Studies programme (MC_PC_20030, MC_PC_20059), the NIHR funded CONVALESCENCE programme (COV-LT-0009), NIHR (NIHR135559, COV-LT2-0073), and the Data and Connectivity National Core Study funded by UK Research and Innovation (MC_PC_20058), and Health Data Research UK (HDRUK2021.000, 2021.0157). BG's work on better use of data in healthcare more broadly is currently funded in part by: the Wellcome Trust, NIHR Oxford Biomedical Research Centre, NIHR Applied Research Collaboration Oxford and Thames Valley, the Mohn-Westlake Foundation; all University of Oxford, Bennett Institute for Applied Data Science staff are supported by BG's grants on this work. RYP is supported by the EPSRC Centre for Doctoral Training in Health Data Science (EP/S02428X/1). FG is supported by the NIHR Applied Research Collaborative for North West London. SD is supported by: a) the BHF Data Science Centre led by HDR UK (grant SP/19/3/34678), b) NIHR University College London Hospitals Biomedical Research Centre, c) BHF Accelerator Award (AA/18/6/24223), d) the NIHR-UKRI CONVALESCENCE study. KB is funded by a Wellcome Senior Research Fellowship (220283/Z/20/Z).

The views expressed are those of the authors and not necessarily those of the NIHR, NHS England, Public Health England or the Department of Health and Social Care. Funders had

For peer review only - http://bmjopen.bmj.com/site/about/guidelines.xhtml

no role in the study design, collection, analysis, and interpretation of data; in the writing of the report; and in the decision to submit the article for publication.

For peer review only

Table 1. Demographic and select clinical characteristics of general population COVID-19 and comparator cohorts, 1 Feb - 30 Nov of each year. General population comparator cohorts were frequency matched on age, sex, and administrative region. Additional health conditions are presented in Supplementary Table 1. All counts rounded to nearest 7.

	**2019**	**2020**		**2021**		**2022**	
	**General population***	**COVID-19 cohort**	**General population^**	**COVID-19 cohort**	**General population^**	**COVID-19 cohort**	**General population^**
	**n (%)**	**n (%)**	**n (%)**	**n (%)**	**n (%)**	**n (%)**	**n (%)**
**Total**	3,140,326 (100.0)	365,421 (100.0)	3,439,534 (100.0)	1,206,555 (100.0)	4,571,469 (100.0)	1,321,313 (100.0)	4,818,870 (100.0)
**Age**							
18-24 y	496,825 (15.8)	61,355 (16.8)	545,286 (15.9)	196,616 (16.3)	699,993 (15.3)	111,342 (8.4)	375,704 (7.8)
25-34 y	663,495 (21.1)	77,686 (21.3)	726,712 (21.1)	264,159 (21.9)	989,506 (21.6)	258,314 (19.5)	915,152 (19.0)
35-44 y	644,945 (20.5)	74,767 (20.5)	706,692 (20.5)	294,238 (24.4)	1,116,283 (24.4)	312,732 (23.7)	1,142,764 (23.7)
45-54 y	719,663 (22.9)	83,832 (22.9)	787,465 (22.9)	271,418 (22.5)	1,042,601 (22.8)	328,510 (24.9)	1,197,693 (24.9)
55-64 y	615,405 (19.6)	67,781 (18.5)	673,372 (19.6)	180,117 (14.9)	723,093 (15.8)	310,422 (23.5)	1,187,557 (24.6)
**Sex**							
Female	1,732,654 (55.2)	201,824 (55.2)	1,898,351 (55.2)	645,666 (53.5)	2,447,200 (53.5)	823,438 (62.3)	3,001,922 (62.3)
Male	1,407,672 (44.8)	163,597 (44.8)	1,541,183 (44.8)	560,889 (46.5)	2,124,269 (46.5)	497,875 (37.7)	1,816,948 (37.7)
**Ethnicity**							
White	1,914,458 (61.0)	213,185 (58.3)	2,109,478 (61.3)	788,249 (65.3)	2,866,892 (62.7)	967,225 (73.2)	3,287,305 (68.2)
Asian or Asian British	225,316 (7.2)	45,101 (12.3)	254,884 (7.4)	68,915 (5.7)	315,854 (6.9)	52,101 (3.9)	300,167 (6.2)
Black	67,319 (2.1)	8099 (2.2)	76,622 (2.2)	21,399 (1.8)	103,117 (2.3)	18,340 (1.4)	105,322 (2.2)
Mixed	34,748 (1.1)	4298 (1.2)	39,872 (1.2)	13,937 (1.2)	56,644 (1.2)	14,077 (1.1)	58,527 (1.2)
Other	60,935 (1.9)	5502 (1.5)	70,427 (2.0)	14,910 (1.2)	102,585 (2.2)	19,425 (1.5)	105,007 (2.2)
Unknown	837,550 (26.7)	89,236 (24.4)	888,265 (25.8)	299,145 (24.8)	1,126,384 (24.6)	250,152 (18.9)	962,542 (20.0)
**IMD**							
1 (most deprived)	795,739 (25.3)	98,399 (26.9)	877,583 (25.5)	257,992 (21.4)	1,027,467 (22.5)	212,044 (16.0)	929,649 (19.3)
2	630,189 (20.1)	76,874 (21.0)	691,131 (20.1)	243,334 (20.2)	926,135 (20.3)	251,692 (19.0)	968,303 (20.1)
3	605,759 (19.3)	68,887 (18.9)	661,150 (19.2)	248,696 (20.6)	935,067 (20.5)	292,131 (22.1)	1,047,914 (21.7)
4	57,9614 (18.5)	64,064 (17.5)	633,059 (18.4)	235,473 (19.5)	877,170 (19.2)	286,538 (21.7)	975,576 (20.2)
5 (least deprived)	52,9025 (16.8)	57,190 (15.7)	576,618 (16.8)	221,067 (18.3)	805,623 (17.6)	278,915 (21.1)	897,421 (18.6)
**Region**							
East	432,551 (13.8)	50,246 (13.8)	473,928 (13.8)	240,170 (19.9)	910,567 (19.9)	327,096 (24.8)	1,184,981 (24.6)
East Midlands	645,218 (20.5)	75,040 (20.5)	706,370 (20.5)	227,577 (18.9)	863,884 (18.9)	222,397 (16.8)	814,919 (16.9)

For peer review only

For peer review only - http://bmjopen.bmj.com/site/about/guidelines.xhtml

London	145,264 (4.6)	17,073 (4.7)	158,844 (4.6)	56,693 (4.7)	210,623 (4.6)	71,113 (5.4)	255,997 (5.3)
North East	257,985 (8.2)	29,988 (8.2)	282,198 (8.2)	71,169 (5.9)	270,284 (5.9)	59,059 (4.5)	216,216 (4.5)
North West	426,615 (13.6)	49,623 (13.6)	467,383 (13.6)	129,934 (10.8)	494,144 (10.8)	108,857 (8.2)	399,168 (8.3)
South East	101,976 (3.2)	11,942 (3.3)	111,986 (3.3)	70,763 (5.9)	266,098 (5.8)	100,114 (7.6)	363,867 (7.6)
South West	220,759 (7.0)	25,641 (7.0)	241,500 (7.0)	160,678 (13.3)	608,790 (13.3)	227,731 (17.2)	833,742 (17.3)
West Midlands	175,581 (5.6)	20,559 (5.6)	192,836 (5.6)	51,737 (4.3)	194,593 (4.3)	40,880 (3.1)	147,791 (3.1)
Yorkshire & The Humber	734,370 (23.4)	85,309 (23.3)	804,496 (23.4)	197,827 (16.4)	752,486 (16.5)	164,073 (12.4)	602175 (12.5)
**Common health conditions**							
Obesity	681,422 (21.7)	96,712 (26.5)	748,398 (21.8)	276,759 (22.9)	957,194 (20.9)	350,777 (26.5)	1,137,710 (23.6)
Hypertension	325,969 (10.4)	43,610 (11.9)	353,416 (10.3)	112,147 (9.3)	411,803 (9.0)	164,367 (12.4)	564,823 (11.7)
Diabetes	184,450 (5.9)	30,590 (8.4)	210,532 (6.1)	70,861 (5.9)	262,906 (5.8)	100,541 (7.6)	358,449 (7.4)
Asthma	538,657 (17.2)	68,208 (18.7)	595,763 (17.3)	236,439 (19.6)	807,618 (17.7)	279,944 (21.2)	852,586 (17.7)

*age, sex and STP frequency matched with 2020 COVID-19 cohort; ^age, sex and STP frequency matched with contemporary COVID-19 cohort

For peer review only

Table 2. Rate of first sick note per 100 person-months for each cohort by demographics

	2019	2020		2021		2022	
	General population^	COVID-19 cohort	General population^	COVID-19 cohort	General population^	COVID-19 cohort	General population^
	Rate per 100 person-months (95%CI)	Rate per 100 person-months (95%CI)	Rate per 100 person-months (95%CI)	Rate per 100 person-months (95%CI)	Rate per 100 person-months (95%CI)	Rate per 100 person-months (95%CI)	Rate per 100 person-months (95%CI)
**Total**	1.21 (1.21 - 1.21)	4.88 (4.83 - 4.93)	0.93 (0.93 - 0.93)	2.66 (2.64 - 2.67)	1.23 (1.23 - 1.23)	1.73 (1.72 - 1.73)	1.23 (1.23 - 1.23)
**Age**							
18-24 y	0.98 (0.97 - 0.99)	1.58 (1.50 - 1.65)	0.66 (0.65 - 0.66)	1.25 (1.23 - 1.28)	0.96 (0.95 - 0.98)	1.30 (1.28 - 1.33)	0.91 (0.90 - 0.92)
25-34 y	1.18 (1.16 - 1.19)	3.51 (3.42 - 3.61)	0.86 (0.85 - 0.87)	2.16 (2.13 - 2.19)	1.17 (1.16 - 1.18)	1.62 (1.60 - 1.64)	1.14 (1.13 - 1.15)
35-44 y	1.19 (1.18 - 1.20)	5.19 (5.07 - 5.31)	0.91 (0.90 - 0.92)	2.95 (2.92 - 2.99)	1.24 (1.23 - 1.25)	1.65 (1.63 - 1.66)	1.19 (1.18 - 1.20)
45-54 y	1.35 (1.34 - 1.37)	6.53 (6.40 - 6.65)	1.08 (1.07 - 1.09)	3.60 (3.55 - 3.64)	1.38 (1.37 - 1.39)	1.91 (1.90 - 1.93)	1.35 (1.34 - 1.36)
55-64 y	1.30 (1.28 - 1.31)	6.62 (6.48 - 6.75)	1.09 (1.07 - 1.10)	3.79 (3.74 - 3.85)	1.33 (1.32 - 1.35)	1.87 (1.85 - 1.89)	1.32 (1.32 - 1.33)
**Sex**							
Female	1.39 (1.38 - 1.40)	5.88 (5.80 - 5.95)	1.11 (1.10 - 1.11)	3.25 (3.22 - 3.27)	1.46 (1.46 - 1.47)	1.96 (1.95 - 1.97)	1.41 (1.41 - 1.42)
Male	0.99 (0.98 - 1.00)	3.61 (3.54 - 3.68)	0.72 (0.71 - 0.72)	2.03 (2.01 - 2.05)	0.96 (0.95 - 0.97)	1.35 (1.34 - 1.37)	0.94 (0.93 - 0.94)
**Ethnicity**							
White	1.30 (1.30 - 1.31)	5.17 (5.10 - 5.24)	1.00 (0.99 - 1.00)	2.79 (2.77 - 2.81)	1.31 (1.31 - 1.32)	1.73 (1.72 - 1.74)	1.29 (1.28 - 1.29)
Mixed	1.03 (1.01 - 1.04)	4.83 (4.69 - 4.97)	0.86 (0.84 - 0.87)	3.11 (3.05 - 3.18)	1.10 (1.08 - 1.12)	2.07 (2.02 - 2.12)	1.16 (1.14 - 1.18)
Asian / Asian British	1.24 (1.20 - 1.28)	5.51 (5.18 - 5.84)	0.91 (0.88 - 0.94)	3.47 (3.34 - 3.61)	1.29 (1.25 - 1.32)	2.22 (2.14 - 2.31)	1.42 (1.38 - 1.45)
Black	1.22 (1.16 - 1.27)	4.55 (4.11 - 4.98)	0.92 (0.88 - 0.96)	2.65 (2.51 - 2.80)	1.19 (1.15 - 1.23)	1.74 (1.66 - 1.83)	1.25 (1.21 - 1.29)
Other	0.62 (0.59 - 0.65)	3.91 (3.56 - 4.25)	0.47 (0.44 - 0.49)	2.31 (2.18 - 2.44)	0.66 (0.64 - 0.69)	1.30 (1.24 - 1.37)	0.75 (0.72 - 0.77)
Unknown	1.09 (1.08 - 1.10)	4.23 (4.13 - 4.33)	0.83 (0.82 - 0.84)	2.19 (2.16 - 2.22)	1.10 (1.09 - 1.11)	1.62 (1.60 - 1.64)	1.09 (1.08 - 1.10)
**IMD quintile**							
1 (most deprived)	1.58 (1.57 - 1.59)	5.27 (5.17 - 5.38)	1.20 (1.19 - 1.21)	3.13 (3.10 - 3.17)	1.66 (1.65 - 1.67)	2.45 (2.42 - 2.48)	1.71 (1.69 - 1.72)
2	1.33 (1.32 - 1.34)	5.07 (4.96 - 5.18)	1.02 (1.01 - 1.03)	2.84 (2.80 - 2.88)	1.35 (1.34 - 1.36)	2.01 (1.99 - 2.03)	1.38 (1.37 - 1.39)
3	1.12 (1.11 - 1.13)	4.84 (4.72 - 4.95)	0.87 (0.86 - 0.88)	2.57 (2.54 - 2.61)	1.13 (1.12 - 1.14)	1.65 (1.64 - 1.67)	1.15 (1.14 - 1.16)
4	1.00 (0.99 - 1.01)	4.65 (4.53 - 4.77)	0.77 (0.76 - 0.78)	2.38 (2.34 - 2.41)	1.02 (1.01 - 1.03)	1.50 (1.48 - 1.52)	1.03 (1.02 - 1.04)
5 (least deprived)	0.86 (0.85 - 0.88)	4.23 (4.11 - 4.35)	0.66 (0.65 - 0.67)	2.18 (2.14 - 2.22)	0.88 (0.87 - 0.89)	1.27 (1.26 - 1.29)	0.90 (0.89 - 0.91)
**Region**							
East Midlands	1.05 (1.03 - 1.06)	4.06 (3.94 - 4.17)	0.76 (0.75 - 0.77)	2.39 (2.36 - 2.43)	1.05 (1.04 - 1.05)	1.53 (1.51 - 1.54)	1.08 (1.07 - 1.09)

For peer review only - http://bmjopen.bmj.com/site/about/guidelines.xhtml

East	1.21 (1.19 - 1.22)	4.79 (4.67 - 4.91)	0.92 (0.91 - 0.93)	2.74 (2.71 - 2.78)	1.29 (1.28 - 1.30)	1.89 (1.87 - 1.91)	1.34 (1.33 - 1.35)
London	0.71 (0.69 - 0.73)	3.08 (2.90 - 3.26)	0.49 (0.48 - 0.51)	2.07 (2.01 - 2.14)	0.71 (0.69 - 0.73)	1.22 (1.19 - 1.25)	0.76 (0.74 - 0.77)
North East	1.34 (1.32 - 1.36)	5.32 (5.13 - 5.51)	1.06 (1.05 - 1.08)	2.88 (2.81 - 2.95)	1.43 (1.41 - 1.45)	2.09 (2.04 - 2.14)	1.52 (1.50 - 1.55)
North West	1.39 (1.38 - 1.41)	5.76 (5.61 - 5.92)	1.12 (1.11 - 1.14)	2.81 (2.76 - 2.86)	1.51 (1.50 - 1.53)	2.11 (2.08 - 2.14)	1.53 (1.51 - 1.54)
South East	0.96 (0.93 - 0.98)	3.73 (3.50 - 3.96)	0.72 (0.70 - 0.74)	2.24 (2.17 - 2.30)	1.00 (0.98 - 1.02)	1.44 (1.41 - 1.47)	1.03 (1.02 - 1.05)
South West	1.07 (1.05 - 1.09)	4.55 (4.37 - 4.74)	0.81 (0.79 - 0.82)	2.57 (2.52 - 2.62)	1.12 (1.11 - 1.13)	1.61 (1.59 - 1.63)	1.15 (1.14 - 1.16)
West Midlands	1.32 (1.29 - 1.34)	5.63 (5.38 - 5.88)	0.97 (0.95 - 0.99)	3.00 (2.92 - 3.07)	1.37 (1.34 - 1.39)	2.20 (2.15 - 2.26)	1.49 (1.46 - 1.52)
Yorkshire & The Humber	1.31 (1.30 - 1.33)	5.46 (5.34 - 5.58)	1.03 (1.02 - 1.04)	2.93 (2.89 - 2.97)	1.41 (1.39 - 1.42)	2.01 (1.98 - 2.03)	1.44 (1.43 - 1.45)

For peer review only

Table 3. Adjusted HRs for receipt of first sick note by follow-up period post-index date. Entire follow-up period was up to 10 months. Crude and age-sex adjusted HRs presented in Supplementary Table 6.

Comparison and follow-up period post-index date	Sick note rate per 100 person-months (95%CI)		Adjusted HR* (95%CI)
	COVID-19 cohort	General population	
**2020 COVID-19 cohort vs 2019 general population**			
0-30 days	9.35 (9.24 - 9.46)	1.99 (1.98 - 2.01)	4.68 (4.61 - 4.75)
0-90 days	6.20 (6.14 - 6.27)	1.48 (1.47 - 1.48)	3.63 (3.58 - 3.68)
0-150 days	5.39 (5.33 - 5.45)	1.31 (1.30 - 1.32)	3.36 (3.32 - 3.40)
Entire follow-up period	4.88 (4.83 - 4.93)	1.21 (1.20 - 1.22)	3.19 (3.15 - 3.23)
**2020 COVID-19 cohort vs 2020 general population**			
0-30 days	9.35 (9.24 - 9.46)	1.57 (1.55 - 1.58)	5.94 (5.85 - 6.03)
0-90 days	6.20 (6.14 - 6.27)	1.12 (1.11 - 1.13)	4.67 (4.61 - 4.73)
0-150 days	5.39 (5.33 - 5.45)	0.99 (0.99 - 1.00)	4.32 (4.27 - 4.38)
Entire follow-up period	4.88 (4.83 - 4.93)	0.93 (0.93 - 0.93)	4.07 (4.02 - 4.12)
**2021 COVID-19 cohort vs 2019 general population**			
0-30 days	6.51 (6.46 - 6.56)	1.99 (1.98 - 2.01)	3.34 (3.30 - 3.38)
0-90 days	3.50 (3.48 - 3.52)	1.48 (1.47 - 1.48)	2.38 (2.35 - 2.41)
0-150 days	2.91 (2.90 - 2.93)	1.31 (1.30 - 1.32)	2.15 (2.13 - 2.17)
Entire follow-up period	2.66 (2.64 - 2.67)	1.21 (1.20 - 1.22)	2.02 (2.00 - 2.04)
**2021 COVID-19 cohort vs 2021 general population**			
0-30 days	6.51 (6.46 - 6.56)	1.97 (1.96 - 1.99)	3.37 (3.33 - 3.40)
0-90 days	3.50 (3.48 - 3.52)	1.47 (1.46 - 1.48)	2.35 (2.33 - 2.37)
0-150 days	2.91 (2.90 - 2.93)	1.32 (1.31 - 1.33)	2.12 (2.10 - 2.13)
Entire follow-up period	2.66 (2.64 - 2.67)	1.23 (1.22 - 1.23)	1.99 (1.98 - 2.01)
**2022 COVID-19 cohort vs 2019 general population**			
0-30 days	4.87 (4.83 - 4.91)	1.99 (1.98 - 2.01)	2.27 (2.24 - 2.30)
0-90 days	2.57 (2.55 - 2.58)	1.48 (1.47 - 1.48)	1.73 (1.72 - 1.75)
0-150 days	2.05 (2.04 - 2.06)	1.31 (1.30 - 1.32)	1.63 (1.61 - 1.64)
Entire follow-up period	1.73 (1.72 - 1.73)	1.21 (1.20 - 1.22)	1.55 (1.54 - 1.57)
**2022 COVID-19 cohort vs 2022 general population**			

For peer review only - http://bmjopen.bmj.com/site/about/guidelines.xhtml

0-30 days	4.87 (4.83 - 4.91)	2.09 (2.07 - 2.10)	2.37 (2.34 - 2.39)
0-90 days	2.57 (2.55 - 2.58)	1.53 (1.52 - 1.54)	1.73 (1.73 - 1.76)
0-150 days	2.05 (2.04 - 2.06)	1.34 (1.39 - 1.35)	1.64 (1.62 - 1.65)
Entire follow-up period	1.73 (1.72 - 1.73)	1.23 (1.22 - 1.23)	1.57 (1.56 - 1.58)

^General population age, sex and STP frequency matched.

*Fully adjusted models include age, sex, IMD quintile, region, ethnicity, obesity, smoking status, hypertension, diabetes, chronic respiratory disease, asthma, chronic cardiac disease, lung cancer, haematological cancer, other cancer, chronic liver disease, other neurological disease, organ transplant, asplenia, HIV, permanent immunodeficiency, and rheumatoid arthritis/systemic lupus erythematosus/psoriasis.

For peer review only

Figure 1. Depiction of eleven cohorts and the historic (2019) and contemporary (2020, 2021, 2022) comparisons included in our analysis. There was no comparison between the COVID-19 hospitalised cohorts and pneumonia cohort due to the potential for misclassification between COVID-19 and pneumonia.

Figure 2. Fully adjusted hazard ratio of first sick note comparing COVID-19 cohorts to their contemporary and historical 2019 general population, stratified by demographic categories.

*All models are adjusted for age, sex, ethnicity, IMD quintile and region, but exclude the stratification variable. Models are additionally adjusted for obesity, smoking status, hypertension, diabetes, chronic respiratory disease, asthma, chronic cardiac disease, lung cancer, haematological cancer, other cancer, chronic liver disease, other neurological disease, organ transplant, asplenia, HIV, permanent immunodeficiency, and rheumatoid arthritis/systemic lupus erythematosus/psoriasis.

For peer review only

For peer review only

For peer review only - http://bmjopen.bmj.com/site/about/guidelines.xhtml

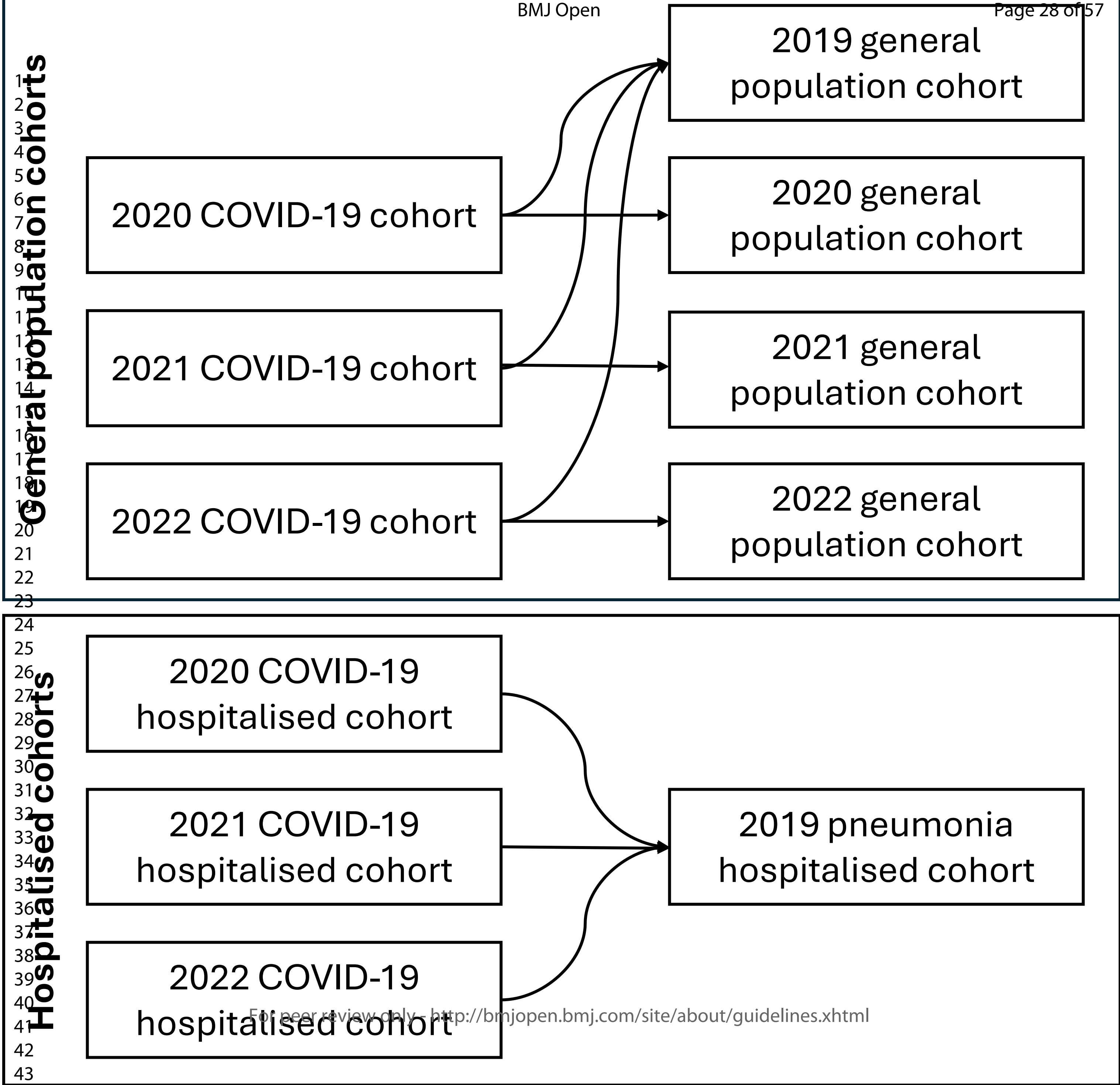
General population cohorts
2020 COVID-19 cohort
2021 COVID-19 cohort
2022 COVID-19 cohort
2019 general population cohort
2020 general population cohort
2021 general population cohort
2022 general population cohort
Hospitalised cohorts
2020 COVID-19 hospitalised cohort
2021 COVID-19 hospitalised cohort
2022 COVID-19 hospitalised cohort
2019 pneumonia hospitalised cohort

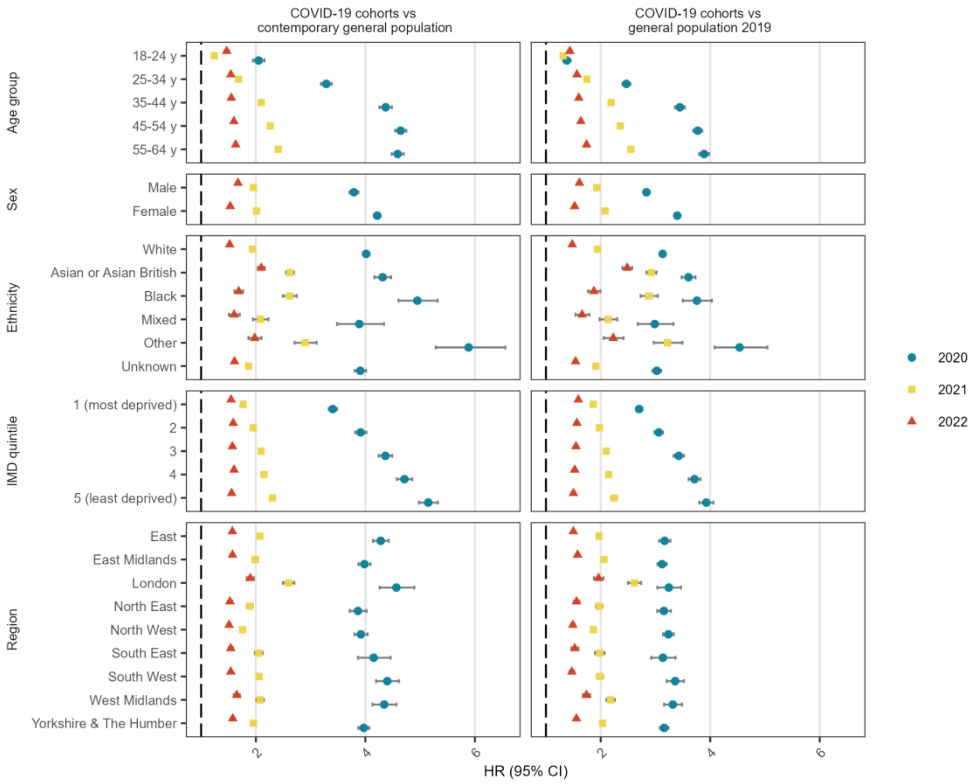

Fully adjusted hazard ratio of first sick note comparing COVID-19 cohorts to their contemporary or historical 2019 general population, stratified by demographic categories.

*All models are adjusted for age, sex, ethnicity, IMD quintile and region, but exclude the stratification variable. Models are additionally adjusted for obesity, smoking status, hypertension, diabetes, chronic respiratory disease, asthma, chronic cardiac disease, lung cancer, haematological cancer, other cancer, chronic liver disease, other neurological disease, organ transplant, asplenia, HIV, permanent immunodeficiency, and rheumatoid arthritis/systemic lupus erythematosus/psoriasis.

190x152mm (300 x 300 DPI)

Supplementary Files

Supplementary Figure 1. Source of first recorded positive SARS-CoV-2 test or COVID-19 diagnosis, by year. Some people have been identified by multiple sources and therefore the percentages within years may not add up to 100%. SGSS = Second Generation Surveillance System.

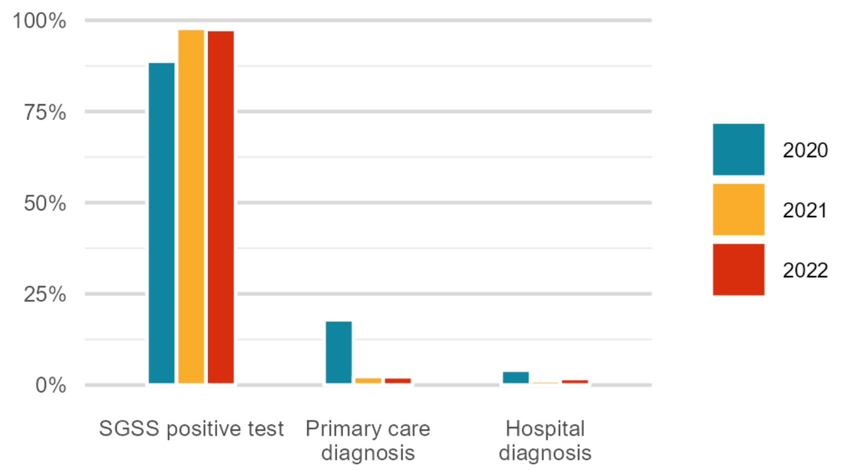

Supplementary Figure 2. Month of first recorded positive SARS-CoV-2 test or COVID-19 diagnosis by year

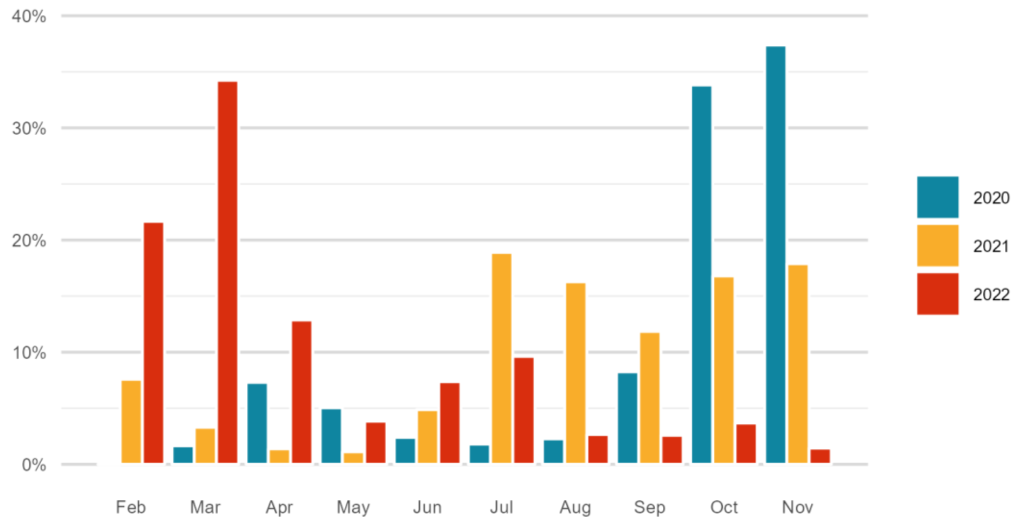

Supplementary Table 1. Select clinical characteristics of general population cohorts, 1 Feb - 30 Nov of each year. General population comparator cohorts were frequency-matched on age, sex, and administrative region. All counts rounded to nearest 7.

	2019	2020		2021		2022	
	**General population***	**COVID-19 cohort**	**General population^**	**COVID-19 cohort**	**General population^**	**COVID-19 cohort**	**General population^**
	**n (%)**	**n (%)**	**n (%)**	**n (%)**	**n (%)**	**n (%)**	**n (%)**
**Total**	3,140,326 (100.0)	365,421 (100.0)	3,439,534 (100.0)	1,206,555 (100.0)	4,571,469 (100.0)	1,321,313 (100.0)	4,818,870 (100.0)
**Health conditions**							
Other chronic respiratory disease	55,265 (1.8)	6650 (1.8)	59,773 (1.7)	15,932 (1.3)	67,361 (1.5)	27,083 (2.0)	90,755 (1.9)
Chronic cardiac disease	79,667 (2.5)	11,039 (3.0)	86,933 (2.5)	26,320 (2.2)	100,527 (2.2)	38,906 (2.9)	127,764 (2.7)
Lung cancer	1442 (0.0)	280 (0.1)	1610 (0.0)	483 (0.0)	1764 (0.0)	1050 (0.1)	2730 (0.1)
Haematological cancer	9212 (0.3)	1316 (0.4)	10,101 (0.3)	3444 (0.3)	12,964 (0.3)	7854 (0.6)	16,142 (0.3)
Other cancer	63,987 (2.0)	7798 (2.1)	69,923 (2.0)	22,351 (1.9)	84,742 (1.9)	39,599 (3.0)	125,146 (2.6)
Chronic liver disease	14,672 (0.5)	2093 (0.6)	16,380 (0.5)	4452 (0.4)	20,860 (0.5)	7637 (0.6)	26,110 (0.5)
Other neurological disease	21,945 (0.7)	2499 (0.7)	23,730 (0.7)	6678 (0.6)	29,827 (0.7)	14,364 (1.1)	35,924 (0.7)
Organ transplant	3339 (0.1)	679 (0.2)	3591 (0.1)	1554 (0.1)	4494 (0.1)	3885 (0.3)	5257 (0.1)
Asplenia	3416 (0.1)	427 (0.1)	3675 (0.1)	1127 (0.1)	4662 (0.1)	2191 (0.2)	5446 (0.1)
HIV	5404 (0.2)	714 (0.2)	6027 (0.2)	1799 (0.1)	7973 (0.2)	3304 (0.3)	8799 (0.2)
Other permanent immunodeficiency	2338 (0.1)	336 (0.1)	2730 (0.1)	1071 (0.1)	3689 (0.1)	1869 (0.1)	4158 (0.1)
Rheumatoid arthritis / SLE / psoriasis	137,536 (4.4)	16,982 (4.6)	150,787 (4.4)	56,203 (4.7)	193,893 (4.2)	71,022 (5.4)	229,537 (4.8)
**Smoking status**							
Current	637,581 (20.3)	47,915 (13.1)	684,236 (19.9)	194,327 (16.1)	895,923 (19.6)	192,381 (14.6)	910,245 (18.9)
Former	802,263 (25.5)	103,810 (28.4)	880,390 (25.6)	358,526 (29.7)	1,189,923 (26.0)	426,041 (32.2)	1,426,432 (29.6)
Never	1,536,297 (48.9)	194,747 (53.3)	1,672,286 (48.6)	581,210 (48.2)	2,185,834 (47.8)	661,794 (50.1)	2,280,509 (47.3)
Missing	164,185 (5.2)	18,942 (5.2)	202,622 (5.9)	72,485 (6.0)	299,789 (6.6)	41,097 (3.1)	201,684 (4.2)

*age, sex and STP frequency matched with 2020 COVID-19 cohort; ^age, sex and STP frequency matched with contemporary COVID-19 cohort; HIV = human immunodeficiency virus; SLE = systemic lupus erythematosus

For peer review only

Supplementary Table 2. Demographic and clinical characteristics of hospitalised cohorts, 1 Feb - 30 Nov of each year. All counts rounded to nearest 7.

	COVID-19 hospitalised cohorts			Pneumonia hospitalised cohort
	**2020**	**2021**	**2022**	**2019**
	**n (%)**	**n (%)**	**n (%)**	**n (%)**
**Total**	22,015 (100.0)	30,205 (100.0)	34,692 (100.0)	29,673 (100.0)
**Age**				
18-24 y	819 (3.7)	2107 (7.0)	2485 (7.2)	1106 (3.7)
25-34 y	2429 (11.0)	5635 (18.7)	6419 (18.5)	2618 (8.8)
35-44 y	3465 (15.7)	6307 (20.9)	6006 (17.3)	4158 (14.0)
45-54 y	6328 (28.7)	7329 (24.3)	7623 (22.0)	7798 (26.3)
55-64 y	8974 (40.8)	8827 (29.2)	12,166 (35.1)	13,986 (47.1)
**Sex**				
Female	10,059 (45.7)	15,442 (51.1)	20,531 (59.2)	14,294 (48.2)
Male	11,956 (54.3)	14,763 (48.9)	14,161 (40.8)	15,379 (51.8)
**Ethnicity**				
White	12,376 (56.2)	18,305 (60.6)	24,542 (70.7)	19,817 (66.8)
Asian or Asian British	3395 (15.4)	3374 (11.2)	2289 (6.6)	1589 (5.4)
Black	1008 (4.6)	1260 (4.2)	805 (2.3)	553 (1.9)
Mixed	343 (1.6)	532 (1.8)	406 (1.2)	217 (0.7)
Other	532 (2.4)	623 (2.1)	511 (1.5)	273 (0.9)
Unknown	4361 (19.8)	6118 (20.3)	6146 (17.7)	7217 (24.3)
**IMD**				
1 (most deprived)	7077 (32.1)	10,199 (33.8)	9478 (27.3)	8974 (30.2)
2	5040 (22.9)	6839 (22.6)	7525 (21.7)	6636 (22.4)
3	3962 (18.0)	5446 (18.0)	6951 (20.0)	5775 (19.5)
4	3269 (14.8)	4319 (14.3)	5957 (17.2)	4662 (15.7)
5 (least deprived)	2667 (12.1)	3395 (11.2)	4774 (13.8)	3626 (12.2)
**Region**				
East	3885 (17.6)	5341 (17.7)	7238 (20.9)	5866 (19.8)
East Midlands	4375 (19.9)	6090 (20.2)	6776 (19.5)	5698 (19.2)
London	1680 (7.6)	1715 (5.7)	1848 (5.3)	1624 (5.5)
North East	1568 (7.1)	2044 (6.8)	2219 (6.4)	1715 (5.8)
North West	2457 (11.2)	3339 (11.1)	3542 (10.2)	2926 (9.9)
South East	854 (3.9)	1421 (4.7)	2359 (6.8)	1701 (5.7)
South West	1631 (7.4)	3080 (10.2)	4326 (12.5)	3857 (13.0)
West Midlands	1547 (7.0)	2037 (6.7)	1771 (5.1)	1463 (4.9)
Yorkshire & The Humber	4025 (18.3)	5145 (17.0)	4613 (13.3)	4816 (16.2)
**Health conditions**				
Obesity	9870 (44.8)	12,600 (41.7)	11,452 (33.0)	9877 (33.3)
Hypertension	6594 (30.0)	6552 (21.7)	8435 (24.3)	8386 (28.3)
Diabetes	5726 (26.0)	5817 (19.3)	6846 (19.7)	6146 (20.7)
Asthma	4907 (22.3)	6755 (22.4)	8694 (25.1)	7581 (25.5)
Other chronic respiratory disease	1645 (7.5)	1722 (5.7)	3304 (9.5)	5460 (18.4)
Chronic cardiac disease	2324 (10.6)	2198 (7.3)	3780 (10.9)	4095 (13.8)
Lung cancer	154 (0.7)	126 (0.4)	301 (0.9)	735 (2.5)
Haematological cancer	371 (1.7)	469 (1.6)	1162 (3.3)	882 (3.0)
Other cancer	1323 (6.0)	1358 (4.5)	3199 (9.2)	3325 (11.2)
Chronic liver disease	623 (2.8)	595 (2.0)	1582 (4.6)	1477 (5.0)
Other neurological disease	651 (3.0)	595 (2.0)	1841 (5.3)	1260 (4.2)
Organ transplant	280 (1.3)	455 (1.5)	1253 (3.6)	490 (1.7)

For peer review only

Asplenia	77 (0.3)	91 (0.3)	203 (0.6)	189 (0.6)
HIV	14 (0.1)	14 (0.0)	49 (0.1)	21 (0.1)
Other permanent immunodeficiency	49 (0.2)	70 (0.2)	196 (0.6)	91 (0.3)
Rheumatoid arthritis / SLE / Psoriasis	1505 (6.8)	1967 (6.5)	3066 (8.8)	2569 (8.7)
**Smoking status**				
Current	3171 (14.4)	4788 (15.9)	9093 (26.2)	11,039 (37.2)
Former	7791 (35.4)	10,654 (35.3)	11,277 (32.5)	9233 (31.1)
Never	10,752 (48.8)	14,021 (46.4)	13,636 (39.3)	9058 (30.5)
Missing	301 (1.4)	742 (2.5)	679 (2.0)	336 (1.1)

HIV = human immunodeficiency virus; SLE = systemic lupus erythematosus

For peer review only

Supplementary Table 3. Number and percentage of people with a sick note in each cohort by demographic categories. The denominator is all people within each category. All counts rounded to the nearest 7.

	2021	2020		2021		2022	
	General population*	COVID-19 cohort	General population^	COVID-19 cohort	General population^	COVID-19 cohort	General population^
	n (%)	n (%)	n (%)	n (%)	n (%)	n (%)	n (%)
**Total**	181,167 (5.8)	34,377 (9.4)	153,447 (4.5)	102,949 (8.5)	259,812 (5.7)	152,859 (11.6)	273,889 (5.7)
**Age**							
18-24 y	23,807 (4.8)	1687 (2.7)	17,556 (3.2)	9765 (5.0)	31,458 (4.5)	10,262 (9.2)	16,499 (4.4)
25-34 y	37,212 (5.6)	5264 (6.8)	30,051 (4.1)	20,307 (7.7)	53,704 (5.4)	29,078 (11.3)	48,804 (5.3)
35-44 y	36,575 (5.7)	7413 (9.9)	30,919 (4.4)	25,732 (8.7)	63,924 (5.7)	35,672 (11.4)	62,881 (5.5)
45-54 y	46,347 (6.4)	10,843 (12.9)	40,586 (5.2)	27,489 (10.1)	66,381 (6.4)	41,195 (12.5)	74,326 (6.2)
55-64 y	37,226 (6.0)	9177 (13.5)	34,335 (5.1)	19,649 (10.9)	44,345 (6.1)	36,652 (11.8)	71,372 (6.0)
**Sex**							
Female	114,191 (6.6)	23,191 (11.5)	100,037 (5.3)	65,037 (10.1)	164,451 (6.7)	106,610 (12.9)	193,816 (6.5)
Male	66,976 (4.8)	11,186 (6.8)	53,410 (3.5)	37,912 (6.8)	95,361 (4.5)	46,249 (9.3)	80,080 (4.4)
**Ethnicity**							
White	118,272 (6.2)	20,622 (9.7)	100,681 (4.8)	67,522 (8.6)	173,194 (6.0)	112,063 (11.6)	193,599 (5.9)
Mixed	11,060 (4.9)	4662 (10.3)	10,409 (4.1)	8169 (11.9)	16,219 (5.1)	7000 (13.4)	16,310 (5.4)
Asian / Asian British	3955 (5.9)	1078 (13.3)	3325 (4.3)	2632 (12.3)	6160 (6.0)	2576 (14.0)	6895 (6.5)
Black	2016 (5.8)	420 (9.8)	1764 (4.4)	1274 (9.1)	3108 (5.5)	1624 (11.5)	3381 (5.8)
Other	1820 (3.0)	490 (8.9)	1596 (2.3)	1211 (8.1)	3241 (3.2)	1701 (8.8)	3689 (3.5)
Unknown	44,051 (5.3)	7112 (8.0)	35,672 (4.0)	22,127 (7.4)	57,890 (5.1)	27,888 (11.1)	50,022 (5.2)
**Region**							
East Midlands	21693 (5.0)	4767 (9.5)	17,423 (3.7)	17,577 (7.3)	44,625 (4.9)	34,139 (10.4)	59,633 (5.0)
East	37,121 (5.8)	6608 (8.8)	31,213 (4.4)	20426 (9.0)	51,457 (6.0)	27,720 (12.5)	50,442 (6.2)
London	4998 (3.4)	1148 (6.7)	3801 (2.4)	4053 (7.1)	7084 (3.4)	5957 (8.4)	9163 (3.6)
North East	16,373 (6.3)	3087 (10.3)	14,329 (5.1)	6979 (9.8)	17,724 (6.6)	7833 (13.3)	15,092 (7.0)
North West	28,168 (6.6)	5313 (10.7)	25,025 (5.4)	12,558 (9.7)	33,950 (6.9)	14,805 (13.6)	27,965 (7.0)

For peer review only

South East	4676 (4.6)	987 (8.3)	3913 (3.5)	4599 (6.5)	12,425 (4.7)	9996 (10.0)	17,430 (4.8)
South West	11,354 (5.1)	2254 (8.8)	9401 (3.9)	11,382 (7.1)	31,787 (5.2)	25,158 (11.0)	44,107 (5.3)
West Midlands	10,941 (6.2)	2002 (9.7)	8939 (4.6)	5481 (10.6)	12,229 (6.3)	5705 (14.0)	10,094 (6.8)
Yorkshire & The Humber	45,843 (6.2)	8204 (9.6)	39,403 (4.9)	19,887 (10.1)	48,524 (6.4)	21,539 (13.1)	39,956 (6.6)
**IMD quintile**							
1 (most deprived)	59,052 (7.4)	9989 (10.2)	50,148 (5.7)	28,728 (11.1)	77,714 (7.6)	33,229 (15.7)	72,597 (7.8)
2	39,767 (6.3)	7581 (9.9)	33,586 (4.9)	22,827 (9.4)	57,729 (6.2)	33,320 (13.2)	61,446 (6.3)
3	32,417 (5.4)	6503 (9.4)	27,699 (4.2)	20,083 (8.1)	49,301 (5.3)	32,578 (11.2)	55,762 (5.3)
4	27,853 (4.8)	5698 (8.9)	23,506 (3.7)	17,087 (7.3)	41,846 (4.8)	29,288 (10.2)	46,494 (4.8)
5 (least deprived)	22,078 (4.2)	4606 (8.1)	18,494 (3.2)	14,224 (6.4)	33,222 (4.1)	24,444 (8.8)	37,597 (4.2)

*age, sex and STP frequency matched with 2020 COVID-19 cohort; ^age, sex and STP frequency matched with contemporary COVID-19 cohort

For peer review only

Supplementary Table 4. Number of people issued a sick note and rate of first sick note per 100 person-months for hospitalised cohorts by demographic categories. The denominator for the percentages is all people within each category. All counts rounded to the nearest 7.

	**COVID-19 hospitalised cohorts**						**Pneumonia hospitalised cohort 2019**	
	**2020**		**2021**		**2022**		**2019**	
	**n (%)**	**Rate per 100 person-months (95%CI)**	**n (%)**	**Rate per 100 person-months (95%CI)**	**n (%)**	**Rate per 100 person-months (95%CI)**	**n (%)**	**Rate per 100 person-months (95%CI)**
**Total**	4606 (20.9)	6.78 (6.59 - 6.98)	7231 (23.9)	7.19 (7.03 - 7.36)	6664 (19.2)	4.13 (4.03 - 4.22)	7448 (25.1)	7.17 (7.01 - 7.34)
**Age**								
18-24 y	91 (11.1)	3.58 (2.84 - 4.31)	336 (15.9)	4.26 (3.80 - 4.71)	392 (15.8)	3.02 (2.72 - 3.31)	266 (24.1)	6.29 (5.53 - 7.04)
25-34 y	399 (16.4)	4.98 (4.49 - 5.47)	1050 (18.6)	5.27 (4.95 - 5.58)	1029 (16.0)	3.04 (2.86 - 3.23)	798 (30.5)	8.46 (7.88 - 9.05)
35-44 y	791 (18.1)	6.88 (6.41 - 7.36)	1589 (25.2)	7.75 (7.37 - 8.13)	1218 (20.3)	4.14 (3.90 - 4.37)	1253 (30.1)	8.66 (8.18 - 9.13)
45-54 y	1498 (23.7)	7.50 (7.12 - 7.88)	2107 (28.7)	9.07 (8.68 - 9.46)	1722 (22.6)	5.16 (4.92 - 5.41)	2100 (26.9)	7.76 (7.42 - 8.09)
55-64 y	1834 (20.4)	7.09 (6.76 - 7.41)	2149 (24.3)	7.42 (7.11 - 7.73)	2310 (19.0)	4.45 (4.27 - 4.63)	3031 (21.7)	6.23 (6.01 - 6.46)
**Sex**								
Female	2212 (22.0)	7.29 (6.99 - 7.60)	3430 (22.2)	6.64 (6.42 - 6.86)	3731 (18.2)	3.74 (3.62 - 3.86)	3556 (24.9)	7.00 (6.77 - 7.23)
Male	2394 (20.0)	6.37 (6.12 - 6.63)	3801 (25.7)	7.78 (7.53 - 8.03)	2940 (20.8)	4.75 (4.58 - 4.92)	3899 (25.4)	7.36 (7.12 - 7.59)
**Ethnicity**								
White	2611 (21.1)	6.99 (6.73 - 7.26)	4459 (24.4)	7.63 (7.41 - 7.86)	4704 (19.2)	4.12 (4.00 - 4.23)	4914 (24.8)	7.08 (6.88 - 7.28)
Mixed	651 (19.2)	6.28 (5.80 - 6.76)	721 (21.4)	5.36 (4.97 - 5.75)	427 (18.7)	4.07 (3.68 - 4.45)	350 (22.0)	5.76 (5.16 - 6.37)
Asian / Asian British	245 (24.3)	6.79 (5.94 - 7.64)	336 (26.7)	8.30 (7.41 - 9.18)	175 (21.7)	4.78 (4.07 - 5.49)	147 (26.6)	7.38 (6.19 - 8.57)
Black	77 (22.4)	6.79 (5.28 - 8.31)	112 (21.1)	6.28 (5.12 - 7.44)	77 (19.0)	3.89 (3.02 - 4.76)	56 (25.8)	7.17 (5.29 - 9.04)
Other	105 (19.7)	5.41 (4.37 - 6.44)	119 (19.1)	4.81 (3.94 - 5.67)	84 (16.4)	3.57 (2.81 - 4.34)	63 (23.1)	7.27 (5.48 - 9.06)
Unknown	910 (20.9)	6.74 (6.30 - 7.18)	1484 (24.3)	7.30 (6.92 - 7.67)	1204 (19.6)	4.18 (3.95 - 4.42)	1925 (26.7)	7.79 (7.44 - 8.13)
**Region**								
East Midlands	819 (21.1)	5.79 (5.39 - 6.18)	1246 (23.3)	7.35 (6.94 - 7.75)	1358 (18.8)	3.88 (3.67 - 4.09)	1512 (25.8)	7.30 (6.94 - 7.67)
East	854 (19.5)	6.39 (5.96 - 6.82)	1428 (23.4)	7.02 (6.65 - 7.38)	1372 (20.2)	4.48 (4.24 - 4.71)	1449 (25.4)	7.35 (6.97 - 7.73)
London	287 (17.1)	4.26 (3.77 - 4.75)	329 (19.2)	5.31 (4.74 - 5.89)	294 (15.9)	3.17 (2.81 - 3.54)	350 (21.6)	5.95 (5.33 - 6.57)
North East	322 (20.5)	7.79 (6.94 - 8.63)	497 (24.3)	6.91 (6.30 - 7.51)	392 (17.7)	3.81 (3.43 - 4.18)	420 (24.5)	7.11 (6.43 - 7.79)
North West	581 (23.6)	9.11 (8.37 - 9.85)	861 (25.8)	7.49 (6.99 - 7.99)	721 (20.4)	4.55 (4.22 - 4.88)	735 (25.1)	7.32 (6.79 - 7.85)
South East	168 (19.7)	5.20 (4.42 - 5.99)	329 (23.2)	6.87 (6.13 - 7.62)	427 (18.1)	3.65 (3.30 - 3.99)	434 (25.5)	7.16 (6.48 - 7.83)

For peer review only

South West	413 (25.3)	8.04 (7.27 - 8.82)	735 (23.9)	8.69 (8.06 - 9.32)	819 (18.9)	3.91 (3.64 - 4.18)	994 (25.8)	7.14 (6.69 - 7.58)
West Midlands	301 (20.7)	6.28 (5.57 - 6.99)	497 (24.4)	6.71 (6.12 - 7.30)	357 (20.2)	4.66 (4.18 - 5.15)	343 (23.4)	6.69 (5.98 - 7.40)
Yorkshire & The Humber	854 (21.2)	8.58 (8.00 - 9.15)	1316 (25.6)	7.45 (7.05 - 7.85)	938 (20.3)	4.64 (4.34 - 4.94)	1211 (25.1)	7.35 (6.93 - 7.76)
**IMD quintile**								
1 (most deprived)	1337 (18.9)	6.50 (6.16 - 6.85)	2352 (23.1)	6.54 (6.27 - 6.80)	1743 (20.6)	4.01 (3.82 - 4.20)	1967 (21.9)	6.13 (5.86 - 6.40)
2	1043 (20.7)	6.69 (6.28 - 7.10)	1645 (24.1)	7.35 (7.00 - 7.70)	1449 (19.3)	4.18 (3.96 - 4.39)	1673 (25.2)	7.16 (6.82 - 7.50)
3	896 (22.6)	7.22 (6.75 - 7.69)	1379 (25.3)	7.74 (7.33 - 8.14)	1330 (19.1)	4.05 (3.83 - 4.26)	1512 (26.2)	7.51 (7.13 - 7.88)
4	756 (23.1)	7.29 (6.77 - 7.81)	1057 (24.5)	7.71 (7.24 - 8.17)	1183 (19.9)	4.24 (3.99 - 4.48)	1288 (27.6)	8.08 (7.64 - 8.52)
5 (least deprived)	574 (21.5)	6.40 (5.88 - 6.93)	798 (23.5)	7.51 (6.99 - 8.03)	952 (19.9)	4.22 (3.95 - 4.49)	1015 (28.0)	8.25 (7.75 - 8.76)

For peer review only

Supplementary Table 5. Crude and adjusted HRs for receipt of first sick note by follow-up period post-index date among general population cohorts

Comparison and follow-up period post-index date	Sick note rate per 100 person-months (95%CI)		Crude HR (95%CI)	Age-sex adjusted HR (95%CI)
	COVID-19 cohort	General population		
**2020 COVID-19 cohort vs 2019 general population**				
0-30 days	9.35 (9.24 - 9.46)	1.99 (1.98 - 2.01)	4.59 (4.52 - 4.65)	4.77 (4.70 - 4.84)
0-90 days	6.20 (6.14 - 6.27)	1.48 (1.47 - 1.48)	3.59 (3.55 - 3.64)	3.69 (3.64 - 3.73)
0-150 days	5.39 (5.33 - 5.45)	1.31 (1.30 - 1.32)	3.35 (3.31 - 3.39)	3.41 (3.37 - 3.45)
Entire follow-up period	4.88 (4.83 - 4.93)	1.21 (1.20 - 1.22)	3.20 (3.16 - 3.24)	3.23 (3.20 - 3.27)
**2020 COVID-19 cohort vs 2020 general population**				
0-30 days	9.35 (9.24 - 9.46)	1.57 (1.55 - 1.58)	5.83 (5.74 - 5.92)	6.14 (6.05 - 6.24)
0-90 days	6.20 (6.14 - 6.27)	1.12 (1.11 - 1.13)	4.65 (4.59 - 4.71)	4.83 (4.77 - 4.89)
0-150 days	5.39 (5.33 - 5.45)	0.99 (0.99 - 1.00)	4.35 (4.30 - 4.40)	4.46 (4.40 - 4.51)
Entire follow-up period	4.88 (4.83 - 4.93)	0.93 (0.93 - 0.93)	4.13 (4.08 - 4.18)	4.19 (4.14 - 4.24)
**2021 COVID-19 cohort vs 2019 general population**				
0-30 days	6.51 (6.46 - 6.56)	1.99 (1.98 - 2.01)	3.24 (3.21 - 3.28)	3.33 (3.29 - 3.37)
0-90 days	3.50 (3.48 - 3.52)	1.48 (1.47 - 1.48)	2.27 (2.25 - 2.29)	2.39 (2.37 - 2.41)
0-150 days	2.91 (2.90 - 2.93)	1.31 (1.30 - 1.32)	2.06 (2.04 - 2.07)	2.17 (2.15 - 2.18)
Entire follow-up period	2.66 (2.64 - 2.67)	1.21 (1.20 - 1.22)	1.96 (1.95 - 1.98)	2.04 (2.03 - 2.06)
**2021 COVID-19 cohort vs 2021 general population**				
0-30 days	6.51 (6.46 - 6.56)	1.97 (1.96 - 1.99)	3.27 (3.24 - 3.31)	3.43 (3.40 - 3.47)
0-90 days	3.50 (3.48 - 3.52)	1.47 (1.46 - 1.48)	2.29 (2.28 - 2.31)	2.40 (2.38 - 2.42)
0-150 days	2.91 (2.90 - 2.93)	1.32 (1.31 - 1.33)	2.07 (2.06 - 2.09)	2.17 (2.16 - 2.19)
Entire follow-up period	2.66 (2.64 - 2.67)	1.23 (1.22 - 1.23)	1.97 (1.96 - 1.99)	2.06 (2.04 - 2.07)
**2022 COVID-19 cohort vs 2019 general population**				
0-30 days	4.87 (4.83 - 4.91)	1.99 (1.98 - 2.01)	2.46 (2.43 - 2.48)	2.14 (2.11 - 2.17)
0-90 days	2.57 (2.55 - 2.58)	1.48 (1.47 - 1.48)	1.79 (1.77 - 1.81)	1.63 (1.62 - 1.65)
0-150 days	2.05 (2.04 - 2.06)	1.31 (1.30 - 1.32)	1.65 (1.64 - 1.66)	1.53 (1.52 - 1.55)
Entire follow-up period	1.73 (1.72 - 1.73)	1.21 (1.20 - 1.22)	1.56 (1.55 - 1.57)	1.46 (1.45 - 1.47)
**2022 COVID-19 cohort vs 2022 general population**				

For peer review only

Comparison and follow-up period post-index date	Sick note rate per 100 person-months (95%CI)		Crude HR (95%CI)	Age-sex adjusted HR (95%CI)
	COVID-19 cohort	General population		
0-30 days	4.87 (4.83 - 4.91)	2.09 (2.07 - 2.10)	2.35 (2.32 - 2.37)	2.35 (2.33 - 2.38)
0-90 days	2.57 (2.55 - 2.58)	1.53 (1.52 - 1.54)	1.73 (1.71 - 1.74)	1.73 (1.72 - 1.74)
0-150 days	2.05 (2.04 - 2.06)	1.34 (1.39 - 1.35)	1.62 (1.60 - 1.63)	1.62 (1.61 - 1.63)
Entire follow-up period	1.73 (1.72 - 1.73)	1.23 (1.22 - 1.23)	1.55 (1.54 - 1.56)	1.55 (1.54 - 1.56)

^General population age, sex and STP frequency matched.

*Fully adjusted models include age, sex, IMD quintile, region, ethnicity, obesity, smoking status, hypertension, diabetes, chronic respiratory disease, asthma, chronic cardiac disease, lung cancer, haematological cancer, other cancer, chronic liver disease, other neurological disease, organ transplant, asplenia, HIV, permanent immunodeficiency, and rheumatoid arthritis/systemic lupus erythematosus/psoriasis.

For peer review only

Supplementary Table 6. Crude and adjusted HRs for receipt of first sick note for comparisons of hospitalised cohorts, overall and by follow-up time post-diagnosis

Time period	Sick note rate per 100 person-months (95%CI)		Crude HR (95%CI)	Age-sex adjusted HR (95%CI)	Fully adjusted HR* (95%CI)
	COVID-19 cohort	Comparator cohort			
**2020 COVID-19 cohort vs 2019 pneumonia cohort**					
0-30 days	18.84 (18.18 - 19.49)	22.06 (21.46 - 22.66)	0.85 (0.81 - 0.88)	0.83 (0.78 - 0.88)	0.72 (0.68 - 0.77)
0-90 days	11.44 (11.09 - 11.79)	12.47 (12.17 - 12.76)	0.86 (0.83 - 0.89)	0.83 (0.78 - 0.88)	0.74 (0.69 - 0.78)
0-150 days	8.66 (8.40 - 8.92)	9.28 (9.06 - 9.49)	0.88 (0.85 - 0.92)	0.84 (0.79 - 0.88)	0.75 (0.71 - 0.80)
Overall	6.78 (6.59 - 6.98)	7.17 (7.01 - 7.34)	0.90 (0.86 - 0.93)	0.85 (0.80 - 0.89)	0.77 (0.73 - 0.81)
**2021 COVID-19 cohort vs 2019 pneumonia cohort**					
0-30 days	21.39 (20.81 - 21.96)	22.06 (21.46 - 22.66)	0.97 (0.93 - 1.00)	0.98 (0.92 - 1.03)	0.83 (0.78 - 0.88)
0-90 days	11.65 (11.37 - 11.93)	12.47 (12.17 - 12.76)	0.93 (0.90 - 0.96)	0.92 (0.88 - 0.97)	0.81 (0.77 - 0.85)
0-150 days	9.03 (8.82 - 9.24)	9.28 (9.06 - 0.49)	0.93 (0.90 - 0.96)	0.91 (0.87 - 0.96)	0.80 (0.77 - 0.85)
Overall	7.19 (7.03 - 7.36)	7.17 (7.01 - 7.34)	0.94 (0.91 - 0.97)	0.92 (0.87 - 0.96)	0.81 (0.77 - 0.86)
**2022 COVID-19 cohort vs 2019 pneumonia cohort**					
0-30 days	12.92 (12.52 - 13.32)	22.06 (21.46 - 22.66)	0.59 (0.57 - 0.62)	0.58 (0.54 - 0.61)	0.56 (0.53 - 0.60)
0-90 days	7.05 (6.87 - 7.24)	12.47 (12.17 - 12.75)	0.61 (0.59 - 0.62)	0.59 (0.56 - 0.62)	0.58 (0.56 - 0.61)
0-150 days	5.22 (5.09 - 5.35)	9.28 (9.06 - 9.49)	0.63 (0.61 - 0.65)	0.61 (0.58 - 0.64)	0.60 (0.58 - 0.63)
Overall	4.13 (4.03 - 4.22)	7.17 (7.01 - 7.34)	0.65 (0.63 - 0.67)	0.63 (0.60 - 0.66)	0.62 (0.59 - 0.65)

*Fully adjusted models include age, sex, IMD quintile, region, ethnicity, obesity, smoking status, hypertension, diabetes, chronic respiratory disease, asthma, chronic cardiac disease, lung cancer, haematological cancer, other cancer, chronic liver disease, other neurological disease, organ transplant, asplenia, HIV, permanent immunodeficiency, and rheumatoid arthritis/systemic lupus erythematosus/psoriasis.

For peer review only

Supplementary Table 7. Crude and adjusted hazard ratio for first sick note for all comparisons, stratified by age group.

	COVID-19 cohort vs 2019 general population		COVID-19 cohort vs contemporary general population	
	**Crude HR (95%CI)**	**Fully adjusted HR (95%CI)**	**Crude HR (95%CI)**	**Fully adjusted HR (95%CI)**
**2020 COVID-19 cohort**				
18-24 y	1.31 (1.25 - 1.38)	1.38 (1.31 - 1.45)	2.01 (1.91 - 2.12)	2.05 (1.95 - 2.16)
25-34 y	2.44 (2.37 - 2.51)	2.47 (2.40 - 2.54)	3.31 (3.22 - 3.41)	3.29 (3.19 - 3.39)
35-44 y	3.47 (3.38 - 3.56)	3.45 (3.36 - 3.54)	4.46 (4.35 - 4.58)	4.37 (4.26 - 4.48)
45-54 y	3.83 (3.75 - 3.91)	3.77 (3.69 - 3.85)	4.77 (4.67 - 4.87)	4.64 (4.54 - 4.75)
55-64 y	4.05 (3.95 - 4.14)	3.89 (3.80 - 3.98)	4.80 (4.69 - 4.91)	4.59 (4.48 - 4.70)
**2021 COVID-19 cohort**				
18-24 y	1.22 (1.19 - 1.25)	1.06 (1.00 - 1.12)	1.25 (1.23 - 1.28)	1.24 (1.22 - 1.27)
25-34 y	1.71 (1.68 - 1.74)	1.31 (1.28 - 1.35)	1.73 (1.71 - 1.76)	1.68 (1.66 - 1.71)
35-44 y	2.16 (2.13 - 2.19)	1.75 (1.72 - 1.78)	2.13 (2.10 - 2.17)	2.10 (2.07 - 2.13)
45-54 y	2.26 (2.23 - 2.29)	2.19 (2.15 - 2.22)	2.27 (2.24 - 2.30)	2.26 (2.23 - 2.29)
55-64 y	2.51 (2.47 - 2.56)	2.36 (2.32 - 2.39)	2.48 (2.44 - 2.52)	2.41 (2.37 - 2.45)
**2022 COVID-19 cohort**				
18-24 y	1.41 (1.38 - 1.45)	1.44 (1.40 - 1.47)	1.53 (1.49 - 1.57)	1.47 (1.43 - 1.51)
25-34 y	1.49 (1.47 - 1.51)	1.57 (1.54 - 1.59)	1.55 (1.52 - 1.57)	1.54 (1.52 - 1.57)
35-44 y	1.52 (1.50 - 1.54)	1.60 (1.57 - 1.63)	1.53 (1.51 - 1.55)	1.55 (1.53 - 1.57)
45-54 y	1.55 (1.53 - 1.57)	1.64 (1.61 - 1.66)	1.57 (1.55 - 1.59)	1.60 (1.58 - 1.62)
55-64 y	1.60 (1.57 - 1.62)	1.74 (1.71 - 1.77)	1.56 (1.54 - 1.59)	1.63 (1.61 - 1.65)
	**COVID-19 hospitalised cohort vs 2019 pneumonia hospitalised cohort**			
	**Crude HR (95%CI)**	**Fully adjusted HR (95%CI)**		
**2020 COVID-19 hospitalised cohort**				
18-24 y	0.50 (0.39 - 0.63)	0.48 (0.37 - 0.61)		
25-34 y	0.54 (0.48 - 0.61)	0.54 (0.47 - 0.61)		
35-44 y	0.77 (0.71 - 0.84)	0.73 (0.66 - 0.80)		
45-54 y	0.94 (0.88 - 1.00)	0.81 (0.76 - 0.88)		

For peer review only

55-64 y	0.50 (0.39 - 0.63)	0.94 (0.88 - 0.99)	
**2021 COVID-19 hospitalised cohort**			
18-24 y	0.64 (0.54 - 0.75)	0.61 (0.51 - 0.72)	
25-34 y	0.57 (0.52 - 0.63)	0.57 (0.51 - 0.63)	
35-44 y	0.82 (0.76 - 0.88)	0.74 (0.68 - 0.80)	
45-54 y	1.09 (1.03 - 1.16)	0.94 (0.88 - 1.01)	
55-64 y	1.16 (1.09 - 1.22)	0.61 (0.51 - 0.72)	
**2022 COVID-19 hospitalised cohort**			
18-24 y	0.54 (0.46 - 0.63)	0.54 (0.46 - 0.64)	
25-34 y	0.42 (0.39 - 0.47)	0.44 (0.39 - 0.48)	
35-44 y	0.56 (0.52 - 0.61)	0.56 (0.52 - 0.61)	
45-54 y	0.73 (0.68 - 0.78)	0.72 (0.68 - 0.77)	
55-64 y	0.76 (0.72 - 0.80)	0.78 (0.74 - 0.82)	

*Fully adjusted models include age, sex, IMD quintile, region, ethnicity, obesity, smoking status, hypertension, diabetes, chronic respiratory disease, asthma, chronic cardiac disease, lung cancer, haematological cancer, other cancer, chronic liver disease, other neurological disease, organ transplant, asplenia, HIV, permanent immunodeficiency, and rheumatoid arthritis/systemic lupus erythematosus/psoriasis.

For peer review only

Supplementary Table 8. Crude and adjusted hazard ratio of first sick note for all comparisons, stratified by sex

	COVID-19 cohort vs 2019 general population		COVID-19 cohort vs contemporary general population	
	Crude HR (95%CI)	Fully adjusted HR (95%CI)	Crude HR (95%CI)	Fully adjusted HR (95%CI)
**2020 COVID-19 cohort**				
Female	3.39 (3.34 - 3.44)	3.40 (3.35 - 3.45)	4.22 (4.16 - 4.29)	4.22 (4.15 - 4.28)
Male	2.86 (2.80 - 2.92)	2.84 (2.78 - 2.89)	3.91 (3.83 - 4.00)	3.79 (3.71 - 3.87)
**2021 COVID-19 cohort**				
Female	2.08 (2.06 - 2.10)	2.08 (2.06 - 2.10)	2.01 (1.99 - 2.03)	2.01 (2.00 - 2.03)
Male	1.84 (1.82 - 1.86)	1.93 (1.90 - 1.95)	1.93 (1.91 - 1.95)	1.95 (1.93 - 1.98)
**2022 COVID-19 cohort**				
Female	1.54 (1.52 - 1.55)	1.52 (1.51 - 1.54)	1.53 (1.51 - 1.54)	1.53 (1.52 - 1.54)
Male	1.50 (1.48 - 1.52)	1.61 (1.59 - 1.64)	1.60 (1.58 - 1.62)	1.67 (1.65 - 1.69)
	**COVID-19 hospitalised cohort vs 2019 pneumonia hospitalised cohort**			
	**Crude HR (95%CI)**	**Fully adjusted HR (95%CI)**		
**2020 COVID-19 hospitalised cohort**				
Female	0.95 (0.91 - 1.01)	0.78 (0.72 - 0.85)		
Male	0.85 (0.81 - 0.89)	0.75 (0.69 - 0.81)		
**2021 COVID-19 hospitalised cohort**				
Female	0.87 (0.83 - 0.92)	0.76 (0.71 - 0.82)		
Male	1.02 (0.97 - 1.06)	0.88 (0.82 - 0.94)		
**2022 COVID-19 hospitalised cohort**				
Female	0.61 (0.59 - 0.64)	0.57 (0.53 - 0.61)		
Male	0.70 (0.67 - 0.74)	0.70 (0.66 - 0.75)		

*Fully adjusted models include age, sex, IMD quintile, region, ethnicity, obesity, smoking status, hypertension, diabetes, chronic respiratory disease, asthma, chronic cardiac disease, lung cancer, haematological cancer, other cancer, chronic liver disease, other neurological disease, organ transplant, asplenia, HIV, permanent immunodeficiency, and rheumatoid arthritis/systemic lupus erythematosus/psoriasis.

Supplementary Table 9. Crude and adjusted hazard ratio of first sick note for all comparisons, stratified by ethnicity

	**COVID-19 cohort vs 2019 general population**		**COVID-19 cohort vs contemporary general population**	
	**Crude HR (95%CI)**	**Fully adjusted HR (95%CI)**	**Crude HR (95%CI)**	**Fully adjusted HR (95%CI)**
**2020 COVID-19 cohort**				
White	3.13 (3.08 - 3.18)	3.13 (3.08 - 3.18)	4.05 (3.99 - 4.11)	4.01 (3.95 - 4.08)
Asian or Asian British	3.71 (3.59 - 3.84)	3.60 (3.48 - 3.73)	4.44 (4.30 - 4.60)	4.31 (4.16 - 4.47)
Black	3.81 (3.56 - 4.08)	3.76 (3.50 - 4.03)	5.12 (4.78 - 5.49)	4.95 (4.60 - 5.32)
Mixed	3.04 (2.73 - 3.38)	2.99 (2.68 - 3.33)	4.00 (3.59 - 4.45)	3.89 (3.48 - 4.34)
Other	5.33 (4.83 - 5.89)	4.54 (4.08 - 5.05)	6.74 (6.09 - 7.46)	5.89 (5.28 - 6.56)
Not stated				
**2021 COVID-19 cohort**				
White	1.89 (1.87 - 1.91)	1.94 (1.92 - 1.96)	1.91 (1.90 - 1.93)	1.94 (1.92 - 1.96)
Asian or Asian British	2.84 (2.76 - 2.92)	2.93 (2.84 - 3.02)	2.69 (2.62 - 2.76)	2.62 (2.55 - 2.70)
Black	2.59 (2.47 - 2.72)	2.88 (2.73 - 3.04)	2.54 (2.42 - 2.66)	2.62 (2.50 - 2.74)
Mixed	2.01 (1.87 - 2.15)	2.14 (1.98 - 2.30)	2.09 (1.95 - 2.23)	2.08 (1.95 - 2.23)
Other	3.42 (3.18 - 3.67)	3.22 (2.97 - 3.49)	3.23 (3.03 - 3.45)	2.90 (2.71 - 3.11)
Not stated				
**2022 COVID-19 cohort**				
White	1.46 (1.45 - 1.47)	1.48 (1.47 - 1.50)	1.49 (1.48 - 1.50)	2.10 (2.04 - 2.16)
Asian or Asian British	2.21 (2.15 - 2.28)	2.48 (2.40 - 2.58)	1.97 (1.91 - 2.02)	1.69 (1.61 - 1.77)
Black	1.94 (1.85 - 2.04)	1.88 (1.77 - 1.99)	1.71 (1.64 - 1.80)	1.60 (1.51 - 1.71)
Mixed	1.57 (1.47 - 1.68)	1.66 (1.54 - 1.79)	1.54 (1.45 - 1.64)	1.98 (1.86 - 2.10)
Other	2.32 (2.16 - 2.48)	2.23 (2.06 - 2.42)	1.93 (1.82 - 2.04)	1.61 (1.59 - 1.63)
Not stated	1.62 (1.59 - 1.64)	1.54 (1.51 - 1.57)	1.63 (1.61 - 1.66)	2.10 (2.04 - 2.16)
	**COVID-19 hospitalised cohort vs 2019 pneumonia hospitalised cohort**			
	**Crude HR (95%CI)**	**Fully adjusted HR (95%CI)**		
**2020 COVID-19 hospitalised cohort**				
White	0.93 (0.89 - 0.97)	0.78 (0.72 - 0.83)		

For peer review only

Asian or Asian British	0.98 (0.86 - 1.12)	0.90 (0.76 - 1.08)	
Black	0.97 (0.79 - 1.19)	0.80 (0.60 - 1.07)	
Mixed	0.96 (0.68 - 1.35)	0.58 (0.35 - 0.94)	
Other	0.83 (0.61 - 1.14)	0.77 (0.51 - 1.18)	
Not stated	0.83 (0.77 - 0.90)	0.74 (0.65 - 0.83)	
**2021 COVID-19 hospitalised cohort**			
White	0.99 (0.95 - 1.03)	0.81 (0.76 - 0.87)	
Asian or Asian British	0.94 (0.83 - 1.07)	1.02 (0.86 - 1.22)	
Black	1.03 (0.85 - 1.25)	0.90 (0.69 - 1.17)	
Mixed	0.83 (0.60 - 1.14)	0.55 (0.35 - 0.84)	
Other	0.70 (0.52 - 0.96)	0.73 (0.47 - 1.12)	
Not stated	0.88 (0.82 - 0.94)	0.76 (0.69 - 0.85)	
**2022 COVID-19 hospitalised cohort**			
White	0.65 (0.63 - 0.68)	0.62 (0.58 - 0.65)	
Asian or Asian British	0.75 (0.65 - 0.86)	0.75 (0.63 - 0.90)	
Black	0.72 (0.58 - 0.90)	0.68 (0.52 - 0.90)	
Mixed	0.62 (0.44 - 0.88)	0.51 (0.32 - 0.80)	
Other	0.58 (0.42 - 0.80)	0.61 (0.41 - 0.91)	
Not stated	0.61 (0.57 - 0.66)	0.60 (0.54 - 0.66)	

*Fully adjusted models include age, sex, IMD quintile, region, ethnicity, obesity, smoking status, hypertension, diabetes, chronic respiratory disease, asthma, chronic cardiac disease, lung cancer, haematological cancer, other cancer, chronic liver disease, other neurological disease, organ transplant, asplenia, HIV, permanent immunodeficiency, and rheumatoid arthritis/systemic lupus erythematosus/psoriasis.

For peer review only

Supplementary Table 10. Crude and adjusted hazard ratio of first sick note for all comparisons, stratified by IMD quintile

	COVID-19 cohort vs 2019 general population		COVID-19 cohort vs contemporary general population	
	**Crude HR (95%CI)**	**Fully adjusted HR (95%CI)**	**Crude HR (95%CI)**	**Fully adjusted HR (95%CI)**
**2020 COVID-19 cohort**				
1 (most deprived)	2.64 (2.59 - 2.70)	2.70 (2.64 - 2.76)	3.44 (3.36 - 3.51)	3.41 (3.33 - 3.48)
2	3.05 (2.98 - 3.13)	3.06 (2.98 - 3.14)	3.96 (3.86 - 4.06)	3.91 (3.81 - 4.02)
3	3.45 (3.36 - 3.54)	3.43 (3.33 - 3.52)	4.41 (4.29 - 4.53)	4.36 (4.24 - 4.49)
4	3.71 (3.61 - 3.82)	3.71 (3.61 - 3.82)	4.72 (4.59 - 4.86)	4.71 (4.57 - 4.86)
5 (least deprived)	3.86 (3.74 - 3.99)	3.93 (3.80 - 4.06)	5.02 (4.86 - 5.18)	5.15 (4.98 - 5.32)
**2021 COVID-19 cohort**				
1 (most deprived)	1.84 (1.81 - 1.86)	1.86 (1.84 - 1.89)	1.77 (1.75 - 1.79)	1.77 (1.74 - 1.79)
2	1.93 (1.90 - 1.96)	1.98 (1.94 - 2.01)	1.93 (1.90 - 1.96)	1.95 (1.92 - 1.98)
3	2.04 (2.00 - 2.07)	2.10 (2.06 - 2.14)	2.06 (2.02 - 2.09)	2.09 (2.06 - 2.13)
4	2.09 (2.05 - 2.13)	2.15 (2.10 - 2.19)	2.09 (2.05 - 2.12)	2.15 (2.11 - 2.19)
5 (least deprived)	2.18 (2.14 - 2.23)	2.25 (2.20 - 2.30)	2.21 (2.17 - 2.26)	2.30 (2.26 - 2.35)
**2022 COVID-19 cohort**				
1 (most deprived)	1.68 (1.66 - 1.70)	1.59 (1.57 - 1.62)	1.58 (1.55 - 1.60)	1.55 (1.53 - 1.57)
2	1.64 (1.62 - 1.67)	1.57 (1.54 - 1.59)	1.60 (1.58 - 1.62)	1.59 (1.56 - 1.61)
3	1.62 (1.60 - 1.65)	1.55 (1.52 - 1.58)	1.58 (1.56 - 1.61)	1.57 (1.55 - 1.59)
4	1.64 (1.62 - 1.67)	1.53 (1.49 - 1.56)	1.61 (1.59 - 1.64)	1.60 (1.58 - 1.63)
5 (least deprived)	1.62 (1.59 - 1.65)	1.50 (1.47 - 1.54)	1.56 (1.54 - 1.59)	1.56 (1.53 - 1.58)
	**COVID-19 hospitalised cohort vs 2019 pneumonia hospitalised cohort**			
	**Crude HR (95%CI)**	**Fully adjusted HR (95%CI)**		
**2020 COVID-19 hospitalised cohort**				
1 (most deprived)	0.96 (0.89 - 1.03)	0.76 (0.68 - 0.84)		
2	0.9 (0.83 - 0.97)	0.73 (0.65 - 0.82)		
3	0.93 (0.85 - 1.01)	0.83 (0.73 - 0.94)		
4	0.88 (0.81 - 0.96)	0.84 (0.73 - 0.96)		

For peer review only

5 (least deprived)	0.78 (0.70 - 0.86)	0.71 (0.60 - 0.83)	
**2021 COVID-19 hospitalised cohort**			
1 (most deprived)	1.02 (0.97 - 1.09)	0.85 (0.78 - 0.93)	
2	0.96 (0.90 - 1.03)	0.80 (0.72 - 0.89)	
3	0.96 (0.90 - 1.04)	0.89 (0.79 - 0.99)	
4	0.88 (0.81 - 0.96)	0.79 (0.70 - 0.90)	
5 (least deprived)	0.82 (0.75 - 0.90)	0.69 (0.60 - 0.79)	
**2022 COVID-19 hospitalised cohort**			
1 (most deprived)	0.71 (0.67 - 0.76)	0.66 (0.60 - 0.72)	
2	0.65 (0.61 - 0.70)	0.63 (0.57 - 0.70)	
3	0.62 (0.57 - 0.66)	0.63 (0.57 - 0.70)	
4	0.60 (0.56 - 0.65)	0.57 (0.51 - 0.64)	
5 (least deprived)	0.60 (0.55 - 0.65)	0.59 (0.52 - 0.67)	

*Fully adjusted models include age, sex, IMD quintile, region, ethnicity, obesity, smoking status, hypertension, diabetes, chronic respiratory disease, asthma, chronic cardiac disease, lung cancer, haematological cancer, other cancer, chronic liver disease, other neurological disease, organ transplant, asplenia, HIV, permanent immunodeficiency, and rheumatoid arthritis/systemic lupus erythematosus/psoriasis.

For peer review only

Supplementary Table 11. Crude and adjusted hazard ratio of first sick note for all comparisons, stratified by region

	COVID-19 cohort vs 2019 general population		COVID-19 cohort vs contemporary general population	
	Crude HR (95%CI)	Fully adjusted HR (95%CI)	Crude HR (95%CI)	Fully adjusted HR (95%CI)
**2020 COVID-19 cohort**				
East Midlands	3.13 (3.05 - 3.22)	3.12 (3.04 - 3.21)	4.05 (3.94 - 4.16)	3.98 (3.87 - 4.09)
East	3.34 (3.24 - 3.45)	3.17 (3.07 - 3.27)	4.56 (4.42 - 4.71)	4.28 (4.14 - 4.43)
London	3.68 (3.45 - 3.92)	3.24 (3.03 - 3.47)	5.22 (4.89 - 5.58)	4.57 (4.26 - 4.89)
North East	3.12 (3.00 - 3.24)	3.15 (3.03 - 3.28)	3.86 (3.71 - 4.02)	3.86 (3.71 - 4.02)
North West	3.20 (3.11 - 3.30)	3.24 (3.14 - 3.33)	3.94 (3.82 - 4.06)	3.92 (3.80 - 4.04)
South East	3.28 (3.06 - 3.52)	3.14 (2.92 - 3.37)	4.34 (4.04 - 4.65)	4.15 (3.86 - 4.46)
South West	3.44 (3.29 - 3.61)	3.36 (3.21 - 3.52)	4.55 (4.35 - 4.77)	4.40 (4.19 - 4.61)
West Midlands	3.29 (3.14 - 3.46)	3.32 (3.16 - 3.49)	4.37 (4.16 - 4.59)	4.34 (4.13 - 4.57)
Yorkshire & The Humber	3.15 (3.07 - 3.22)	3.16 (3.08 - 3.24)	4.02 (3.92 - 4.12)	3.97 (3.88 - 4.07)
**2021 COVID-19 cohort**				
East Midlands	2.05 (2.02 - 2.09)	2.06 (2.02 - 2.10)	1.95 (1.92 - 1.98)	1.99 (1.96 - 2.02)
East	2.01 (1.97 - 2.05)	1.97 (1.93 - 2.01)	2.07 (2.03 - 2.11)	2.07 (2.04 - 2.11)
London	2.63 (2.53 - 2.74)	2.62 (2.50 - 2.74)	2.69 (2.59 - 2.79)	2.60 (2.50 - 2.70)
North East	1.96 (1.91 - 2.01)	1.97 (1.91 - 2.03)	1.85 (1.80 - 1.90)	1.89 (1.84 - 1.94)
North West	1.85 (1.81 - 1.88)	1.87 (1.83 - 1.91)	1.73 (1.69 - 1.76)	1.76 (1.72 - 1.79)
South East	2.02 (1.94 - 2.10)	1.98 (1.90 - 2.07)	2.02 (1.95 - 2.09)	2.05 (1.98 - 2.12)
South West	2.03 (1.98 - 2.09)	1.99 (1.93 - 2.04)	2.02 (1.97 - 2.06)	2.06 (2.02 - 2.11)
West Midlands	2.10 (2.03 - 2.17)	2.18 (2.1 - 2.26)	2.04 (1.98 - 2.11)	2.08 (2.01 - 2.14)
Yorkshire & The Humber	2.03 (1.99 - 2.06)	2.04 (2.00 - 2.07)	1.92 (1.89 - 1.95)	1.95 (1.92 - 1.99)
**2022 COVID-19 cohort**				
East Midlands	1.71 (1.68 - 1.73)	1.58 (1.55 - 1.61)	1.55 (1.53 - 1.58)	1.57 (1.55 - 1.60)
East	1.61 (1.58 - 1.64)	1.50 (1.47 - 1.53)	1.55 (1.53 - 1.58)	1.57 (1.55 - 1.59)
London	1.91 (1.84 - 1.98)	1.96 (1.88 - 2.05)	1.79 (1.74 - 1.86)	1.90 (1.83 - 1.96)
North East	1.68 (1.63 - 1.73)	1.56 (1.51 - 1.61)	1.50 (1.46 - 1.54)	1.53 (1.49 - 1.57)
North West	1.63 (1.60 - 1.66)	1.49 (1.46 - 1.53)	1.51 (1.48 - 1.54)	1.51 (1.48 - 1.54)
South East	1.67 (1.61 - 1.73)	1.53 (1.47 - 1.59)	1.54 (1.50 - 1.58)	1.54 (1.50 - 1.58)

For peer review only

South West	1.66 (1.62 - 1.69)	1.47 (1.44 - 1.51)	1.54 (1.52 - 1.57)	1.54 (1.52 - 1.57)
West Midlands	1.80 (1.75 - 1.86)	1.74 (1.68 - 1.81)	1.62 (1.57 - 1.67)	1.65 (1.60 - 1.71)
Yorkshire & The Humber	1.65 (1.63 - 1.68)	1.56 (1.53 - 1.59)	1.53 (1.51 - 1.56)	1.58 (1.55 - 1.61)

	**COVID-19 hospitalised cohort vs 2019 pneumonia hospitalised cohort**	
	**Crude HR (95%CI)**	**Fully adjusted HR (95%CI)**
**2020 COVID-19 hospitalised cohort**		
East Midlands	0.83 (0.77 - 0.91)	0.69 (0.61 - 0.78)
East	0.78 (0.67 - 0.91)	0.74 (0.65 - 0.84)
London	0.94 (0.82 - 1.09)	0.65 (0.51 - 0.83)
North East	1.06 (0.95 - 1.18)	0.81 (0.65 - 1.02)
North West	0.75 (0.63 - 0.90)	0.89 (0.76 - 1.04)
South East	1.10 (0.98 - 1.23)	0.58 (0.45 - 0.76)
South West	0.88 (0.75 - 1.02)	1.08 (0.91 - 1.28)
West Midlands	0.96 (0.88 - 1.05)	0.74 (0.59 - 0.93)
Yorkshire & The Humber	0.83 (0.77 - 0.91)	0.78 (0.69 - 0.90)
**2021 COVID-19 hospitalised cohort**		
East Midlands	0.91 (0.85 - 0.98)	0.78 (0.69 - 0.87)
East	0.92 (0.85 - 0.99)	0.79 (0.70 - 0.88)
London	0.86 (0.74 - 1.00)	0.80 (0.65 - 1.00)
North East	0.94 (0.83 - 1.08)	0.83 (0.68 - 1.03)
North West	0.98 (0.89 - 1.09)	0.81 (0.70 - 0.95)
South East	0.90 (0.78 - 1.04)	0.79 (0.63 - 0.99)
South West	0.99 (0.90 - 1.09)	0.82 (0.71 - 0.95)
West Midlands	0.96 (0.84 - 1.11)	0.92 (0.74 - 1.15)
Yorkshire & The Humber	0.98 (0.91 - 1.06)	0.86 (0.77 - 0.97)
**2022 COVID-19 hospitalised cohort**		
East Midlands	0.68 (0.63 - 0.73)	0.68 (0.61 - 0.75)
East	0.60 (0.56 - 0.65)	0.60 (0.54 - 0.66)
London	0.61 (0.52 - 0.71)	0.72 (0.58 - 0.90)
North East	0.59 (0.52 - 0.68)	0.58 (0.48 - 0.70)

For peer review only

North West	0.69 (0.63 - 0.77)	0.61 (0.53 - 0.71)	
South East	0.58 (0.51 - 0.66)	0.56 (0.46 - 0.67)	
South West	0.63 (0.58 - 0.69)	0.58 (0.51 - 0.67)	
West Midlands	0.74 (0.64 - 0.86)	0.72 (0.58 - 0.88)	
Yorkshire & The Humber	0.69 (0.64 - 0.75)	0.61 (0.54 - 0.68)	

*Fully adjusted models include age, sex, IMD quintile, region, ethnicity, obesity, smoking status, hypertension, diabetes, chronic respiratory disease, asthma, chronic cardiac disease, lung cancer, haematological cancer, other cancer, chronic liver disease, other neurological disease, organ transplant, asplenia, HIV, permanent immunodeficiency, and rheumatoid arthritis/systemic lupus erythematosus/psoriasis.

For peer review only

Supplementary Figure 3. Adjusted hazard ratio of first sick note comparing hospitalised COVID-19 cohorts to people hospitalised with pneumonia in 2019, stratified by demographic categories and year

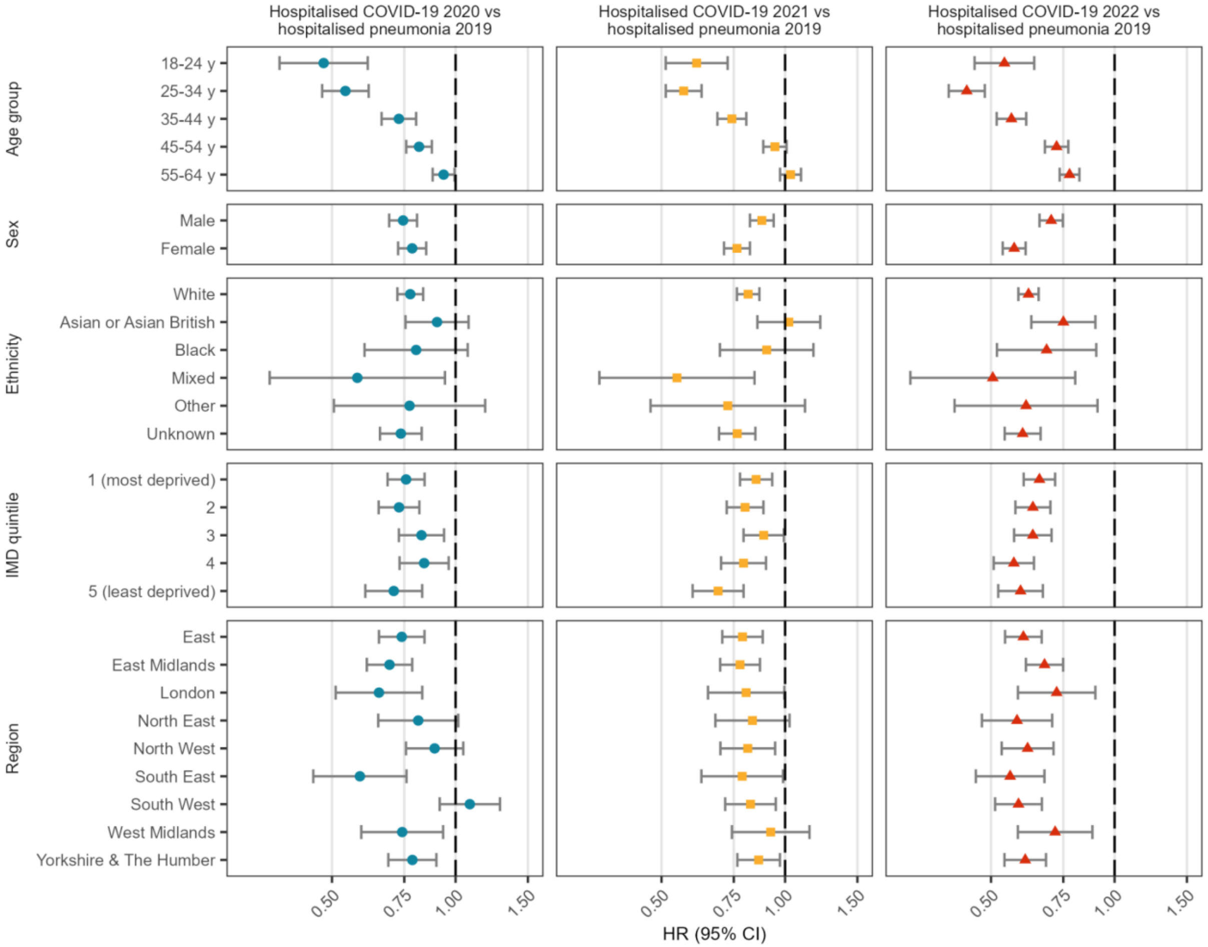

*All models are adjusted for age, sex, ethnicity, IMD quintile and region, excluding the stratification variable. Models are additionally adjusted for obesity, smoking status, hypertension, diabetes, chronic respiratory disease, asthma, chronic cardiac disease, lung cancer, haematological cancer, other cancer, chronic liver disease, other neurological disease, organ transplant, asplenia, HIV, permanent immunodeficiency, and rheumatoid arthritis/systemic lupus erythematosus/psoriasis

Information governance

NHS England is the data controller of the NHS England OpenSAFELY COVID-19 Service; TPP is the data processor; all study authors using OpenSAFELY have the approval of NHS England.(1) This implementation of OpenSAFELY is hosted within the TPP environment which is accredited to the ISO 27001 information security standard and is NHS IG Toolkit compliant.(2)

Patient data has been pseudonymised for analysis and linkage using industry standard cryptographic hashing techniques; all pseudonymised datasets transmitted for linkage onto OpenSAFELY are encrypted; access to the NHS England OpenSAFELY COVID-19 service is via a virtual private network (VPN) connection; the researchers hold contracts with NHS England and only access the platform to initiate database queries and statistical models; all database activity is logged; only aggregate statistical outputs leave the platform environment following best practice for anonymisation of results such as statistical disclosure control for low cell counts.(3)

The service adheres to the obligations of the UK General Data Protection Regulation (UK GDPR) and the Data Protection Act 2018. The service previously operated under notices initially issued in February 2020 by the the Secretary of State under Regulation 3(4) of the Health Service (Control of Patient Information) Regulations 2002 (COPI Regulations), which required organisations to process confidential patient information for COVID-19 purposes; this set aside the requirement for patient consent.(4) As of 1 July 2023, the Secretary of State has requested that NHS England continue to operate the Service under the COVID-19 Directions 2020.(5) In some cases of data sharing, the common law duty of confidence is met using, for example, patient consent or support from the Health Research Authority Confidentiality Advisory Group.(6)

Taken together, these provide the legal bases to link patient datasets using the service. GP practices, which provide access to the primary care data, are required to share relevant health information to support the public health response to the pandemic, and have been informed of how the service operates.

(1) NHS Digital. The NHS England OpenSAFELY COVID-19 service - privacy notice [Internet]. 2023 [cited 2023 Jul 5]. Available from: https://digital.nhs.uk/coronavirus/coronavirus-covid-19-response-information-governance-hub/the-nhs-england-opensafely-covid-19-service-privacy-notice

(2) NHS Digital. NHS Digital. 2023 [cited 2023 Jul 5]. Data Security and Protection Toolkit. Available from: https://digital.nhs.uk/data-and-information/looking-after-information/data-security-and-information-governance/data-security-and-protection-toolkit

(3) NHS Digital [Internet]. [cited 2023 Mar 6]. ISB1523: Anonymisation Standard for Publishing Health and Social Care Data. Available from: https://digital.nhs.uk/data-and-information/information-standards/information-standards-and-data-collections-including-extractions/publications-and-notifications/standards-and-collections/isb1523-anonymisation-standard-for-publishing-health-and-social-care-data

(4) UK Department of Health and Social Care. GOV.UK. 2022 [cited 2023 Jul 5]. [Withdrawn] Coronavirus (COVID-19): notice under regulation 3(4) of the Health Service (Control of Patient Information) Regulations 2002 – general. Available from: https://www.gov.uk/government/publications/coronavirus-covid-19-

notification-of-data-controllers-to-share-information/coronavirus-covid-19-notice-under-regulation-34-of-the-health-service-control-of-patient-information-regulations-2002-general--2

(5) NHS Digital. NHS Digital. 2022 [cited 2023 Jul 5]. Secretary of State for Health and Social Care: COVID-19 Public Health Directions 2020. Available from: https://digital.nhs.uk/about-nhs-digital/corporate-information-and-documents/directions-and-data-provision-notices/secretary-of-state-directions/covid-19-public-health-directions-2020

(6) NHS Health Research Authority. Health Research Authority. [cited 2023 Jul 5]. Confidentiality Advisory Group. Available from: https://www.hra.nhs.uk/about-us/committees-and-services/confidentiality-advisory-group/

For peer review only

The RECORD statement – checklist of items, extended from the STROBE statement, that should be reported in observational studies using routinely collected health data.

	Item No.	STROBE items	Location in manuscript where items are reported	RECORD items	Location in manuscript where items are reported
**Title and abstract**					
	1	(a) Indicate the study's design with a commonly used term in the title or the abstract (b) Provide in the abstract an informative and balanced summary of what was done and what was found		RECORD 1.1: The type of data used should be specified in the title or abstract. When possible, the name of the databases used should be included.<br><br>RECORD 1.2: If applicable, the geographic region and timeframe within which the study took place should be reported in the title or abstract.<br><br>RECORD 1.3: If linkage between databases was conducted for the study, this should be clearly stated in the title or abstract.	1.1<br>Title<br>Page 6 (Data Source and Data Sharing)<br><br>1.2 Abstract
**Introduction**					
Background rationale	2	Explain the scientific background and rationale for the investigation being reported			Background
Objectives	3	State specific objectives, including any prespecified hypotheses			Last paragraph of Background
**Methods**					
Study Design	4	Present key elements of study design early in the paper			Title<br>Methods - Study design
Setting	5	Describe the setting, locations, and relevant dates, including periods of recruitment, exposure, follow-up, and data collection			Methods - Study population)<br>Methods - Study measures

For peer review only

Participants	6	*(a) Cohort study* - Give the eligibility criteria, and the sources and methods of selection of participants. Describe methods of follow-up<br>*Case-control study* - Give the eligibility criteria, and the sources and methods of case ascertainment and control selection. Give the rationale for the choice of cases and controls<br>*Cross-sectional study* - Give the eligibility criteria, and the sources and methods of selection of participants<br><br>*(b) Cohort study* - For matched studies, give matching criteria and number of exposed and unexposed<br>*Case-control study* - For matched studies, give matching criteria and the number of controls per case		RECORD 6.1: The methods of study population selection (such as codes or algorithms used to identify subjects) should be listed in detail. If this is not possible, an explanation should be provided.<br><br>RECORD 6.2: Any validation studies of the codes or algorithms used to select the population should be referenced. If validation was conducted for this study and not published elsewhere, detailed methods and results should be provided.<br><br>RECORD 6.3: If the study involved linkage of databases, consider use of a flow diagram or other graphical display to demonstrate the data linkage process, including the number of individuals with linked data at each stage.	6.1 A link to all code and codelists used in the study is provided in the Methods<br><br>6.2 N/A<br><br>6.3 N/A
Variables	7	Clearly define all outcomes, exposures, predictors, potential confounders, and effect modifiers. Give diagnostic criteria, if applicable.		RECORD 7.1: A complete list of codes and algorithms used to classify exposures, outcomes, confounders, and effect modifiers should be provided. If these cannot be reported, an explanation should be provided.	A link to all code and codelists used in the study is provided in the Methods
Data sources/ measurement	8	For each variable of interest, give sources of data and details of methods of assessment (measurement).<br>Describe comparability of assessment methods if there is more than one group			Methods - Study measures;<br>A link to all code and codelists used in the study is provided in the Methods

For peer review only - http://bmjopen.bmj.com/site/about/guidelines.xhtml

Bias	9	Describe any efforts to address potential sources of bias			Methods – Study population<br>Methods - Cox regression<br>Discussion – Strengths and Weaknesses
Study size	10	Explain how the study size was arrived at			Methods - Study population
Quantitative variables	11	Explain how quantitative variables were handled in the analyses. If applicable, describe which groupings were chosen, and why			Methods - Cox regression
Statistical methods	12	(a) Describe all statistical methods, including those used to control for confounding<br>(b) Describe any methods used to examine subgroups and interactions<br>(c) Explain how missing data were addressed<br>(d) *Cohort study* - If applicable, explain how loss to follow-up was addressed<br>*Case-control study* - If applicable, explain how matching of cases and controls was addressed<br>*Cross-sectional study* - If applicable, describe analytical methods taking account of sampling strategy<br>(e) Describe any sensitivity analyses			Methods - Statistical methods
Data access and cleaning methods		..		RECORD 12.1: Authors should describe the extent to which the investigators had access to the database	Data access and verification

For peer review only

				population used to create the study population.<br><br>RECORD 12.2: Authors should provide information on the data cleaning methods used in the study.	
Linkage		..		RECORD 12.3: State whether the study included person-level, institutional-level, or other data linkage across two or more databases. The methods of linkage and methods of linkage quality evaluation should be provided.	Methods - Data source
**Results**					
Participants	13	(a) Report the numbers of individuals at each stage of the study (*e.g.*, numbers potentially eligible, examined for eligibility, confirmed eligible, included in the study, completing follow-up, and analysed)<br>(b) Give reasons for non-participation at each stage.<br>(c) Consider use of a flow diagram		RECORD 13.1: Describe in detail the selection of the persons included in the study (*i.e.*, study population selection) including filtering based on data quality, data availability and linkage. The selection of included persons can be described in the text and/or by means of the study flow diagram.	Results - Study population description
Descriptive data	14	(a) Give characteristics of study participants (*e.g.*, demographic, clinical, social) and information on exposures and potential confounders<br>(b) Indicate the number of participants with missing data for each variable of interest<br>(c) *Cohort study* - summarise follow-up time (*e.g.*, average and total amount)			Results - Study population description)<br>Table 1<br>Supplementary Table 1

For peer review only

Outcome data	15	*Cohort study* - Report numbers of outcome events or summary measures over time<br>*Case-control study* - Report numbers in each exposure category, or summary measures of exposure<br>*Cross-sectional study* - Report numbers of outcome events or summary measures			Results - Overall fit note rates<br>Supplementary Tables
Main results	16	(a) Give unadjusted estimates and, if applicable, confounder-adjusted estimates and their precision (e.g., 95% confidence interval). Make clear which confounders were adjusted for and why they were included<br>(b) Report category boundaries when continuous variables were categorized<br>(c) If relevant, consider translating estimates of relative risk into absolute risk for a meaningful time period			Table 2<br>Supplementary Table 4<br>Figure 2
Other analyses	17	Report other analyses done—e.g., analyses of subgroups and interactions, and sensitivity analyses			
**Discussion**					
Key results	18	Summarise key results with reference to study objectives			Discussion - Summary
Limitations	19	Discuss limitations of the study, taking into account sources of potential bias or imprecision. Discuss both direction and magnitude of any potential bias		RECORD 19.1: Discuss the implications of using data that were not created or collected to answer the specific research question(s). Include discussion of misclassification bias, unmeasured confounding, missing data, and changing eligibility over	Discussion - Strengths and weaknesses

For peer review only

				time, as they pertain to the study being reported.	
Interpretation	20	Give a cautious overall interpretation of results considering objectives, limitations, multiplicity of analyses, results from similar studies, and other relevant evidence			Discussion - Policy implications and interpretation
Generalisability	21	Discuss the generalisability (external validity) of the study results			Discussion - Strengths and weaknesses
**Other Information**					
Funding	22	Give the source of funding and the role of the funders for the present study and, if applicable, for the original study on which the present article is based			Methods – Role of the funding source<br>Funding
Accessibility of protocol, raw data, and programming code		..		RECORD 22.1: Authors should provide information on how to access any supplemental information such as the study protocol, raw data, or programming code.	A link to the study's Github repo, including protocol, code and codelists, is provided in the Methods

*Reference: Benchimol EI, Smeeth L, Guttmann A, Harron K, Moher D, Petersen I, Sørensen HT, von Elm E, Langan SM, the RECORD Working Committee. The REporting of studies Conducted using Observational Routinely-collected health Data (RECORD) Statement. *PLoS Medicine* 2015; in press.

*Checklist is protected under Creative Commons Attribution (CC BY) license.
